# Supplementary material for: Multimorbidity clustering of the emergency department patient flow: Impact analysis of new unscheduled care clinics
Source: PLoS One. 2022 Jan 31;17(1):e0262914. doi: 10.1371/journal.pone.0262914 (PMC8803184; doi:10.1371/journal.pone.0262914)
Supplement: S1 Table — (DOCX) [file pone.0262914.s001.docx]

**S1 Table.** Diagnostic content and quality indicators of the 14 clusters

| **Cluster name** | **Visits: n** | **Composition (blocks of diagnoses)** | **RR_intra_*** | **RR_inter_*** | **MR*** |
| --- | --- | --- | --- | --- | --- |
| **1 Digestive disorders, pregnancy and menstruation** | 17,705 | R10-R19 (37.1%); K55-K64 (16.2%); N80-N98 (8.7%); N20-N23 (6.9%); O00-O08 (5.3%); N30-N39 (4.9%); Z30-Z39 (4.1%); O20-O29 (3.8%); K80-K87 (3.8%); N10-N16 (3.7%); N70-N77 (2.1%); K35-K38 (1.3%); J30-J39 (0.6%); P90-P96 (0.6%); N60-N64 (0.5%); O85-O92 (0.3%); A50-A64 (0.3%); O60-O75 (0.2%); Q50-Q56 (0.1%) | 1.48 | 0.77 | 1.91 |
| **2 General symptoms and mental disorders** | 14,336 | R50-R69 (68.4%); G40-G47 (11.1%); R25-R29 (7.7%); I60-I69 (6.0%); H80-H83 (5.2%); Z55-Z65 (0.9%); F00-F09 (0.6%); R47-R49 (0.3%) | 1.00 | 0.80 | 1.25 |
| **3 Infectious diseases** | 14,336 | J00-J06 (21.7%); Z70-Z76 (13.8%); J40-J47 (8.6%); H65-H75 (7.8%); J20-J22 (7.4%); A00-A09 (7.3%); K50-K52 (6.1%); T66-T78 (4.4%); K20-K31 (4.2%); L50-L54 (3.6%); B00-B09 (3.4%); R20-R23 (3.4%); L20-L30 (2.2%); H60-H62 (1.8%); B35-B49 (1.6%); H90-H95 (1.1%); B25-B34 (0.9%); B85-B89 (0.5%); P80-P83 (0.1%); P75-P78 (0.1%) | 1.48 | 0.82 | 1.79 |
| **4 General symptoms of chronic conditions** | 13,893 | R00-R09 (48.4%); J09-J18 (12.8%); I30-I52 (7.9%); E79-E90 (3.5%); D60-D64 (2.6%); D50-D53 (2.5%); E10-E16 (2.3%); I20-I25 (2.2%); N17-N19 (2.0%); I26-I28 (1.8%); K90-K93 (1.6%); J95-J99 (1.5%); I10-I15 (1.5%); D55-D59 (1.4%); K40-K46 (1.3%); G50-G59 (1.3%); J60-J70 (1.1%); J80-J84 (0.9%); K70-K77 (0.7%); R70-R79 (0.6%); I95-I99 (0.6%); G90-G99 (0.5%); K65-K67 (0.3%); H30-H36 (0.3%); L40-L45 (0.2%); B15-B19 (0.2%); B65-B83 (0.2%); C76-C80 (0.1%); G60-G64 (0.1%); M91-M94 (0.1%); D00-D09 (0.1%); R90-R94 (0.1%); R95-R99 (0.1%) | 1.36 | 0.97 | 1.40 |
| **5 Mental disorders and at-risk behaviors** | 12,139 | R40-R46 (17.9%); Z00-Z13 (15.8%); S50-S59 (15.4%); F10-F19 (11.0%); F40-F49 (10.3%); T36-T50 (8.6%); F30-F39 (7.9%); T51-T65 (3.4%); F20-F29 (3.3%); S10-S19 (3.3%); F90-F98 (2.1%); F60-F69 (0.5%); C81-C96 (0.3%); L10-L14 (0.2%); F80-F89 (0.1%) | 1.51 | 0.91 | 1.65 |
| **6 Wrist and Hand Trauma** | 6,777 | S60-S69 (100.0%) | 1.00 | 0.57 | 1.75 |
| **7 Head Trauma** | 6,112 | S00-S09 (100.0%) | 1.00 | 0.44 | 2.27 |
| **8 Hip related trauma and disorders** | 5,781 | S70-S79 (21.2%); R30-R39 (14.6%); A30-A49 (11.0%); T20-T32 (9.7%); N40-N51 (9.3%); I80-I89 (7.7%); T79-T79 (6.6%); T80-T88 (5.8%); I70-I79 (3.6%); Z40-Z54 (3.3%); D65-D69 (2.4%); Z80-Z99 (1.2%); D37-D48 (1.0%); C15-C26 (0.7%); D10-D36 (0.7%); H20-H22 (0.4%); G35-G37 (0.3%); E28-E30 (0.3%); M95-M99 (0.2%) | 1.90 | 1.03 | 1.85 |
| **9 Feet Trauma** | 5,224 | S90-S99 (98.5%); M80-M90 (1.5%) | 2.18 | 0.60 | 3.65 |
| **10 Back and Spine disorders** | 3,777 | M40-M54 (79.7%); S30-S39 (20.4%) | 1.26 | 0.61 | 2.06 |
| **11 Occulomotor disorders** | 3,235 | T15-T19 (37.0%); H10-H13 (22.6%); H55-H59 (15.9%); H53-H54 (9.0%); H15-H19 (6.6%); H00-H06 (5.3%); B50-B64 (1.0%); H43-H45 (1.0%); G70-G73 (0.9%); G80-G83 (0.5%); N00-N08 (0.4%) | 1.29 | 0.52 | 2.47 |
| **12 Lower limb trauma** | 3,074 | S80-S89 (100.0%) | 1.00 | 0.64 | 1.56 |
| **13 Cutaneous infections, wounds and skin disorders** | 2,787 | L00-L08 (38.9%); Z20-Z29 (22.5%); K00-K14 (21.1%); L60-L75 (4.6%); L80-L99 (2.7%); F50-F59 (2.2%); O30-O48 (1.7%); G20-G26 (1.7%); E00-E07 (1.2%); G00-G09 (1.0%); A20-A28 (0.8%); L55-L59 (0.7%); A65-A69 (0.5%); A80-A89 (0.4%) | 1.75 | 0.80 | 2.18 |
| **14 Arthropathies** | 2,760 | M60-M79 (70.9%); M00-M25 (29.2%) | 1.88 | 0.71 | 2.63 |
| **15 Shoulder and arm trauma** | 1,930 | S40-S49 (100.0%) | 1.00 | 0.55 | 1.82 |
| **16 Chest trauma and other diseases of the pleura** | 1,151 | S20-S29 (81.1%); J90-J94 (18.9%) | 2.79 | 0.82 | 3.42 |

*RR_intra_, RR_inter_ and MR are quality indicators of the clusters. They are described in the method section and in the original article of the clustering method [1].

1. Wartelle A, Mourad-Chehade F, Yalaoui F, Chrusciel J, Laplanche D, Sanchez S. Clustering of a Health Dataset Using Diagnosis Co-Occurrences. Appl Sci. 2021;11:2373. doi:10.3390/app11052373.
