# Supplementary material for: Multimorbidity clustering of the emergency department patient flow: Impact analysis of new unscheduled care clinics
Source: PLoS One. 2022 Jan 31;17(1):e0262914. doi: 10.1371/journal.pone.0262914 (PMC8803184; doi:10.1371/journal.pone.0262914)
Supplement: S2 Table — (DOCX) [file pone.0262914.s002.docx]

**S2 Table.** Labels of the ICD10 Blocks with content of each block from the ICD10 chapter classification structure

| **ICD10 Block** | **Label** |  |
| --- | --- | --- |
| **A00A09** | Intestinal infectious diseases | |
| **A20A28** | Certain zoonotic bacterial diseases | |
| **A30A49** | Other bacterial diseases | |
| **A50A64** | Infections with a predominantly sexual mode of transmission | |
| **A65A69** | Other spirochetal diseases | |
| **A80A89** | Viral infections of the central nervous system | |
| **B00B09** | Viral infections characterized by skin and mucous membrane lesions | |
| **B15B19** | Viral hepatitis | |
| **B25B34** | Other viral diseases | |
| **B35B49** | Mycoses |  |
| **B50B64** | Protozoal diseases | |
| **B65B83** | Helminthiases | |
| **B85B89** | Pediculosis, acariasis and other infestations | |
| **C15C26** | Malignant neoplasms, digestive organs | |
| **C76C80** | Malignant neoplasms, secondary and ill-defined | |
| **C81C96** | Malignant neoplasms, stated or presumed to be primary, of lymphoid, hematopoietic and related tissue | |
| **D00D09** | In situ neoplasms | |
| **D10D36** | Benign neoplasms | |
| **D37D48** | Neoplasms of uncertain or unknown behavior | |
| **D50D53** | Nutritional anemia | |
| **D55D59** | Hemolytic anemia | |
| **D60D64** | Aplastic and other anemia | |
| **D65D69** | Coagulation defects, purpura and other hemorrhagic conditions | |
| **E00E07** | Thyroid gland/Thyroid | |
| **E10E16** | Pancreas/Insulin and glucagon | |
| **E28E30** | Gonads/Estrogen, androgens, testosterone, etc. | |
| **E79E90** | Other metabolic disorders | |
| **F00F09** | Organic, including symptomatic mental disorders | |
| **F10F19** | Mental and behavioral disorders due to psychoactive substance use | |
| **F20F29** | Schizophrenia, schizotypal and delusional disorders | |
| **F30F39** | Mood (affective) disorders | |
| **F40F49** | Neurotic, stress-related and somatoform disorders | |
| **F50F59** | Behavioral syndromes associated with physiological disturbances and physical factors | |
| **F60F69** | Disorders of adult personality and behavior | |
| **F80F89** | Disorders of psychological development | |
| **F90F98** | Behavioral and emotional disorders with onset usually occurring in childhood and adolescence | |
| **G00G09** | Inflammatory diseases of the central nervous system | |
| **G20G26** | Extrapyramidal and movement disorders | |
| **G35G37** | Demyelinating diseases of the central nervous system | |
| **G40G47** | Episodic and paroxysmal disorders | |
| **G50G59** | Nerve, nerve root and plexus disorders | |
| **G60G64** | Polyneuropathies and other disorders of the peripheral nervous system | |
| **G70G73** | Diseases of myoneural junction and muscle | |
| **G80G83** | Cerebral palsy and other paralytic syndromes | |
| **G90G99** | Other disorders of the nervous system | |
| **H00H06** | Disorders of eyelid, lacrimal system and orbit | |
| **H10H13** | Disorders of conjunctiva | |
| **H15H19** | Disorders of sclera and cornea | |
| **H20H22** | Disorders of iris and ciliary body | |
| **H30H36** | Disorders of choroid and retina | |
| **H43H45** | Disorders of vitreous body and globe | |
| **H53H54** | Disorders of ocular muscles, binocular movement, accommodation and refraction | |
| **H55H59** | Other disorders of eye and adnexa | |
| **H60H62** | Diseases of external ear | |
| **H65H75** | Diseases of middle ear and mastoid | |
| **H80H83** | Diseases of inner ear | |
| **H90H95** | Other disorders of ear | |
| **I10I15** | Hypertensive diseases | |
| **I20I25** | Ischemic heart diseases | |
| **I26I28** | Pulmonary heart disease and diseases of pulmonary circulation | |
| **I30I52** | Other forms of heart disease | |
| **I60I69** | Cerebrovascular diseases | |
| **I70I79** | Diseases of arteries, arterioles and capillaries | |
| **I80I89** | Diseases of veins, lymphatic vessels and lymph nodes, not elsewhere classified | |
| **I95I99** | Other and unspecified disorders of the circulatory system | |
| **J00J06** | Acute upper respiratory infections | |
| **J09J18** | Influenza and Pneumonia | |
| **J20J22** | Other acute lower respiratory infections | |
| **J30J39** | Other diseases of upper respiratory tract | |
| **J40J47** | Chronic lower respiratory diseases | |
| **J60J70** | Lung diseases due to external agents | |
| **J80J84** | Other respiratory diseases principally affecting the interstitium | |
| **J90J94** | Other diseases of pleura | |
| **J95J99** | Other diseases of the respiratory system | |
| **K00K14** | Diseases of oral cavity, salivary glands and jaws | |
| **K20K31** | Diseases of esophagus, stomach and duodenum | |
| **K35K38** | Diseases of appendix | |
| **K40K46** | Hernia |  |
| **K50K52** | Noninfective enteritis and colitis | |
| **K55K64** | Other diseases of intestines | |
| **K65K67** | Diseases of peritoneum | |
| **K70K77** | Diseases of liver | |
| **K80K87** | Disorders of gallbladder, biliary tract and pancreas | |
| **K90K93** | Other diseases of the digestive system | |
| **L00L08** | Infections of the skin and subcutaneous tissue | |
| **L10L14** | Bullous disorders | |
| **L20L30** | Dermatitis and eczema | |
| **L40L45** | Papulosquamous disorders | |
| **L50L54** | Urticaria and erythema | |
| **L55L59** | Radiation-related disorders of the skin and subcutaneous tissue | |
| **L60L75** | Radiation-related disorders of the skin and subcutaneous tissue | |
| **L80L99** | Other disorders of the skin and subcutaneous tissue | |
| **M00M25** | Arthropathies | |
| **M40M54** | Dorsopathies | |
| **M60M79** | Soft tissue disorders | |
| **M80M90** | Osteopathies | |
| **M91M94** | Chondropathies | |
| **M95M99** | Other disorders of the musculoskeletal system and connective tissue | |
| **N00N08** | Glomerular diseases | |
| **N10N16** | Renal tubulo-interstitial diseases | |
| **N17N19** | Renal failure | |
| **N20N23** | Urolithiasis |  |
| **N30N39** | Other diseases of urinary system | |
| **N40N51** | Diseases of male genital organs | |
| **N60N64** | Disorders of breast | |
| **N70N77** | Inflammatory diseases of female pelvic organs | |
| **N80N98** | Noninflammatory disorders of female genital tract | |
| **O00O08** | Pregnancy with abortive outcome | |
| **O20O29** | Other maternal disorders predominantly related to pregnancy | |
| **O30O48** | Maternal care related to the fetus and amniotic cavity and possible delivery problems | |
| **O60O75** | Complications of labor and delivery | |
| **O85O92** | Complications predominantly related to the puerperium | |
| **P75P78** | Digestive system disorders of fetus and newborn | |
| **P80P83** | Conditions involving the integument and temperature regulation of fetus and newborn | |
| **P90P96** | Other disorders originating in the perinatal period | |
| **Q50Q56** | Congenital malformations and deformations: genital organs | |
| **R00R09** | Circulatory and respiratory systems | |
| **R10R19** | Digestive system and abdomen | |
| **R20R23** | Skin and subcutaneous tissue | |
| **R25R29** | Nervous and musculoskeletal systems | |
| **R30R39** | Urinary system | |
| **R40R46** | Cognition, perception, emotional state and behavior | |
| **R47R49** | Speech and voice | |
| **R50R69** | General symptoms and signs | |
| **R70R79** | On examination of blood, without diagnosis | |
| **R90R94** | On diagnostic imaging and in function studies, without diagnosis | |
| **R95R99** | Ill-defined and unknown causes of mortality | |
| **S00S09** | Injury: Head | |
| **S10S19** | Injury: Neck |  |
| **S20S29** | Injury: Thorax | |
| **S30S39** | Injury: Abdomen, lower back, lumbar spine and pelvis | |
| **S40S49** | Injury: Shoulder and upper arm | |
| **S50S59** | Injury: Elbow and forearm | |
| **S60S69** | Injury: Wrist and hand | |
| **S70S79** | Injury: Hip and thigh | |
| **S80S89** | Injury: Knee and lower leg | |
| **S90S99** | Injury: Ankle and foot | |
| **T15T19** | Effects of foreign body entering through natural orifice | |
| **T20T32** | Burns and corrosions | |
| **T36T50** | Poisoning by drugs, medicaments and biological substances | |
| **T51T65** | Toxic effects of substances chiefly non-medicinal as to source | |
| **T66T78** | Other and unspecified effects of external causes | |
| **T79T79** | Certain early complications of trauma | |
| **T80T88** | Complications of surgical and medical care, not elsewhere classified | |
| **Z00Z13** | Persons encountering health services for examination and investigation | |
| **Z20Z29** | Persons with potential health hazards related to communicable diseases | |
| **Z30Z39** | Persons encountering health services in circumstances related to reproduction | |
| **Z40Z54** | Persons encountering health services for specific procedures and health care | |
| **Z55Z65** | Persons with potential health hazards related to socioeconomic and psychosocial circumstances | |
| **Z70Z76** | Persons encountering health services in other circumstances | |
| **Z80Z99** | Persons with potential health hazards related to family and personal history and certain conditions influencing health status | |
