# Supplementary material for: Multimorbidity clustering of the emergency department patient flow: Impact analysis of new unscheduled care clinics
Source: PLoS One. 2022 Jan 31;17(1):e0262914. doi: 10.1371/journal.pone.0262914 (PMC8803184; doi:10.1371/journal.pone.0262914)
Supplement: S3 Table — (DOCX) [file pone.0262914.s003.docx]

**S3 Table.** Coefficients results Poisson regression model without global trend of emergency department (ED) visits counts per day from 2016 to 2019

| **Cluster names** |  | 1: Digestive disorders, pregnancy, menstruation | 2: General symptoms and mental disorders | 3: Infectious diseases | 4: General symptoms of chronic conditions | 5: Mental disorders and at-risk behaviors | 6: Wrist and Hand Trauma | 7: Head Trauma | 8: Hip related trauma and disorders | 9: Feet Trauma |
| --- | --- | --- | --- | --- | --- | --- | --- | --- | --- | --- |
| **Patient population count (2016 to 2019)** |  | 30,166 | 29,388 | 24,512 | 25,893 | 22,100 | 13,990 | 13,595 | 11,955 | 10,514 |
| **Weekly intercept** |  | 150.34 | 122.51 | 160.41 | 141.57 | 102.68 | 61.77 | 65.65 | 50.76 | 45.97 |
| **Daily seasonal effect of month** | Jan. | 0.00 | 0.00 | 0.00 | 0.00 | 0.00 | 0.00 | 0.00 | 0.00 | 0.00 |
|  | Feb. | 1.95 | 0.97 | -1.43 | -2.25 | 0.77 | -0.58 | 0.03 | 0.61 | -0.23 |
|  | Mar. | 2.61 | 0.93 | -1.51 | -2.04 | 2.24 | 0.72 | 0.37 | 0.53 | 1.14 |
|  | Apr. | 1.58 | -0.07 | -1.80 | -4.40 | 2.36 | 1.34 | 1.03 | 1.08 | 1.48 |
|  | May. | 1.73 | 1.43 | -7.48 | -5.16 | 2.98 | 1.29 | 1.01 | 0.95 | 2.31 |
|  | Jun. | 1.00 | 1.39 | -4.17 | -5.56 | 4.66 | 1.36 | 0.96 | 0.84 | 1.88 |
|  | Jul. | 0.68 | 2.02 | -4.52 | -6.29 | 4.82 | 1.03 | 1.29 | 0.69 | 1.45 |
|  | Aug. | 0.93 | 1.34 | -7.55 | -7.07 | 2.84 | -0.32 | -0.32 | 1.04 | 0.94 |
|  | Sep. | 0.85 | 1.04 | -5.48 | -5.81 | 2.59 | 1.12 | 0.92 | 0.77 | 1.64 |
|  | Oct. | 0.84 | 1.79 | -2.68 | -4.73 | 1.60 | 1.04 | 1.10 | 1.01 | 1.64 |
|  | Nov. | 0.70 | 1.42 | -2.70 | -3.92 | 1.22 | 0.66 | 1.05 | 0.41 | 1.11 |
|  | Dec. | 1.70 | 1.14 | 6.98 | -0.90 | 1.28 | -0.15 | 0.66 | 1.03 | -0.43 |
| **Daily seasonal effect of day of the week** | Mon. | 0.00 | 0.00 | 0.00 | 0.00 | 0.00 | 0.00 | 0.00 | 0.00 | 0.00 |
|  | Tue. | 4.56 | 2.79 | 0.68 | 4.85 | -0.96 | 0.74 | -1.62 | 0.23 | 0.34 |
|  | Wed. | 1.80 | 2.04 | -0.96 | 2.79 | -1.13 | -0.16 | -1.76 | -0.06 | -0.28 |
|  | Thu. | 2.13 | 1.21 | -0.45 | 2.74 | -0.85 | -0.04 | -2.46 | -0.15 | -0.38 |
|  | Fri. | 2.17 | 1.87 | -1.17 | 3.07 | -0.31 | 0.27 | -1.45 | -0.74 | 0.03 |
|  | Sat. | 1.61 | 1.12 | -0.73 | 3.76 | 0.15 | 0.17 | -1.35 | 0.68 | -0.49 |
|  | Sun. | 0.61 | 1.06 | -0.62 | 1.70 | 0.02 | 0.25 | -1.07 | 0.28 | -0.35 |
| **Weekly effect before Period 1** | 2016/01/01 to 2017/05/01 | -21.03 | 0.74 | -21.03 | -13.34 | 2.93 | -5.80 | -3.16 | -3.94 | -5.32 |
| **95% CI before Period 1** |  | [-25.34,-16.71] | [-3.26,4.74] | [-24.72,-17.33] | [-17.08,-9.60] | [-0.73,6.59] | [-8.52,-3.08] | [-5.76,-0.55] | [-6.43,-1.45] | [-7.72,-2.93] |
| **Weekly effect before Period 2** | 2017/05/01 to 2018-10-08 | 0.00 | 0.00 | 0.00 | 0.00 | 0.00 | 0.00 | 0.00 | 0.00 | 0.00 |
| **Weekly effect after Period 3** | 2018/10/08 to 2019/04/28 | -5.11 | -8.40 | -4.09 | 3.30 | 3.65 | -8.39 | -6.21 | -1.99 | -8.00 |
| **95% CI after Period 3** |  | [-11.12,0.94] | [-13.74,-3.03] | [-9.63,1.50] | [-2.19,8.83] | [-1.26,8.59] | [-11.98,-4.76] | [-9.67,-2.71] | [-5.39,1.45] | [-11.07,-4.89] |
| **Weekly effect after Period 4** | 2019-04-28 to 2019-12-31 | 1.91 | 1.58 | 11.31 | 2.02 | 0.07 | -16.76 | -10.58 | 0.25 | -14.49 |
| **95% CI after Period 4** |  | [-3.39,7.25] | [-3.28,6.47] | [6.60,16.07] | [-2.52,6.59] | [-4.35,4.51] | [-19.82,-13.67] | [-13.56,-7.58] | [-2.80,3.34] | [-17.17,-11.78] |
| **Cluster names** |  | 10: Back and Spine disorders | 11: Occulomotor disorders | 12: Lower limb trauma | 13: Cutaneous infections, wounds, and skin disorders | 14: Arthropathies | 15: Shoulder and arm trauma | 16: Chest trauma and other diseases of the pleura | **Total** | **Total for negative trends (Clusters 2, 6, 7, 9, 11, 12 and 15)** |
| **Patient population count (2016 to 2019)** |  | 8,243 | 7,367 | 6,652 | 5,840 | 5,994 | 4,352 | 2,558 | 141,821 | 71,705 |
| **Weekly intercept** |  | 33.16 | 24.95 | 29.61 | 21.81 | 23.22 | 20.68 | 13.31 | 1052.45 | 366.14 |
| **Daily seasonal effect of month** | Jan. | 0.00 | 0.00 | 0.00 | 0.00 | 0.00 | 0.00 | 0.00 | 0.00 | 0.00 |
|  | Feb. | -0.10 | 0.34 | 0.24 | 0.36 | 0.53 | -0.11 | -0.08 | 1.03 | 0.31 |
|  | Mar. | -0.11 | 0.34 | 0.61 | 0.40 | 0.54 | 0.29 | 0.09 | 7.84 | 4.70 |
|  | Apr. | 0.01 | 0.92 | 0.88 | 0.79 | 0.67 | 0.24 | 0.18 | 6.88 | 6.12 |
|  | May. | 0.28 | 0.14 | 0.71 | 0.28 | 0.73 | 0.51 | 0.18 | 2.14 | 7.64 |
|  | Jun. | -0.32 | 0.76 | 1.01 | 0.59 | 0.59 | 0.59 | 0.16 | 6.44 | 8.29 |
|  | Jul. | 0.27 | 0.46 | 0.38 | 0.73 | 0.40 | 0.68 | 0.17 | 4.35 | 7.24 |
|  | Aug. | -0.26 | 0.70 | 0.10 | 0.82 | 0.57 | 0.40 | 0.24 | -5.02 | 3.09 |
|  | Sep. | 0.14 | 0.14 | 1.01 | 0.57 | 0.65 | 0.64 | 0.11 | 1.37 | 6.69 |
|  | Oct. | 0.31 | 0.45 | 0.81 | 0.55 | 0.64 | 0.46 | 0.20 | 5.52 | 7.58 |
|  | Nov. | 0.30 | 0.38 | 0.59 | 0.38 | 0.12 | 0.31 | -0.13 | 2.36 | 5.85 |
|  | Dec. | -0.12 | -0.05 | 0.21 | 0.19 | 0.10 | 0.20 | 0.04 | 11.77 | 1.60 |
| **Daily seasonal effect of day of the week** | Mon. | 0.00 | 0.00 | 0.00 | 0.00 | 0.00 | 0.00 | 0.00 | 0.00 | 0.00 |
|  | Tue. | 1.26 | 1.25 | -0.03 | 0.63 | 0.60 | -0.34 | 0.33 | 15.31 | 3.27 |
|  | Wed. | 0.60 | 0.85 | -0.58 | 0.48 | 0.18 | -0.53 | 0.09 | 3.73 | -0.31 |
|  | Thu. | 0.47 | 0.61 | -0.74 | 0.30 | 0.01 | -0.55 | 0.03 | 2.10 | -2.31 |
|  | Fri. | 0.25 | 0.48 | -0.39 | 0.11 | 0.11 | -0.39 | -0.09 | 4.03 | 0.61 |
|  | Sat. | 0.15 | 0.86 | -0.54 | 0.39 | 0.05 | -0.53 | -0.15 | 5.25 | -0.65 |
|  | Sun. | -0.12 | 1.07 | -0.45 | 0.23 | -0.31 | -0.23 | -0.08 | 2.08 | 0.35 |
| **Weekly effect before Period 1** | 01/01/2016 to 01/05/2017 | -2.42 | -1.74 | -1.86 | -2.58 | -3.36 | -0.45 | -1.31 | -87.82 | -18.60 |
| **95% CI before Period 1** |  | [-4.42,-0.41] | [-3.65,0.18] | [-3.72,-0.00] | [-4.35,-0.81] | [-5.09,-1.63] | [-2.05,1.16] | [-2.78,0.15] | [-98.87,-76.77] | [-25.30,-11.90] |
| **Weekly effect Before Period 2** | 01/05/2017 to 08/10/2018 | 0.00 | 0.00 | 0.00 | 0.00 | 0.00 | 0.00 | 0.00 | 0.00 | 0.00 |
| **Weekly effect After Period 3** | 08/10/2018 to 28/04/2019 | 1.91 | -3.41 | -2.51 | 0.99 | -0.45 | -1.70 | 1.06 | **-39.83** | **-39.17** |
| **95% CI after Period 3** |  | [-0.91,4.76] | [-5.94,-0.84] | [-5.01,0.03] | [-1.45,3.48] | [-2.81,1.96] | [-3.78,0.41] | [-0.93,3.10] | **[-55.13,-24.49]** | **[-48.06,-30.25]** |
| **Weekly effect after Period 4** | 28/04/2019 to 31/12/2019 | -0.34 | -3.70 | -6.57 | 1.60 | -3.40 | -2.08 | -2.00 | -44.79 | -54.12 |
| **95% CI after Period 4** |  | [-2.77,2.12] | [-5.94,-1.43] | [-8.69,-4.43] | [-0.57,3.81] | [-5.45,-1.32] | [-3.99,-0.14] | [-3.68,-0.26] | [-58.12,-31.42] | [-61.95,-46.25] |

*Except Cluster names and the patient population count rows, all rows indicate that the regression coefficient of the model regressed on the corresponding cluster (or patient population). These coefficients express a variation in emergency department (ED) visits per day (noted as daily effect) or in ED visits per week (noted as weekly effect).
