# Supplementary material for: Multimorbidity clustering of the emergency department patient flow: Impact analysis of new unscheduled care clinics
Source: PLoS One. 2022 Jan 31;17(1):e0262914. doi: 10.1371/journal.pone.0262914 (PMC8803184; doi:10.1371/journal.pone.0262914)
Supplement: S4 Table — (DOCX) [file pone.0262914.s004.docx]

**S5 Table.** Coefficient result Poisson regression model with global trend of emergency department (ED) visits counts per day from 2016 to 2019

| **Cluster names** |  | 1: Digestive disorders, pregnancy, menstruation | 2: General symptoms and mental disorders | 3: Infectious diseases | 4: General symptoms of chronic conditions | 5: Mental disorders and at-risk behaviors | 6: Wrist and Hand Trauma | 7: Head Trauma | 8: Hip related trauma and disorders | 9: Feet Trauma |
| --- | --- | --- | --- | --- | --- | --- | --- | --- | --- | --- |
| **Patient population count (2016 to 2019)** |  | 30,166 | 29,388 | 24,512 | 25,893 | 22,100 | 13,990 | 13,595 | 11,955 | 10,514 |
| **Weekly intercept** |  | 123.82 | 120.90 | 138.32 | 129.30 | 98.70 | 51.31 | 62.75 | 47.60 | 44.28 |
| **annual trend (weekly effect increase)** |  | 13.08 | 0.79 | 10.78 | 5.96 | 1.98 | 5.22 | 1.42 | 1.54 | 0.83 |
| **95% CI annual trend** |  | [7.84,18.33] | [-4.16,5.74] | [6.00,15.57] | [1.32,10.61] | [-2.50,6.47] | [1.91,8.52] | [-1.73,4.57] | [-1.53,4.61] | [-2.07,3.72] |
| **Daily seasonal effect of month** | Jan. | 0.00 | 0.00 | 0.00 | 0.00 | 0.00 | 0.00 | 0.00 | 0.00 | 0.00 |
|  | Feb. | 1.81 | 0.96 | -1.54 | -2.32 | 0.74 | -0.63 | 0.01 | 0.60 | -0.24 |
|  | Mar. | 2.30 | 0.91 | -1.71 | -2.12 | 2.19 | 0.57 | 0.33 | 0.50 | 1.12 |
|  | Apr. | 1.12 | -0.10 | -2.14 | -4.56 | 2.28 | 1.15 | 0.99 | 1.03 | 1.46 |
|  | May. | 2.09 | 1.45 | -7.01 | -4.97 | 3.04 | 1.45 | 1.06 | 1.01 | 2.34 |
|  | Jun. | 1.22 | 1.41 | -3.87 | -5.43 | 4.69 | 1.45 | 0.99 | 0.87 | 1.90 |
|  | Jul. | 0.76 | 2.03 | -4.33 | -6.24 | 4.83 | 1.07 | 1.30 | 0.70 | 1.45 |
|  | Aug. | 0.84 | 1.34 | -7.52 | -7.08 | 2.83 | -0.35 | -0.32 | 1.03 | 0.94 |
|  | Sep. | 0.61 | 1.02 | -5.57 | -5.91 | 2.55 | 1.02 | 0.90 | 0.75 | 1.63 |
|  | Oct. | 0.77 | 1.79 | -2.65 | -4.74 | 1.58 | 1.00 | 1.09 | 1.01 | 1.64 |
|  | Nov. | 0.56 | 1.41 | -2.74 | -3.97 | 1.20 | 0.61 | 1.04 | 0.40 | 1.10 |
|  | Dec. | 1.39 | 1.12 | 6.82 | -1.01 | 1.23 | -0.29 | 0.63 | 1.00 | -0.45 |
| **Daily seasonal effect of day of the week** | Mon. | 0.00 | 0.00 | 0.00 | 0.00 | 0.00 | 0.00 | 0.00 | 0.00 | 0.00 |
|  | Tue. | 4.58 | 2.79 | 0.69 | 4.86 | -0.96 | 0.73 | -1.62 | 0.23 | 0.34 |
|  | Wed. | 1.81 | 2.04 | -0.98 | 2.80 | -1.13 | -0.17 | -1.76 | -0.06 | -0.28 |
|  | Thu. | 2.15 | 1.21 | -0.47 | 2.74 | -0.85 | -0.05 | -2.46 | -0.15 | -0.37 |
|  | Fri. | 2.18 | 1.87 | -1.18 | 3.07 | -0.31 | 0.25 | -1.45 | -0.74 | 0.03 |
|  | Sat. | 1.63 | 1.12 | -0.72 | 3.77 | 0.15 | 0.16 | -1.35 | 0.68 | -0.49 |
|  | Sun. | 0.63 | 1.06 | -0.61 | 1.70 | 0.01 | 0.24 | -1.07 | 0.28 | -0.35 |
| **Weekly effect before Period 1** | 2016-01-01 to 2017-05-01 | -2.46 | 1.85 | -6.25 | -4.86 | 5.74 | 1.58 | -1.14 | -1.75 | -4.15 |
| **95% CI before Period 1** |  | [-11.06,6.13] | [-6.22,9.92] | [-13.76,1.25] | [-12.45,2.71] | [-1.60,13.07] | [-3.83,6.99] | [-6.31,4.03] | [-6.77,3.27] | [-8.89,0.59] |
| **Weekly effect before Period 2** | 2017-05-01 to 2018-10-08 | 0.00 | 0.00 | 0.00 | 0.00 | 0.00 | 0.00 | 0.00 | 0.00 | 0.00 |
| **Weekly effect after Period 3** | 2018-10-08 to 2019-04-28 | -17.05 | -9.12 | -14.05 | -2.14 | 1.84 | -13.10 | -7.50 | -3.39 | -8.75 |
| **95% CI after Period 3** |  | [-24.78,-9.31] | [-16.14,-2.10] | [-21.19,-6.90] | [-9.10,4.83] | [-4.57,8.25] | [-17.80,-8.40] | [-12.02,-2.98] | [-7.82,1.04] | [-12.80,-4.69] |
| **Weekly effect after Period 4** | 2019-04-28 to 2019-12-31 | -19.32 | 0.29 | -6.57 | -7.59 | -3.18 | -25.27 | -12.89 | -2.25 | -15.85 |
| **95% CI after Period 4** |  | [-29.41,-9.25] | [-9.19,9.75] | [-15.88,2.71] | [-16.40,1.18] | [-11.76,5.39] | [-31.51,-19.05] | [-18.84,-6.97] | [-8.11,3.60] | [-21.33,-10.39] |
| **Cluster names** |  | 10: Back and spine disorders | 11: Occulomotor disorders | 12: Lower limb trauma | 13: Cutaneous infections, wounds and skin disorders | 14: Arthropathies | 15: Shoulder and arm trauma | 16: Chest trauma and other diseases of the pleura | **Total** | **Total for negative trends (Clusters 2, 6, 7, 9, 11, 12 and 15)** |
| **Patient population count (2016 to 2019)** |  | 8,243 | 7,367 | 6,652 | 5,840 | 5,994 | 4,352 | 2,558 | 141,821 | 71,705 |
| **Weekly intercept** |  | 28.37 | 24.85 | 29.61 | 18.37 | 22.92 | 21.06 | 13.88 | 951.45 | 344.31 |
| **Annual Trend (weekly effect increase)** |  | 2.37 | 0.05 | 0.00 | 1.75 | 0.16 | -0.19 | -0.29 | 49.76 | 10.76 |
| **95% CI Annual Trend** |  | [-0.08,4.81] | [-2.33,2.44] | [-2.31,2.31] | [-0.42,3.91] | [-1.99,2.30] | [-2.12,1.74] | [-2.04,1.47] | [36.15,63.36] | [2.55,18.97] |
| **Daily seasonal effect of month** | Jan. | 0.00 | 0.00 | 0.00 | 0.00 | 0.00 | 0.00 | 0.00 | 0.00 | 0.00 |
|  | Feb. | -0.14 | 0.34 | 0.24 | 0.32 | 0.53 | -0.10 | -0.08 | 0.45 | 0.18 |
|  | Mar. | -0.17 | 0.34 | 0.61 | 0.34 | 0.53 | 0.30 | 0.10 | 6.71 | 4.43 |
|  | Apr. | -0.08 | 0.92 | 0.88 | 0.73 | 0.67 | 0.24 | 0.19 | 5.20 | 5.75 |
|  | May. | 0.34 | 0.14 | 0.71 | 0.34 | 0.73 | 0.51 | 0.17 | 3.74 | 7.98 |
|  | Jun. | -0.28 | 0.76 | 1.01 | 0.61 | 0.59 | 0.59 | 0.15 | 7.42 | 8.51 |
|  | Jul. | 0.28 | 0.46 | 0.38 | 0.73 | 0.40 | 0.68 | 0.17 | 4.73 | 7.32 |
|  | Aug. | -0.29 | 0.70 | 0.10 | 0.80 | 0.56 | 0.41 | 0.24 | -5.25 | 3.04 |
|  | Sep. | 0.08 | 0.14 | 1.01 | 0.54 | 0.64 | 0.65 | 0.12 | 0.53 | 6.51 |
|  | Oct. | 0.29 | 0.45 | 0.81 | 0.53 | 0.63 | 0.46 | 0.20 | 5.30 | 7.53 |
|  | Nov. | 0.26 | 0.38 | 0.59 | 0.35 | 0.12 | 0.31 | -0.12 | 1.87 | 5.74 |
|  | Dec. | -0.18 | -0.05 | 0.21 | 0.14 | 0.09 | 0.21 | 0.05 | 10.72 | 1.37 |
| **Daily seasonal effect of day of the week** | Mon. | 0.00 | 0.00 | 0.00 | 0.00 | 0.00 | 0.00 | 0.00 | 0.00 | 0.00 |
|  | Tue. | 1.27 | 1.25 | -0.03 | 0.63 | 0.60 | -0.34 | 0.33 | 15.35 | 3.28 |
|  | Wed. | 0.60 | 0.85 | -0.58 | 0.47 | 0.18 | -0.53 | 0.09 | 3.73 | -0.30 |
|  | Thu. | 0.47 | 0.61 | -0.74 | 0.29 | 0.01 | -0.55 | 0.03 | 2.10 | -2.31 |
|  | Fri. | 0.26 | 0.48 | -0.39 | 0.12 | 0.11 | -0.39 | -0.09 | 4.03 | 0.61 |
|  | Sat. | 0.17 | 0.86 | -0.54 | 0.39 | 0.05 | -0.53 | -0.15 | 5.27 | -0.65 |
|  | Sun. | -0.11 | 1.07 | -0.45 | 0.22 | -0.31 | -0.23 | -0.08 | 2.10 | 0.36 |
| **Weekly effect before Period 1** | 01/01/2016 to 01/05/2017 | 0.96 | -1.67 | -1.86 | -0.16 | -3.13 | -0.72 | -1.71 | -17.62 | -3.40 |
| **95% CI before Period 1** |  | [-3.06,4.98] | [-5.55,2.21] | [-5.56,1.84] | [-3.64,3.33] | [-6.66,0.38] | [-3.91,2.47] | [-4.56,1.13] | [-39.75,4.50] | [-16.79,9.99] |
| **Weekly effect before Period 2** | 01/05/2017 to 08/10/2018 | 0.00 | 0.00 | 0.00 | 0.00 | 0.00 | 0.00 | 0.00 | 0.00 | 0.00 |
| **Weekly effect after Period 3** | 08/10/2018 to 28/04/2019 | -0.26 | -3.45 | -2.51 | -0.63 | -0.59 | -1.53 | 1.32 | **-85.37** | **-48.99** |
| **95% CI after Period 3** |  | [-3.88,3.37] | [-6.79,-0.11] | [-5.79,0.78] | [-3.81,2.57] | [-3.67,2.50] | [-4.26,1.21] | [-1.25,3.91] | **[-105.16,-65.58]** | **[-60.63,-37.33]** |
| **Weekly effect after Period 4** | 28/04/2019 to 31/12/2019 | -4.18 | -3.79 | -6.58 | -1.28 | -3.66 | -1.77 | -1.53 | -125.99 | -71.74 |
| **95% CI after Period 4** |  | [-8.86,0.48] | [-8.31,0.71] | [-10.97,-2.22] | [-5.49,2.91] | [-7.72,0.38] | [-5.47,1.91] | [-4.88,1.80] | [-151.98,-100.03] | [-87.34,-56.16] |

*Except Cluster names and the patient population count rows, all rows indicate that the regression coefficient of the model regressed on the corresponding cluster (or patient population), these coefficients express a variation in emergency department (ED) visits per day (noted as daily effect) or in ED visits per week (noted as weekly effect).
