# Supplementary material for: Multimorbidity clustering of the emergency department patient flow: Impact analysis of new unscheduled care clinics
Source: PLoS One. 2022 Jan 31;17(1):e0262914. doi: 10.1371/journal.pone.0262914 (PMC8803184; doi:10.1371/journal.pone.0262914)
Supplement: S1 Fig — (DOCX) [file pone.0262914.s005.docx]

| **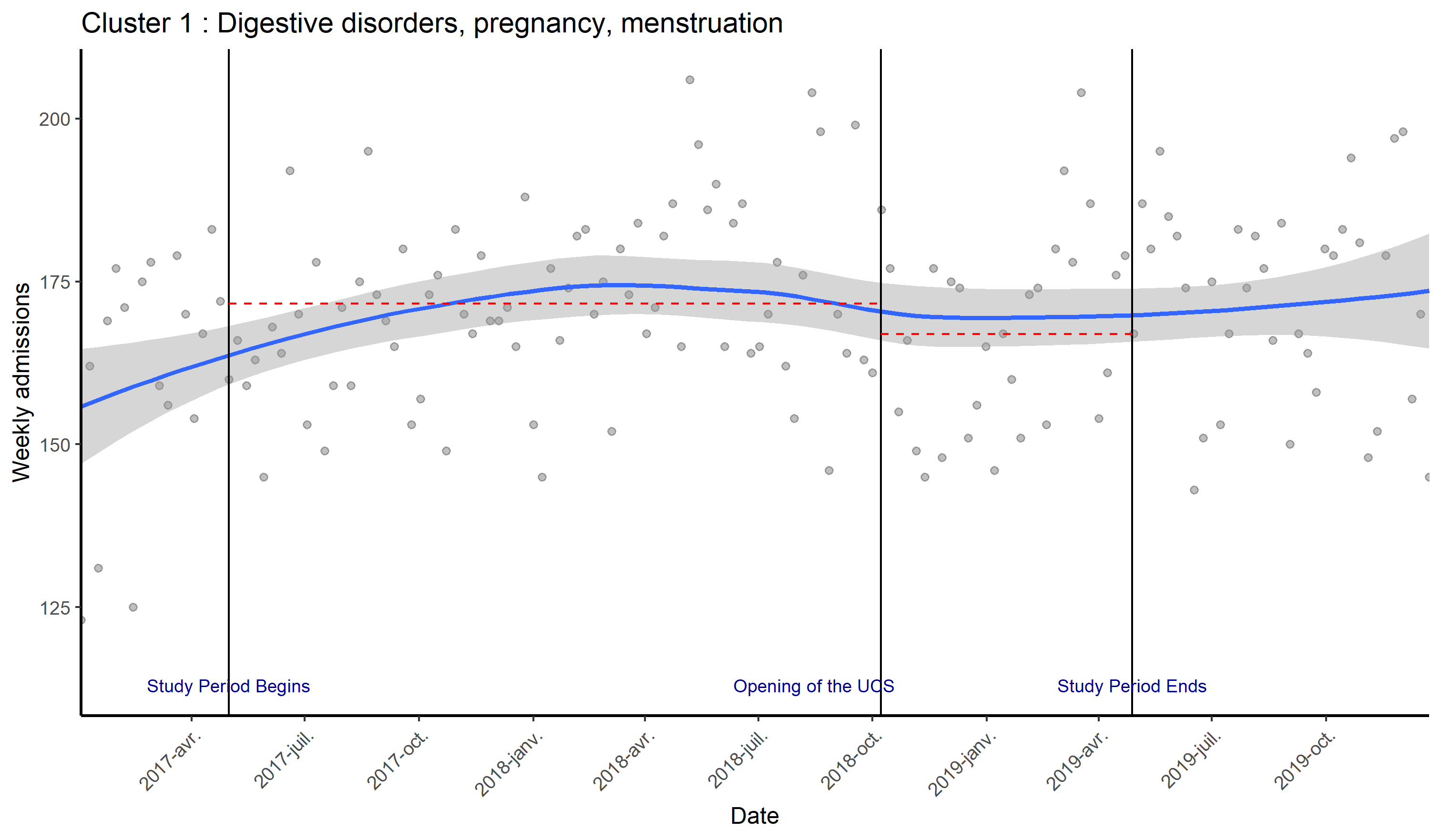** | 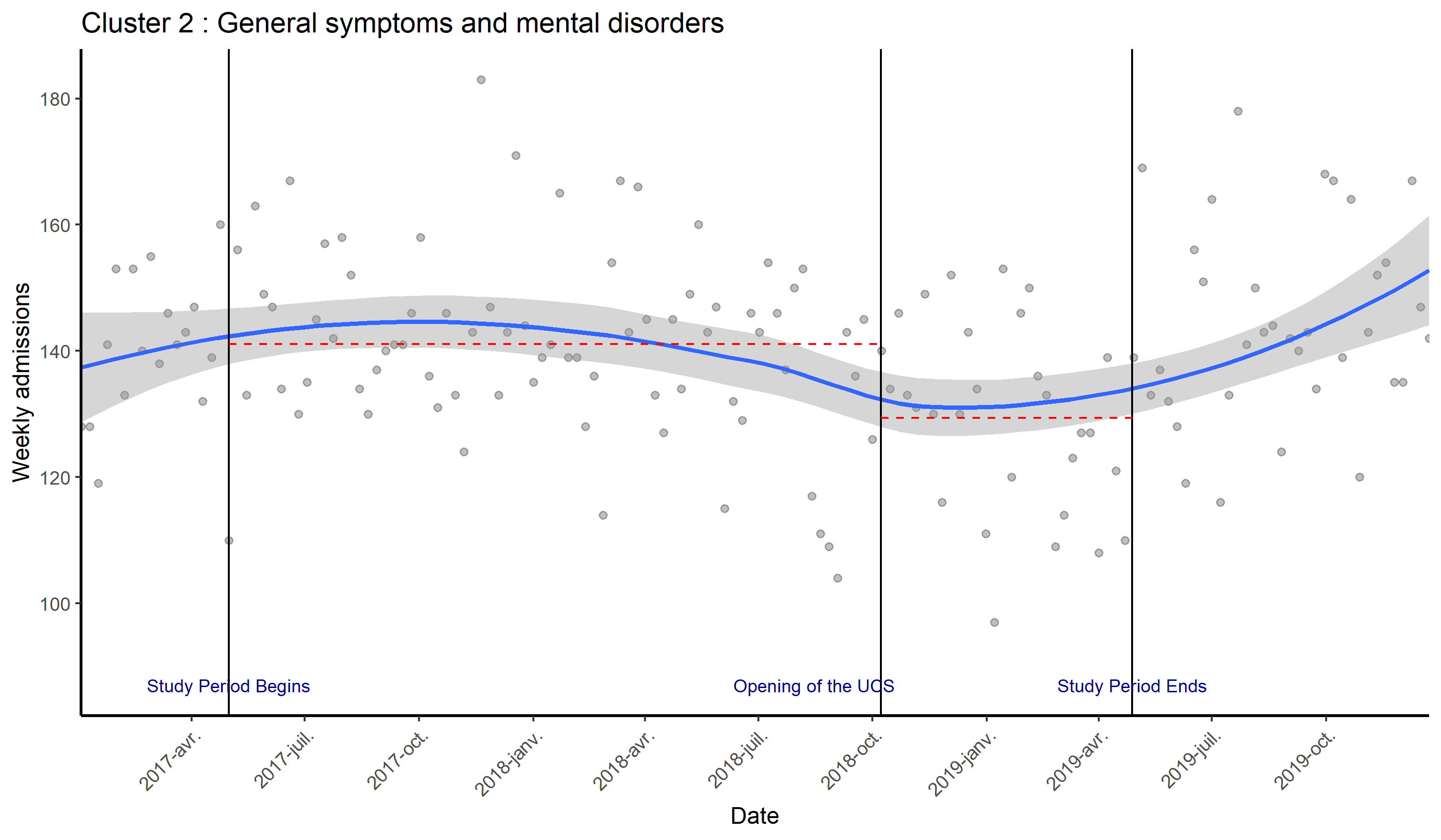 |
| --- | --- |
| **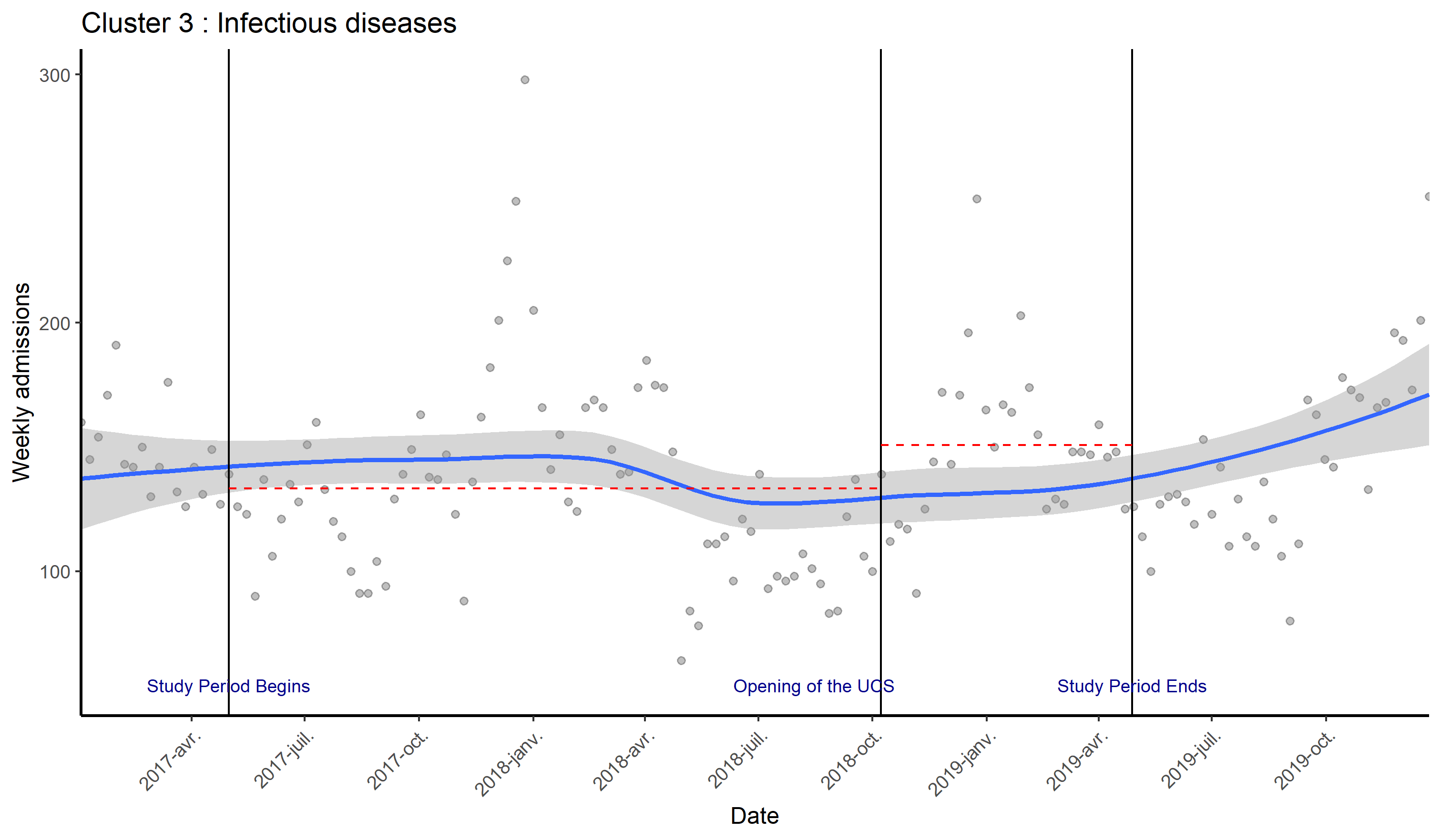** | **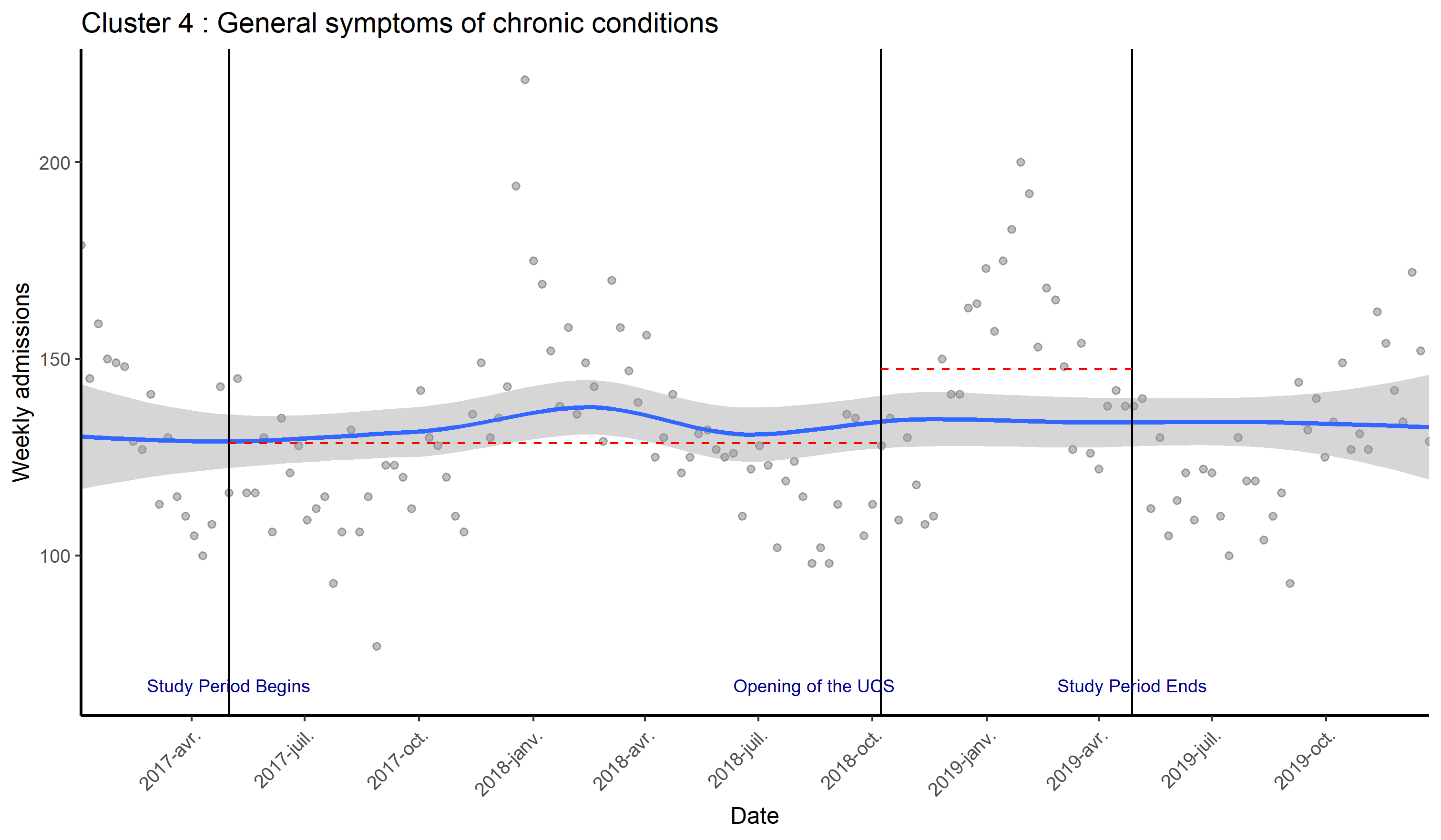** |
| **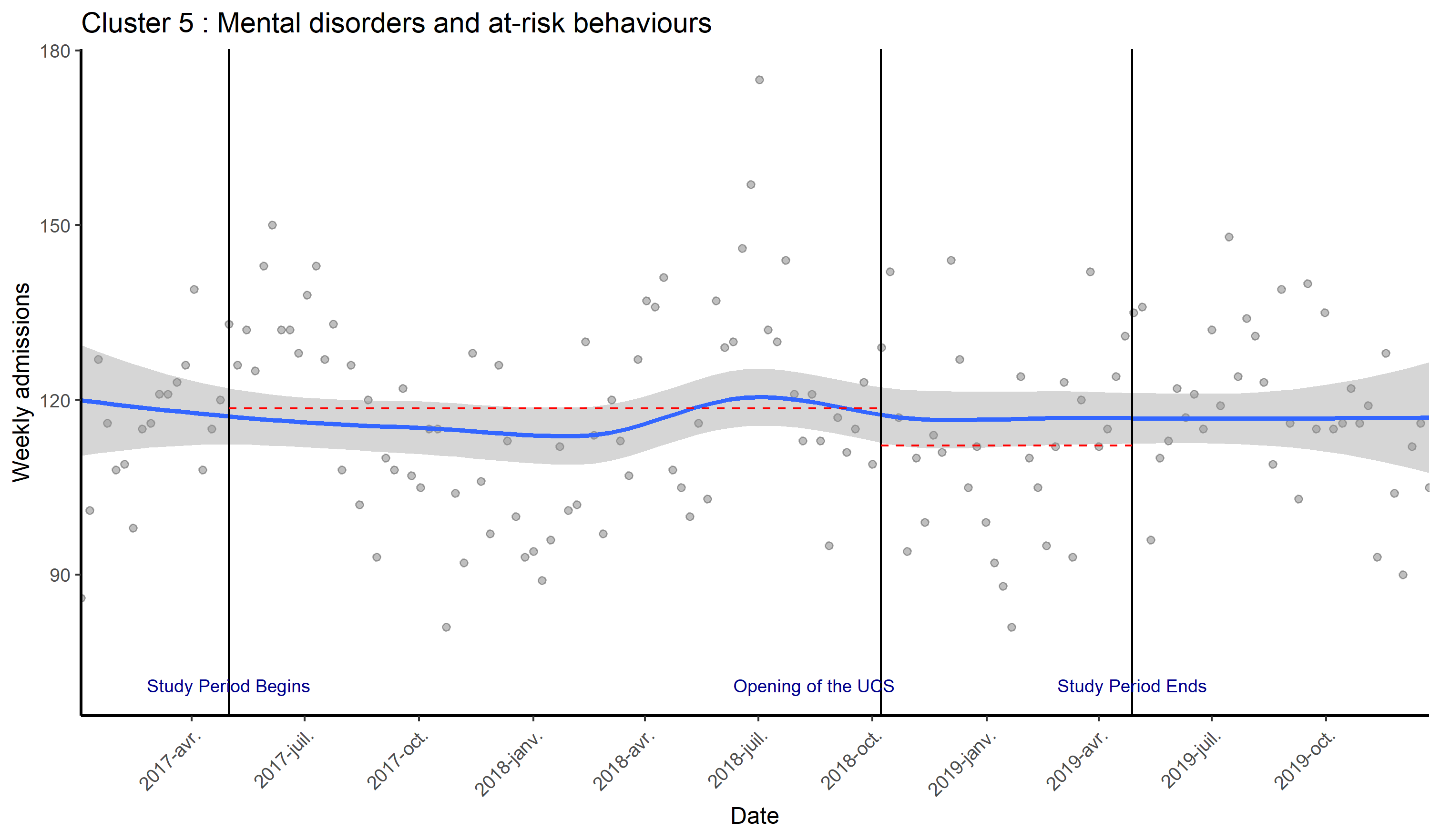** | **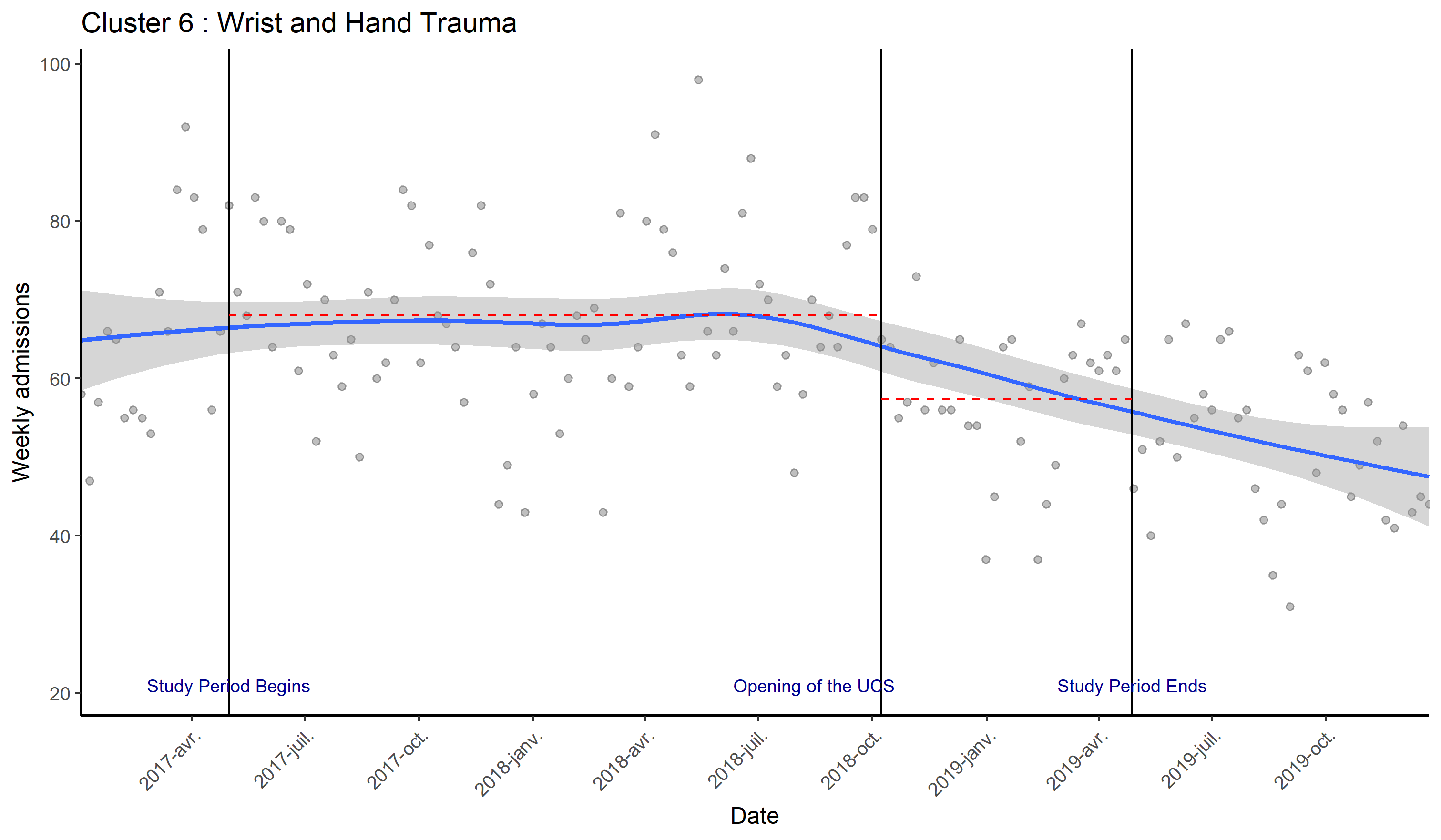** |
| **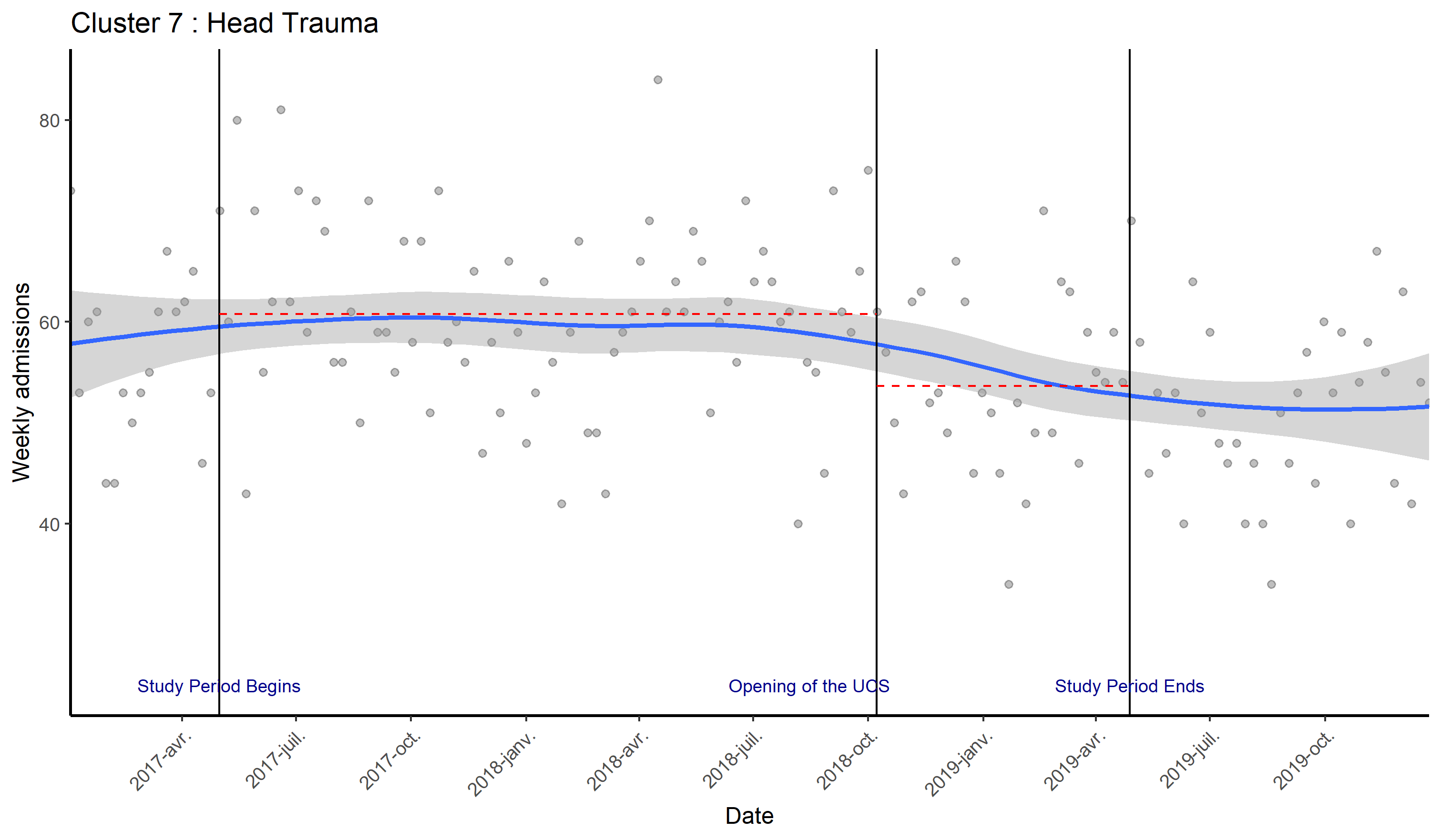** | **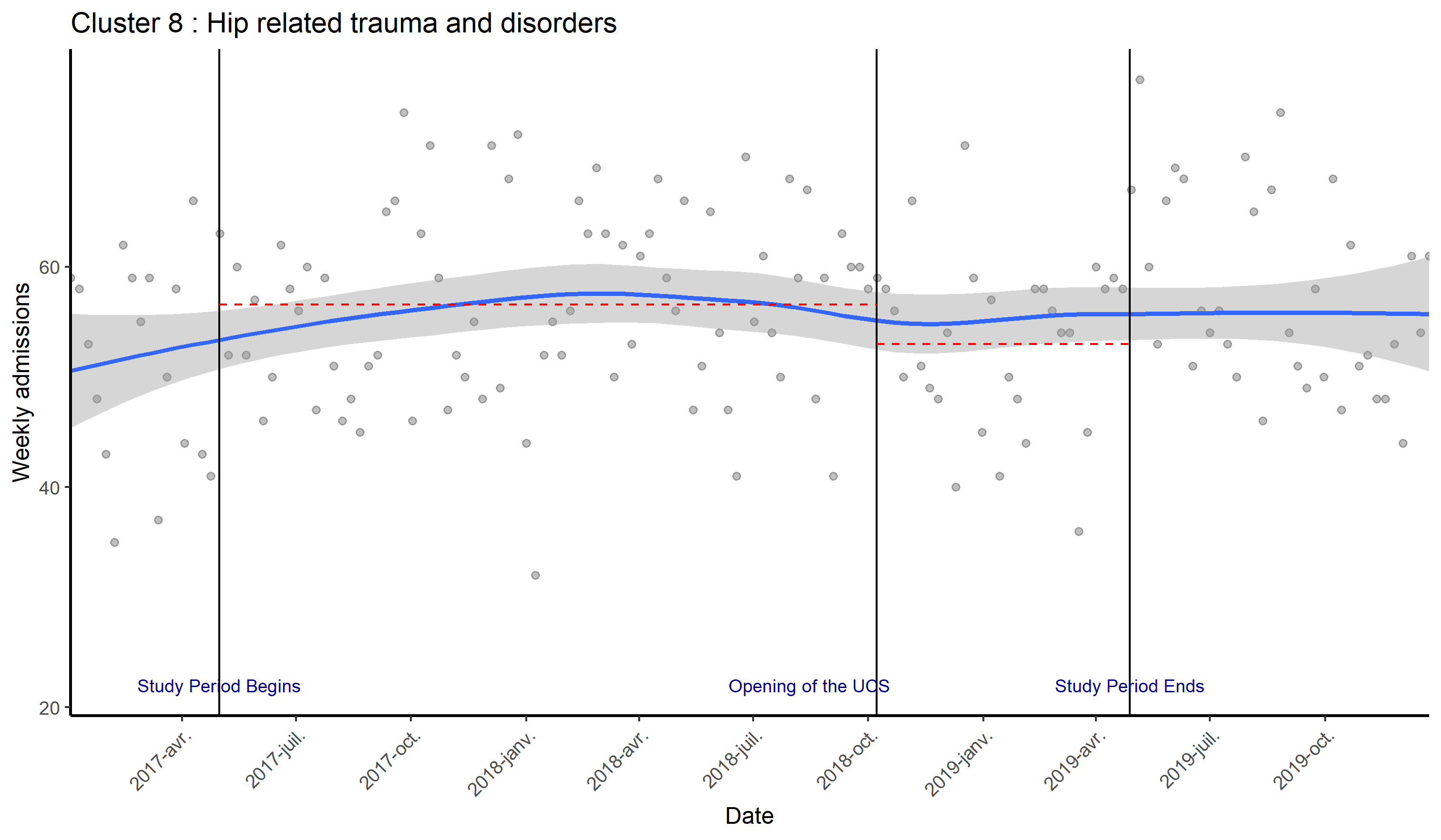** |
| **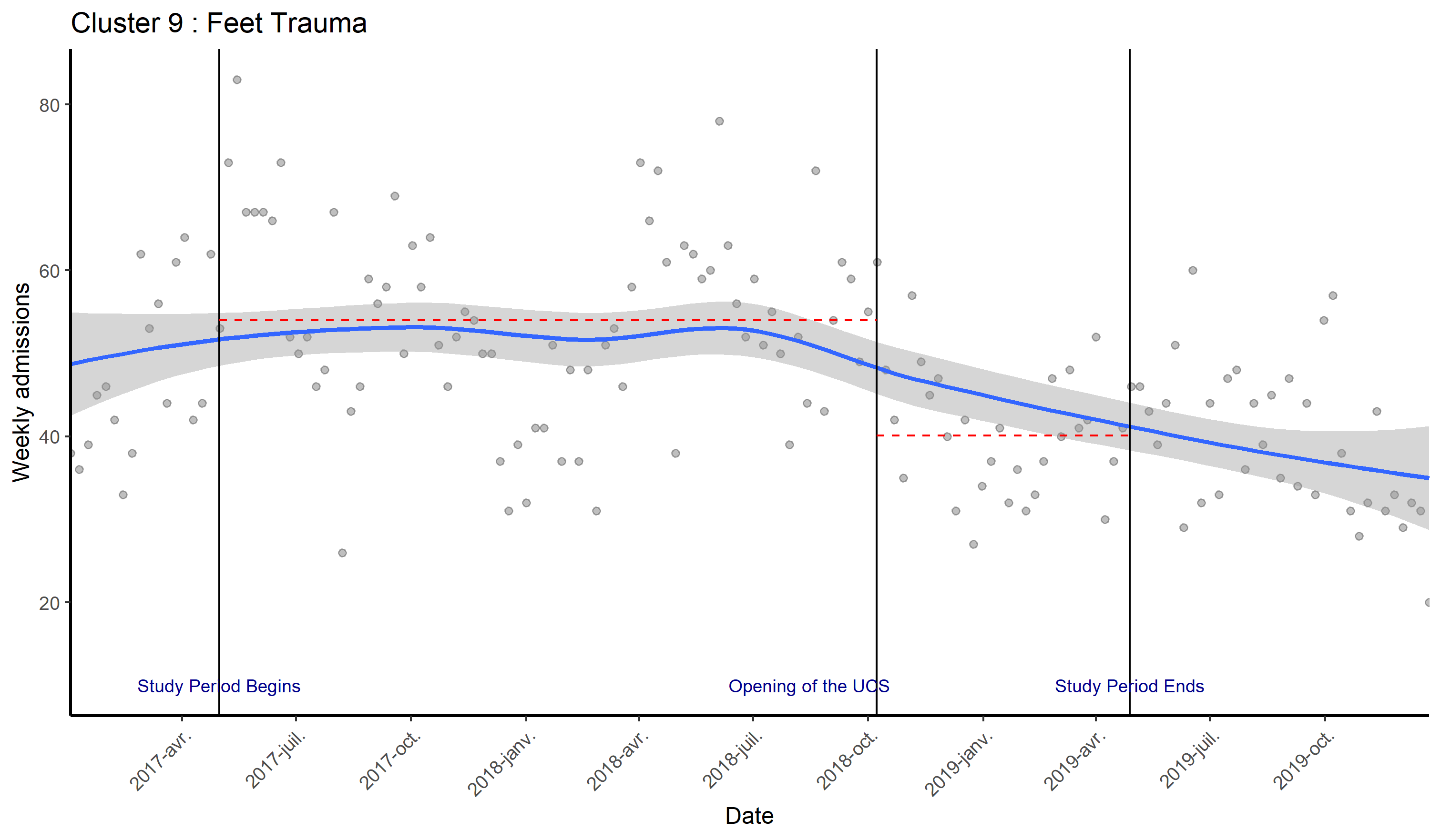** | **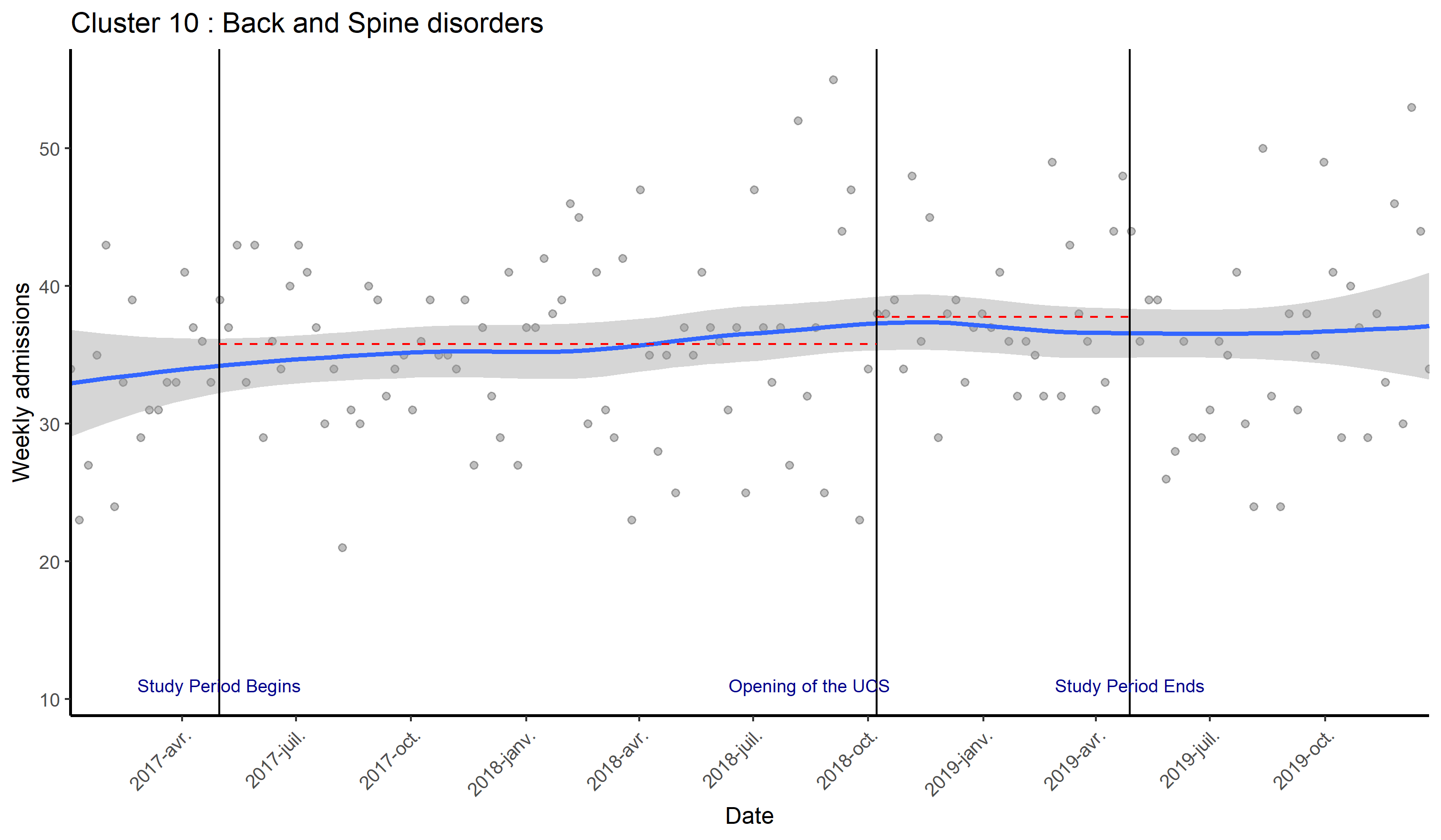** |
| **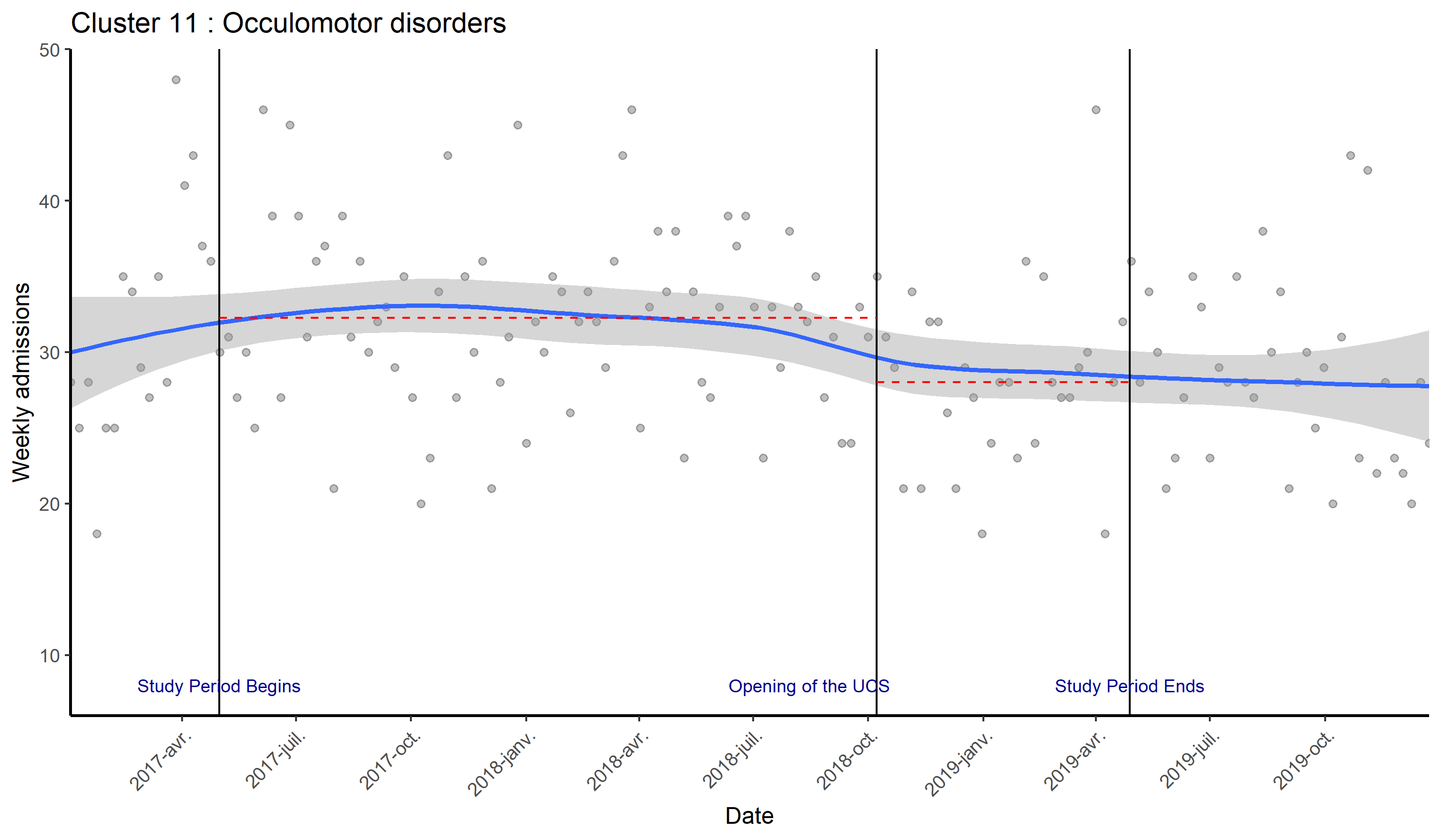** | **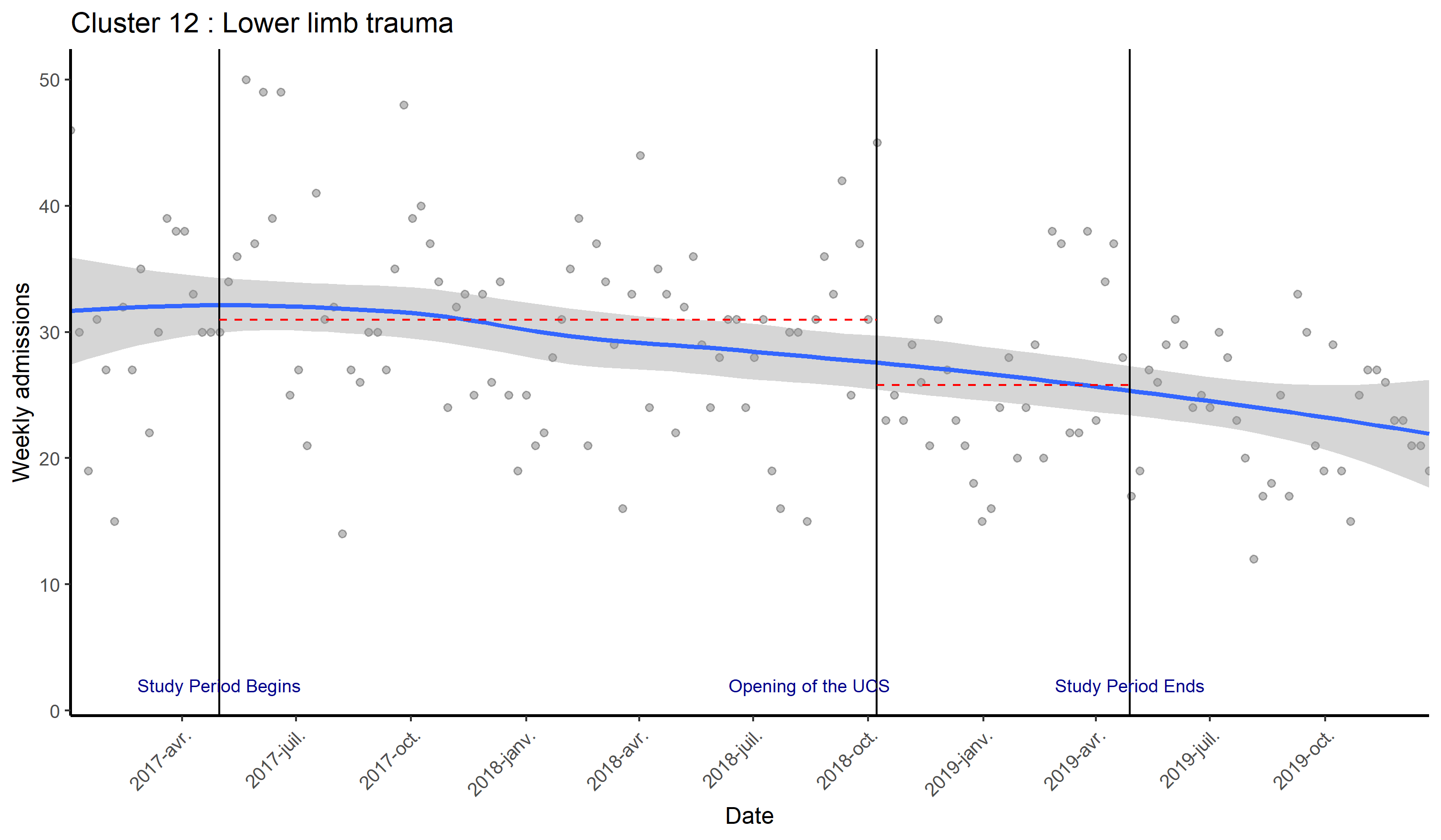** |
| **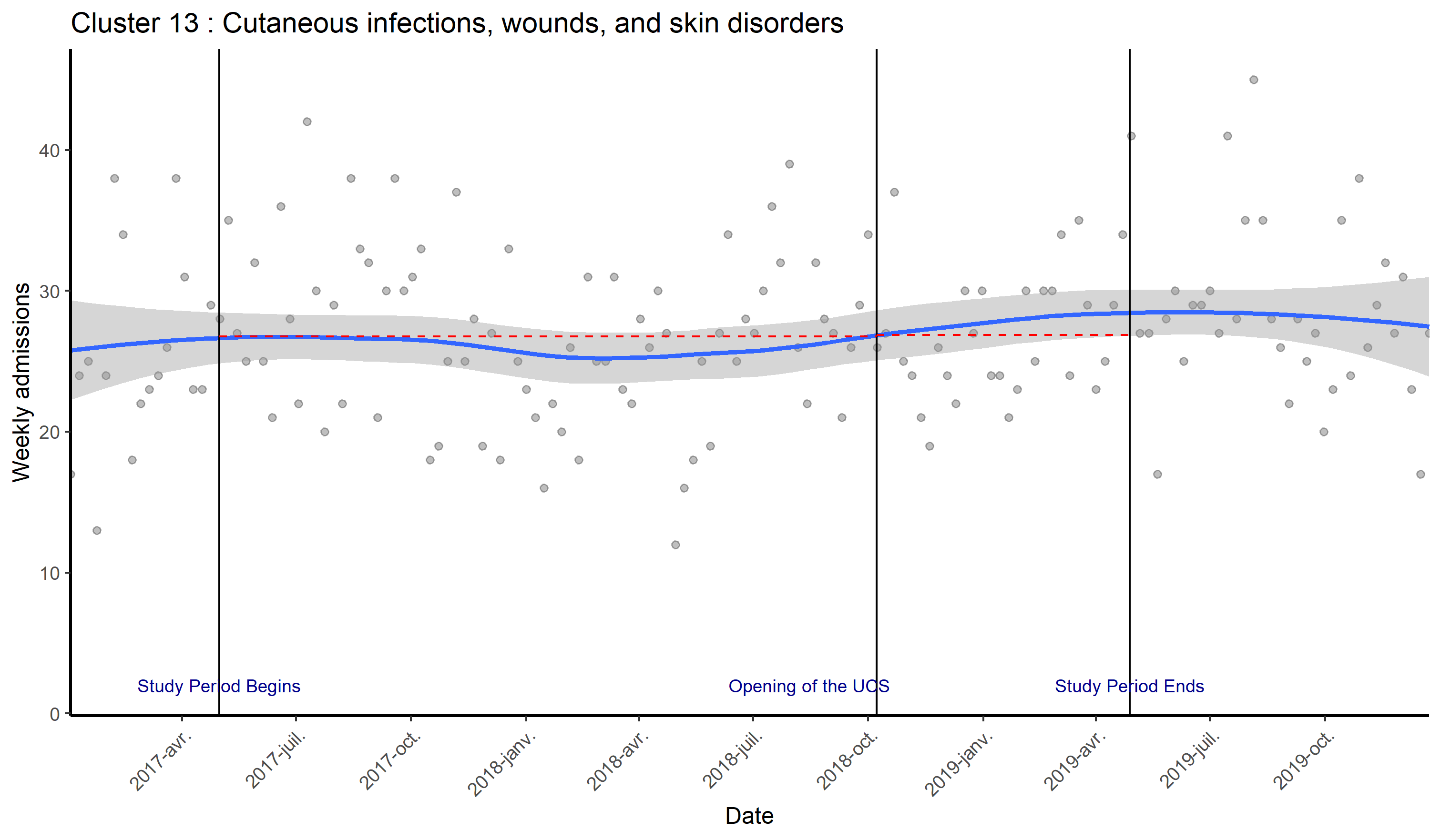** | **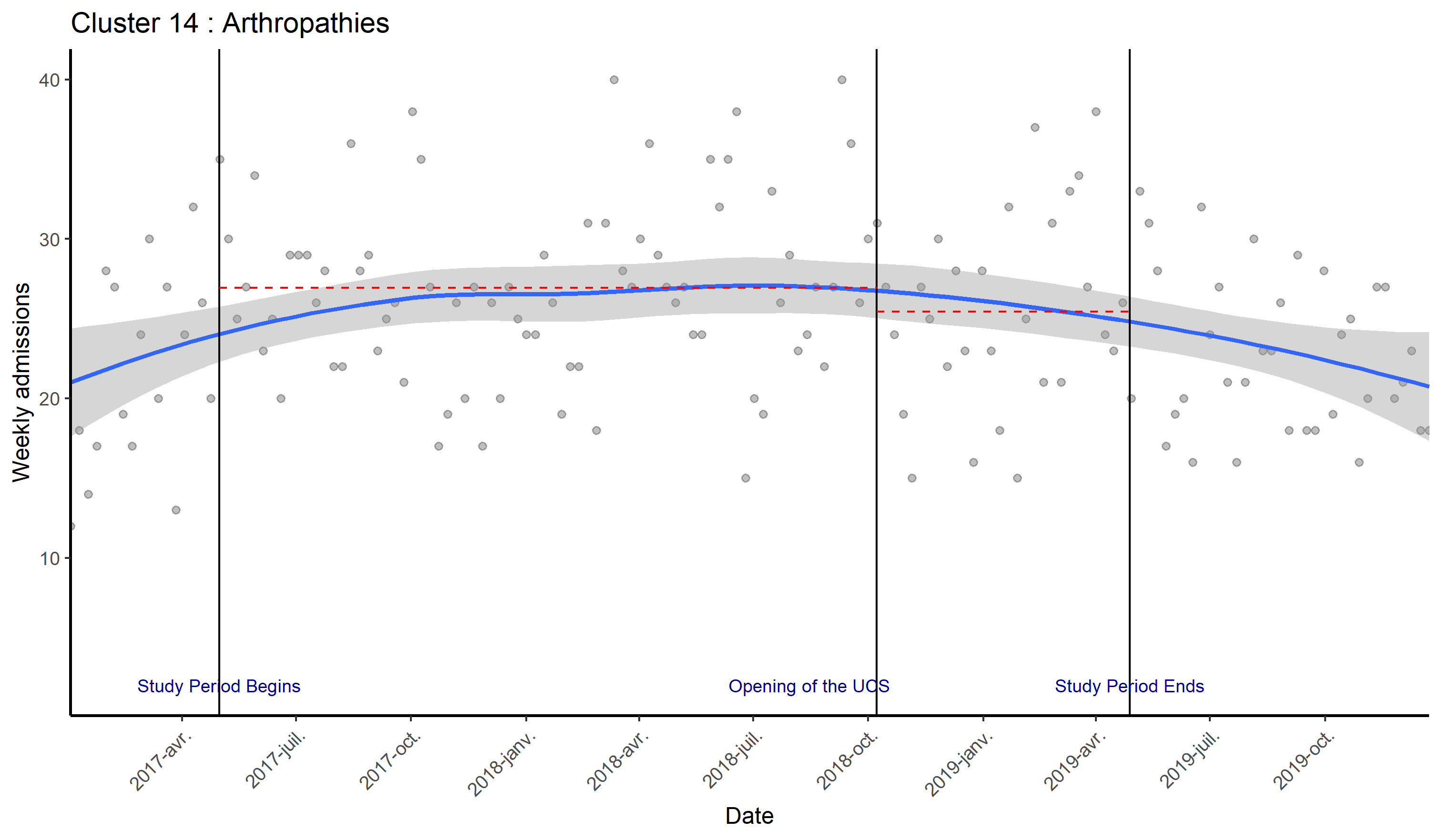** |
| **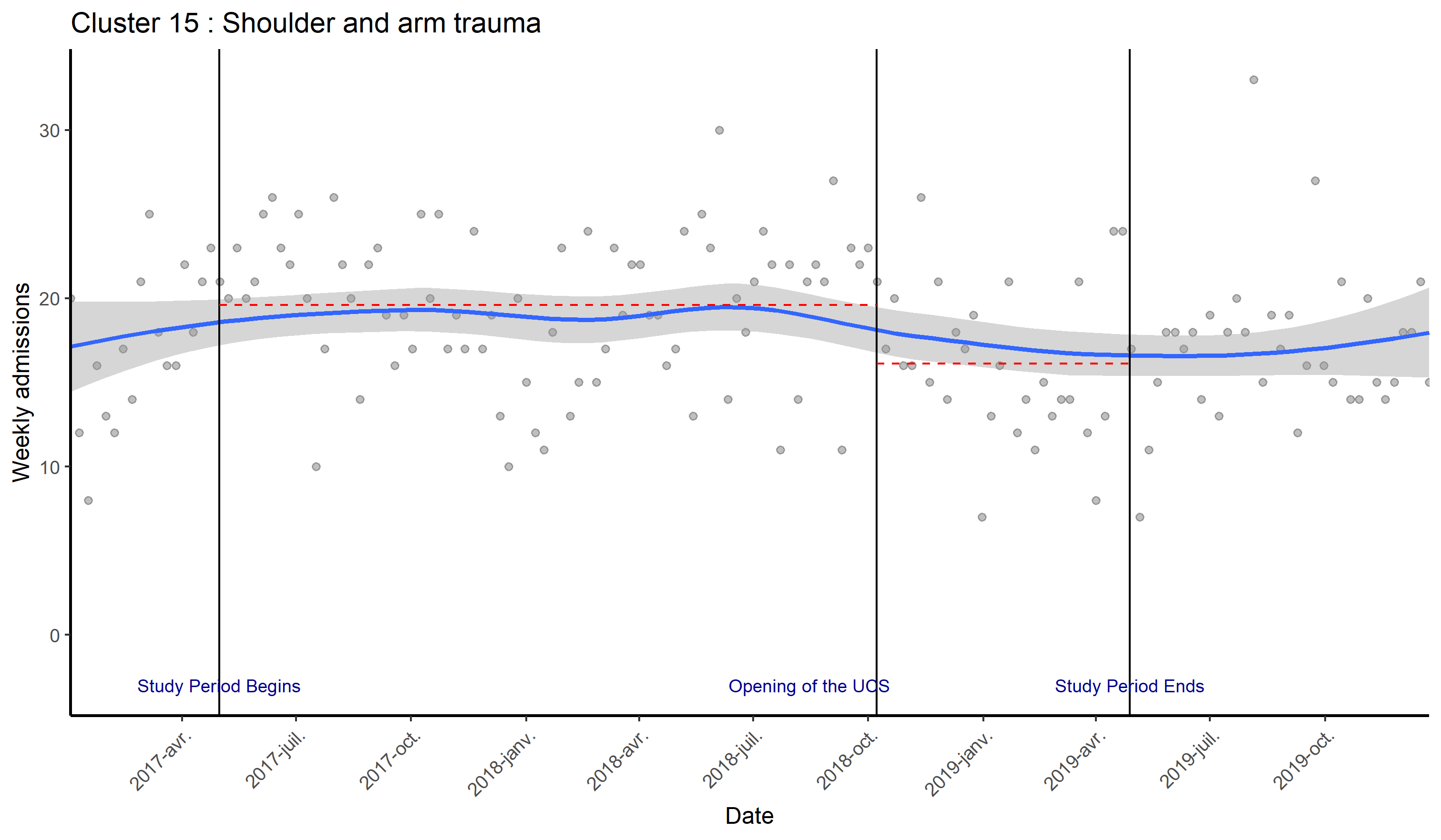** | **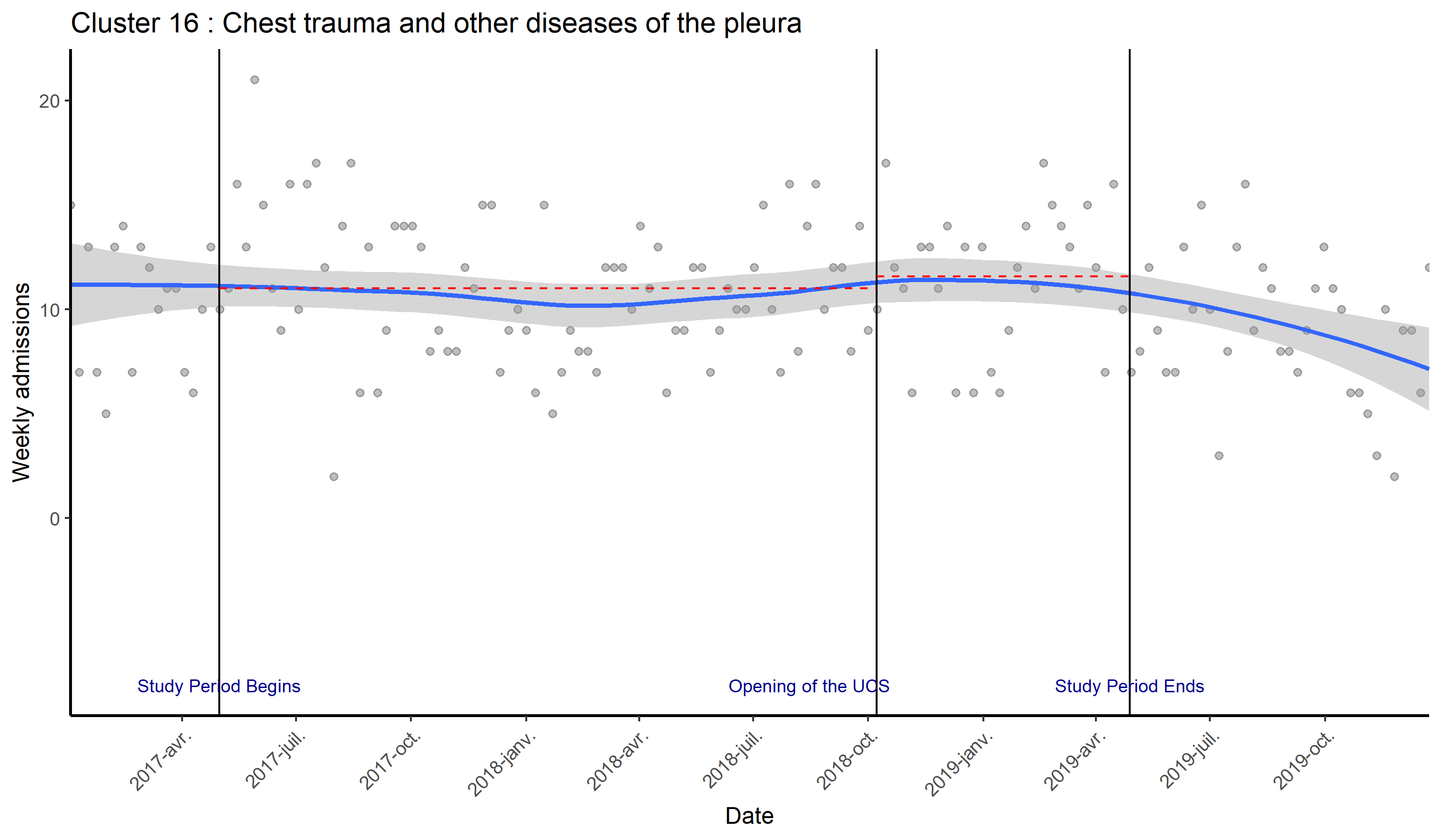** |

**With the 21 ICD 10 chapters**

| **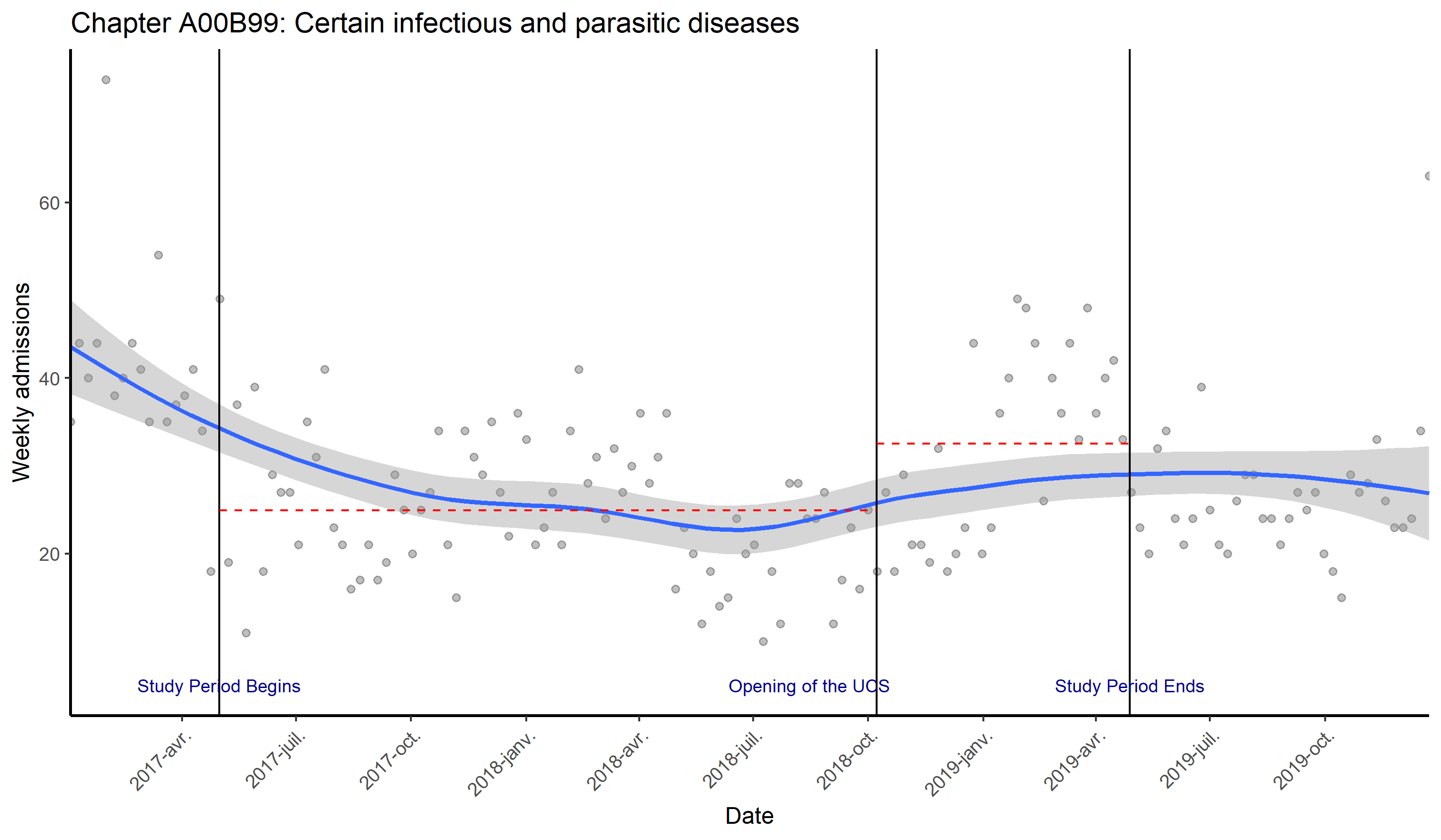** | **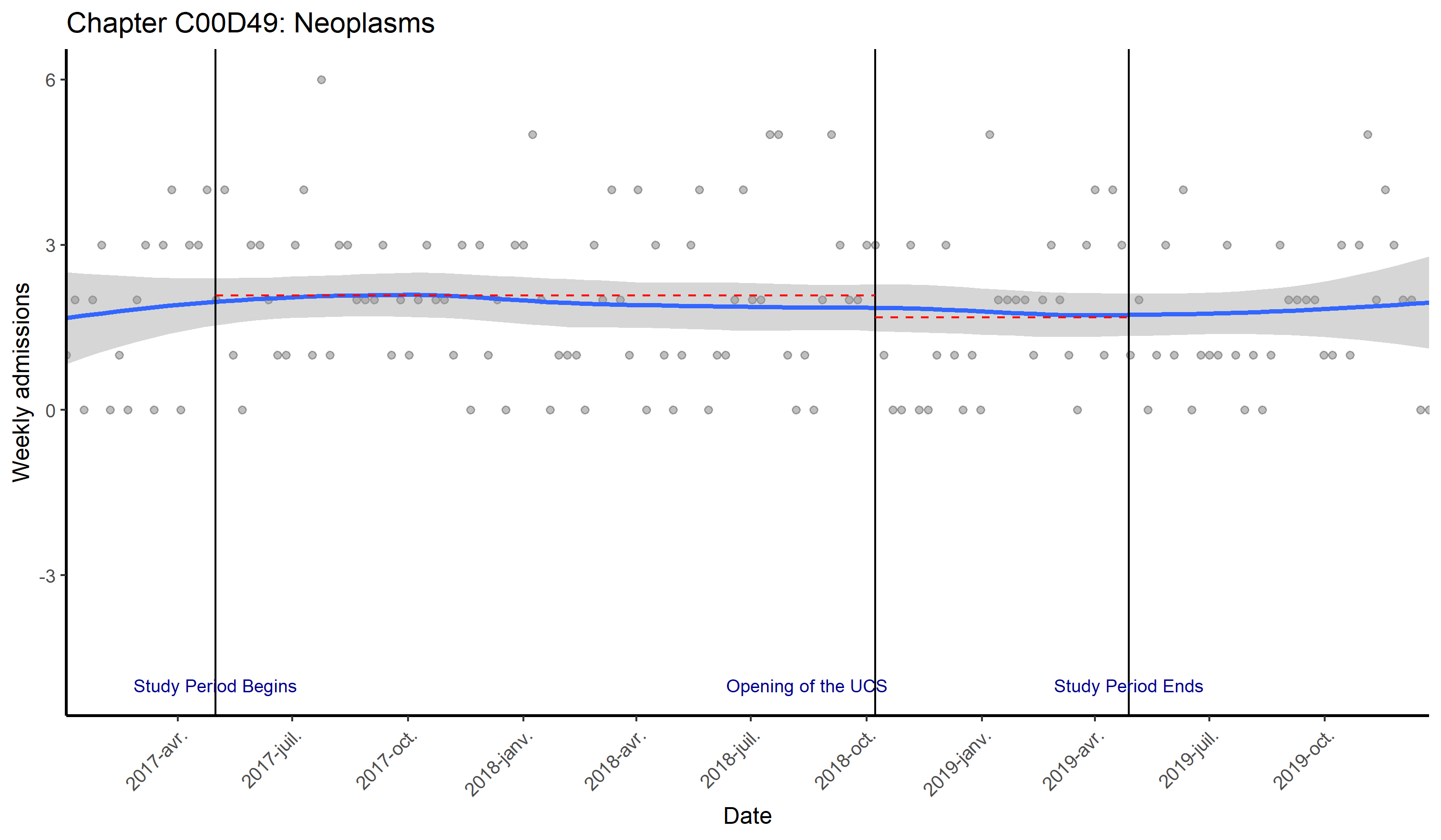** |
| --- | --- |
| **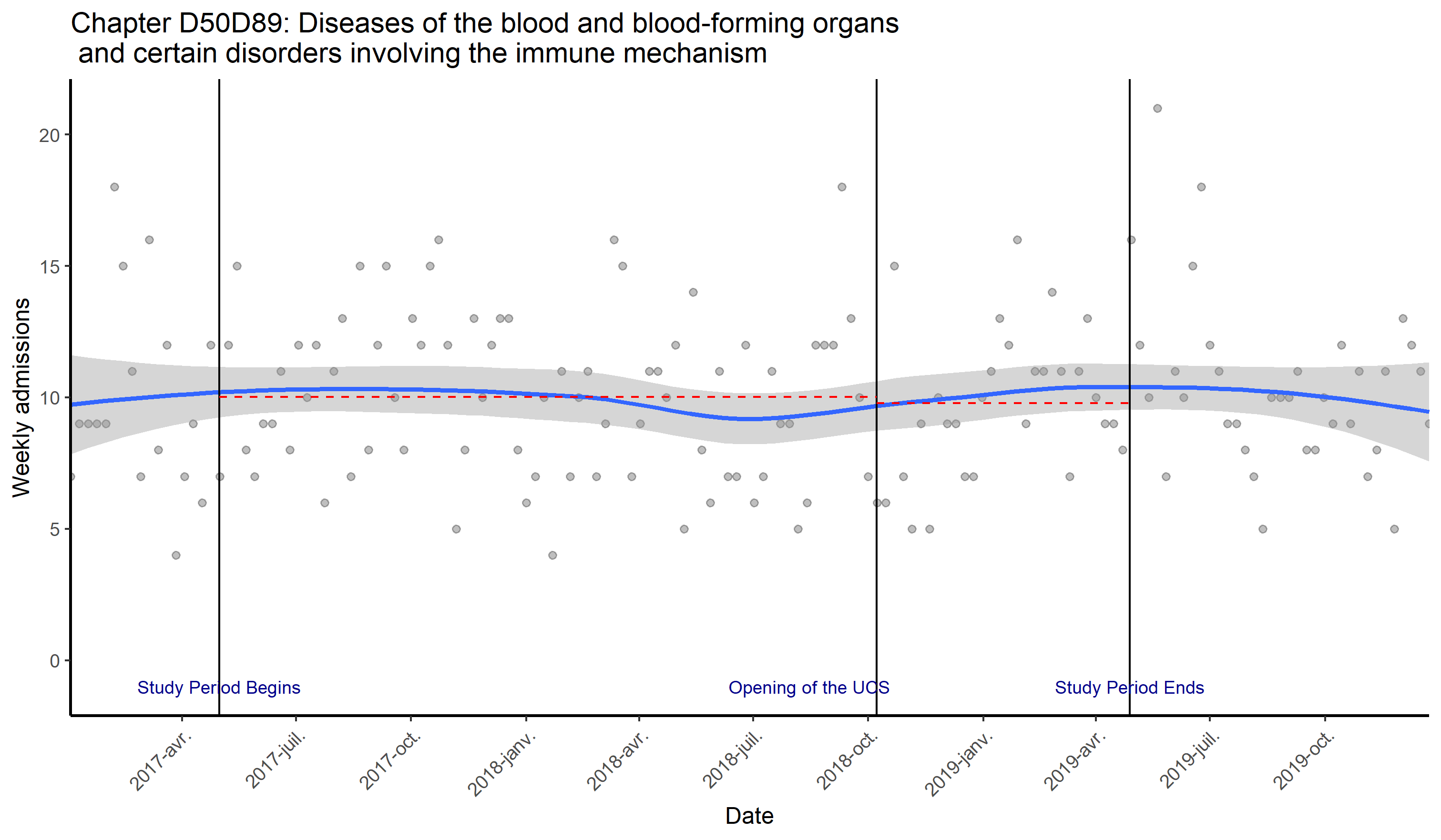** | **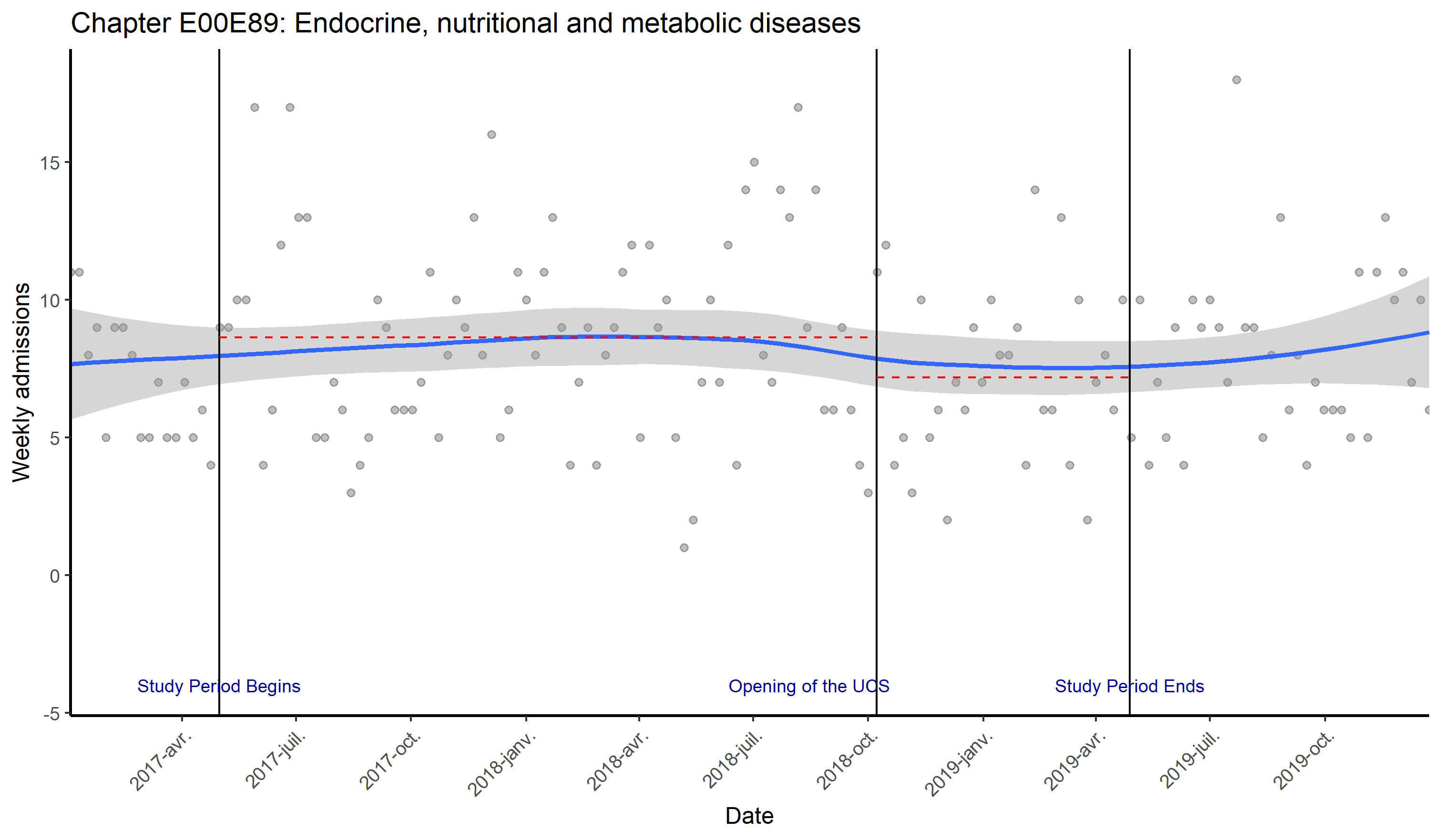** |
| **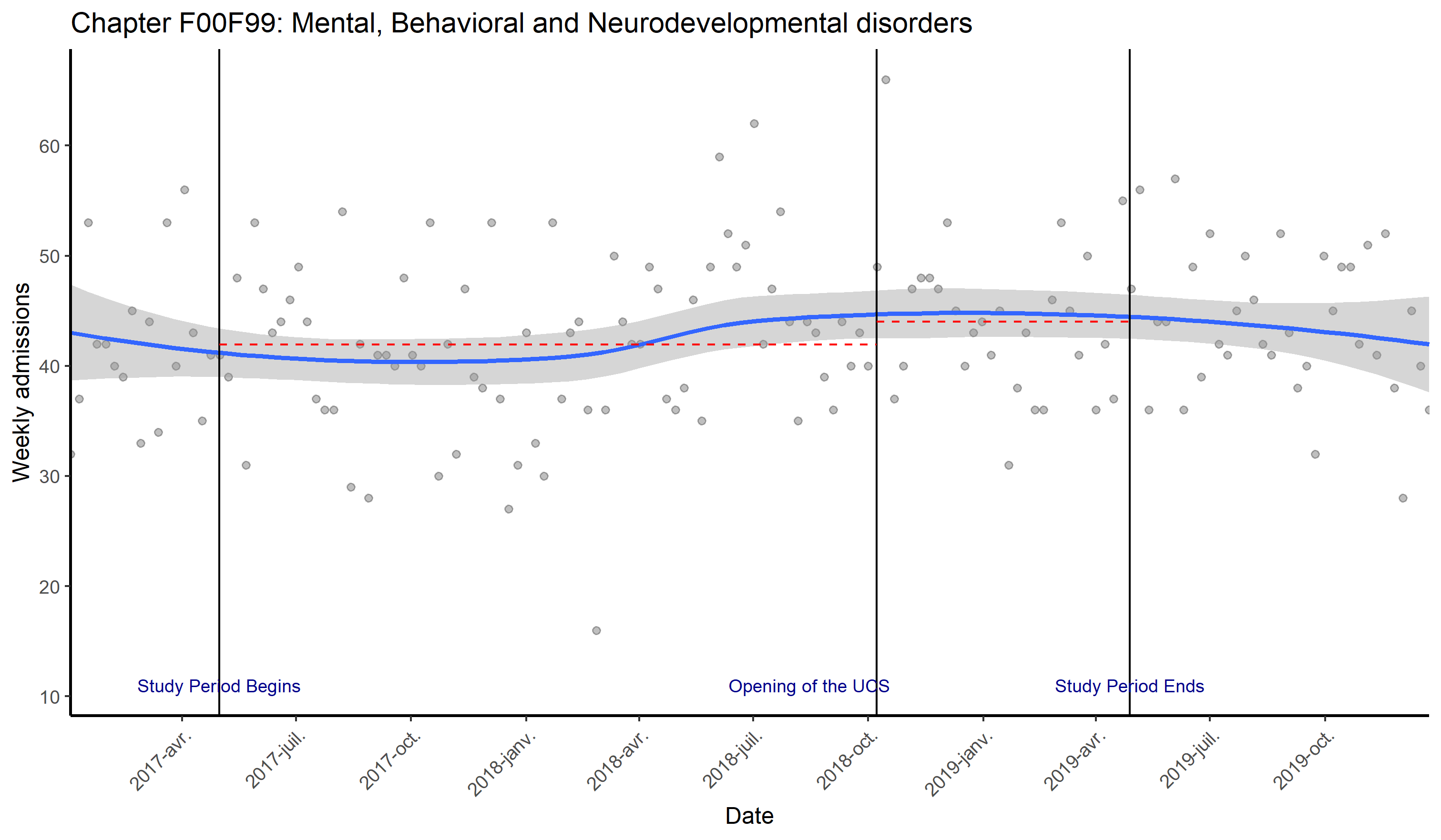** | **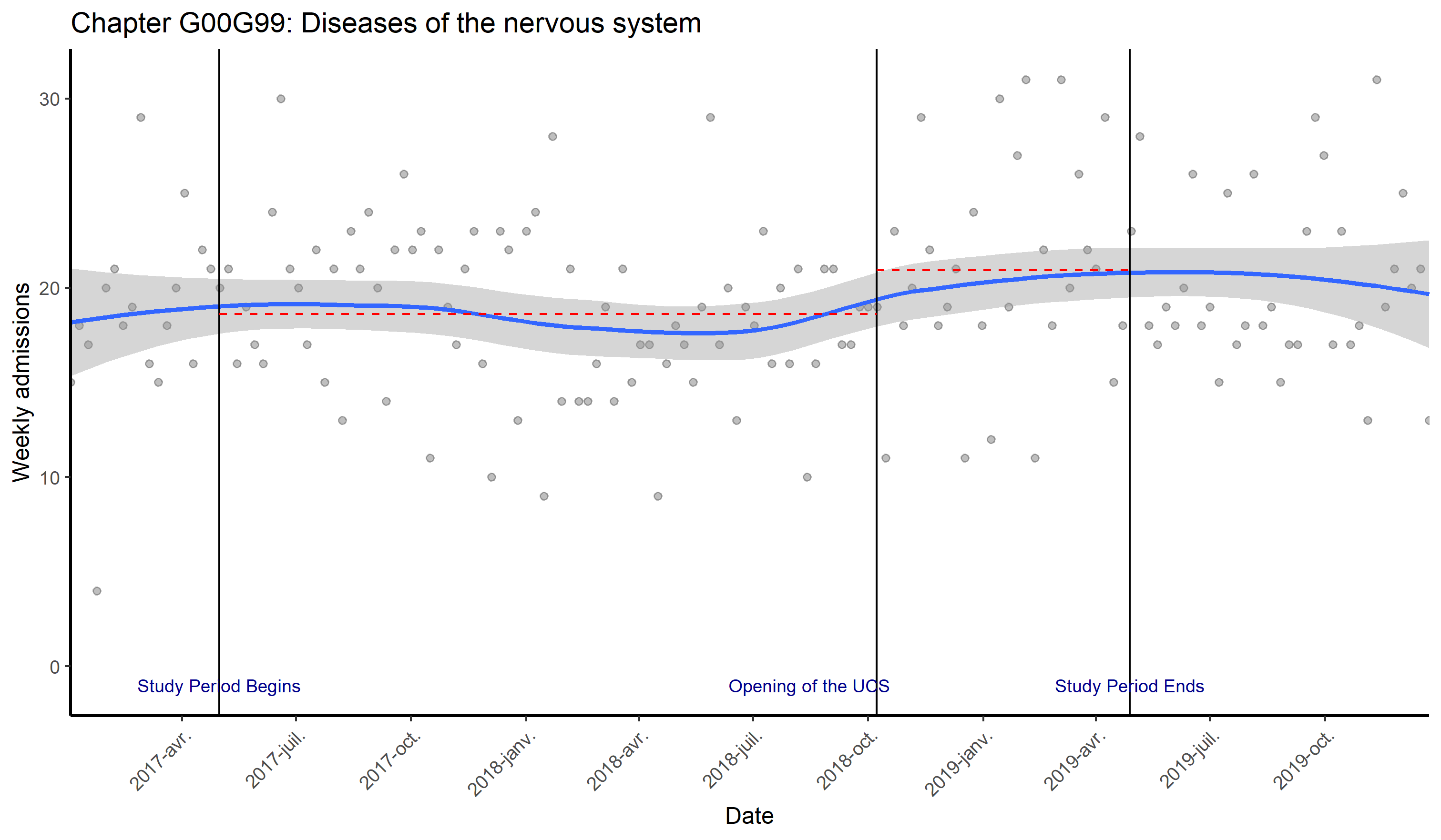** |
| **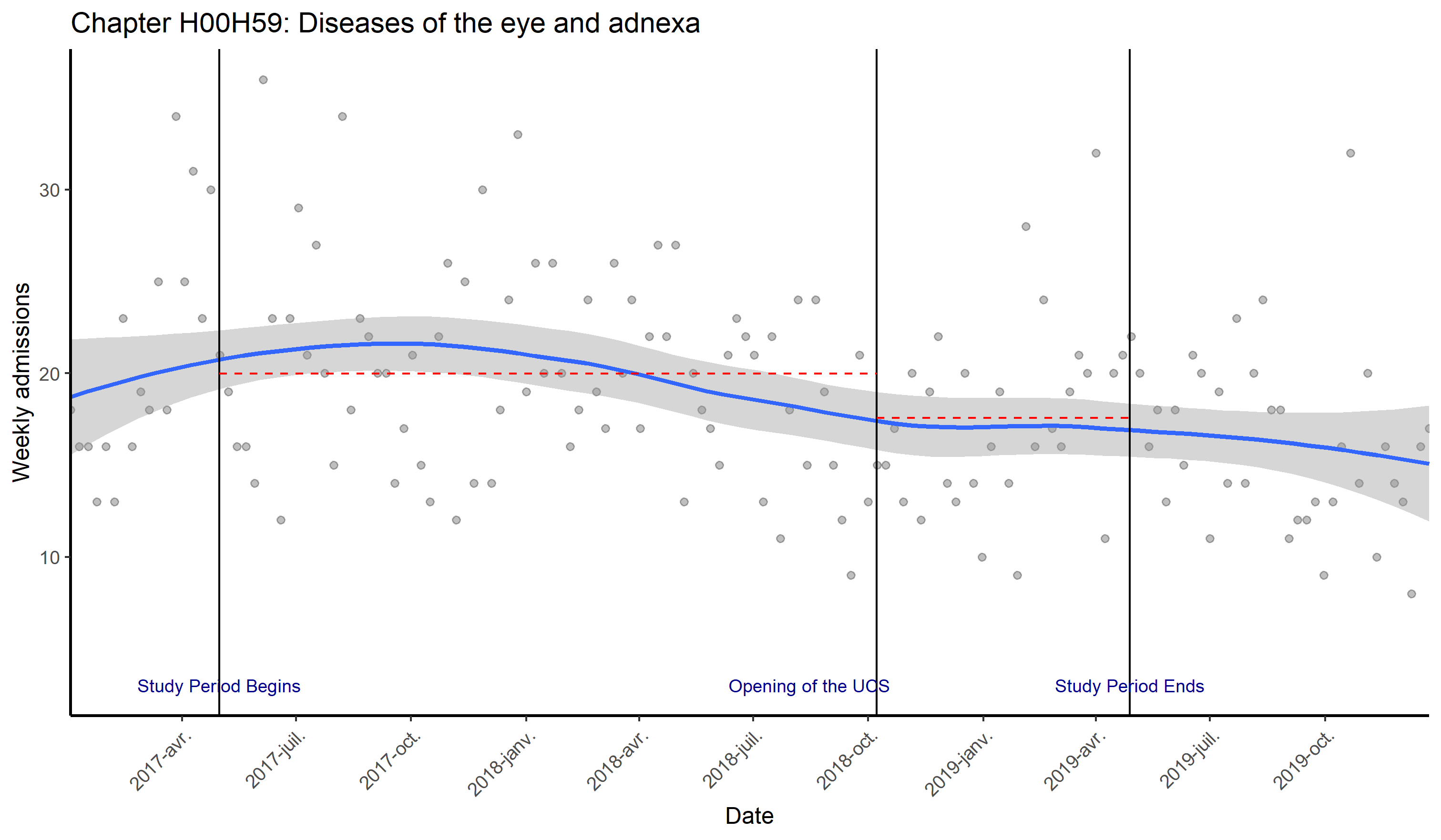** | **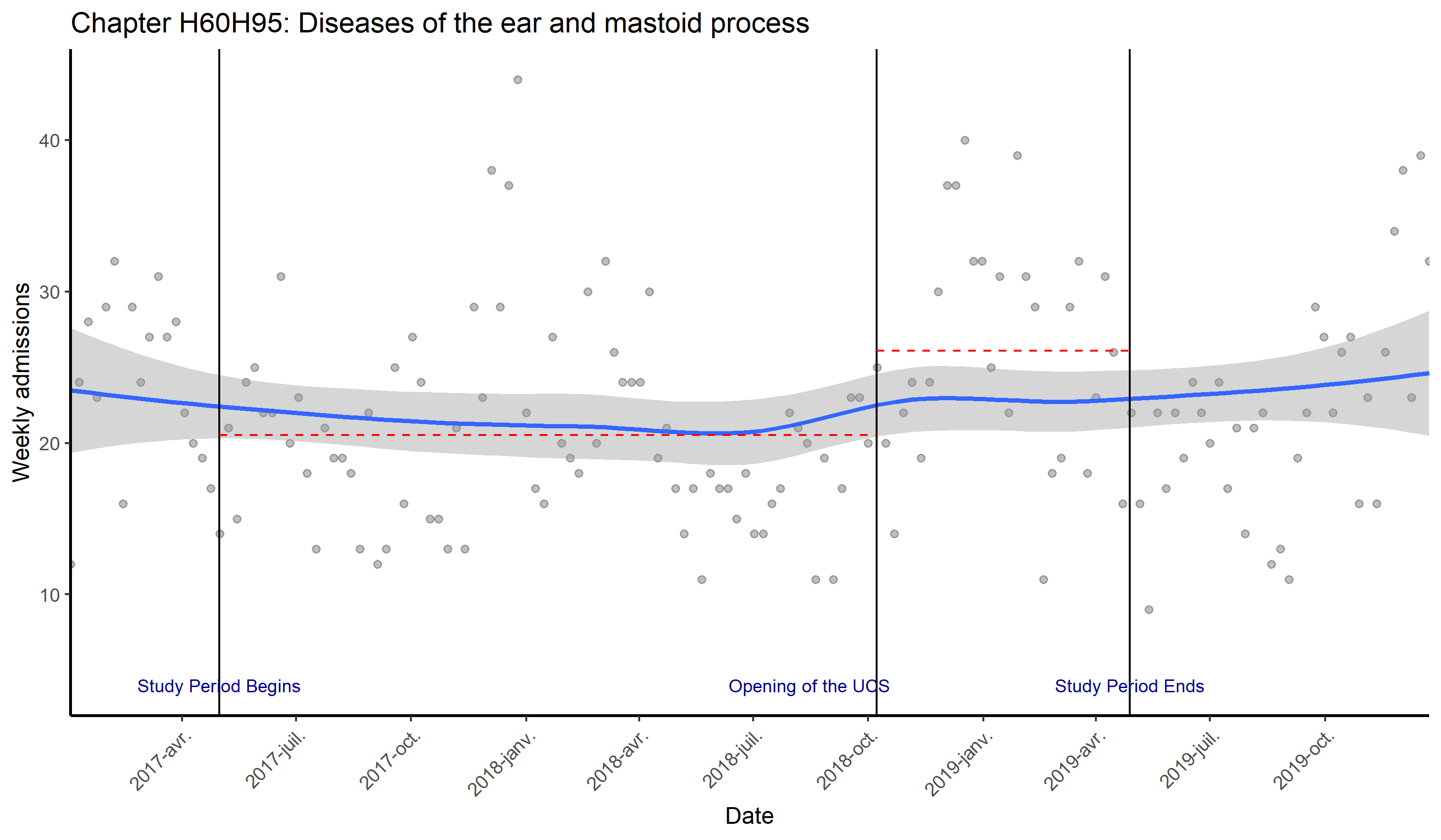** |
| **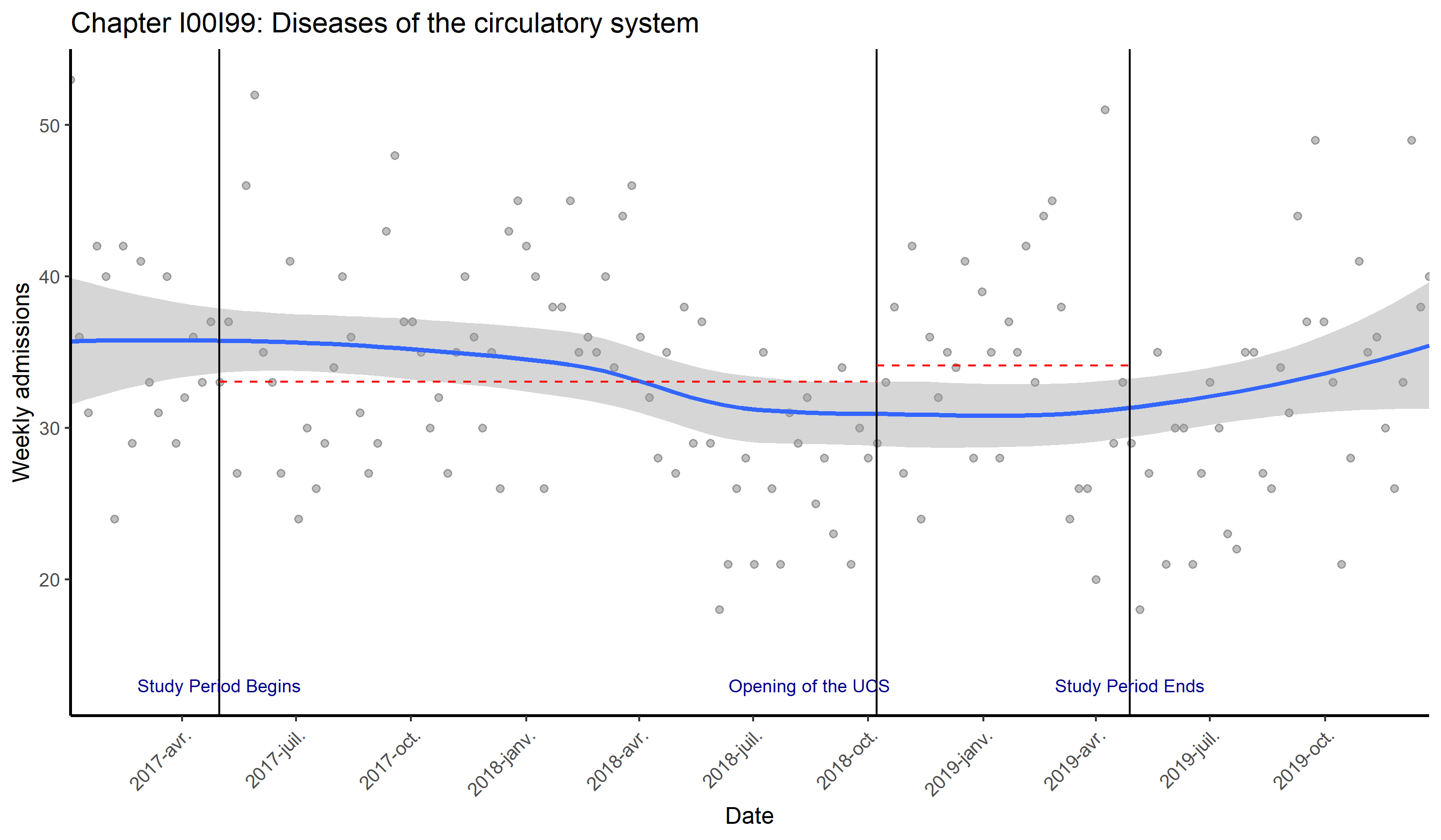** | **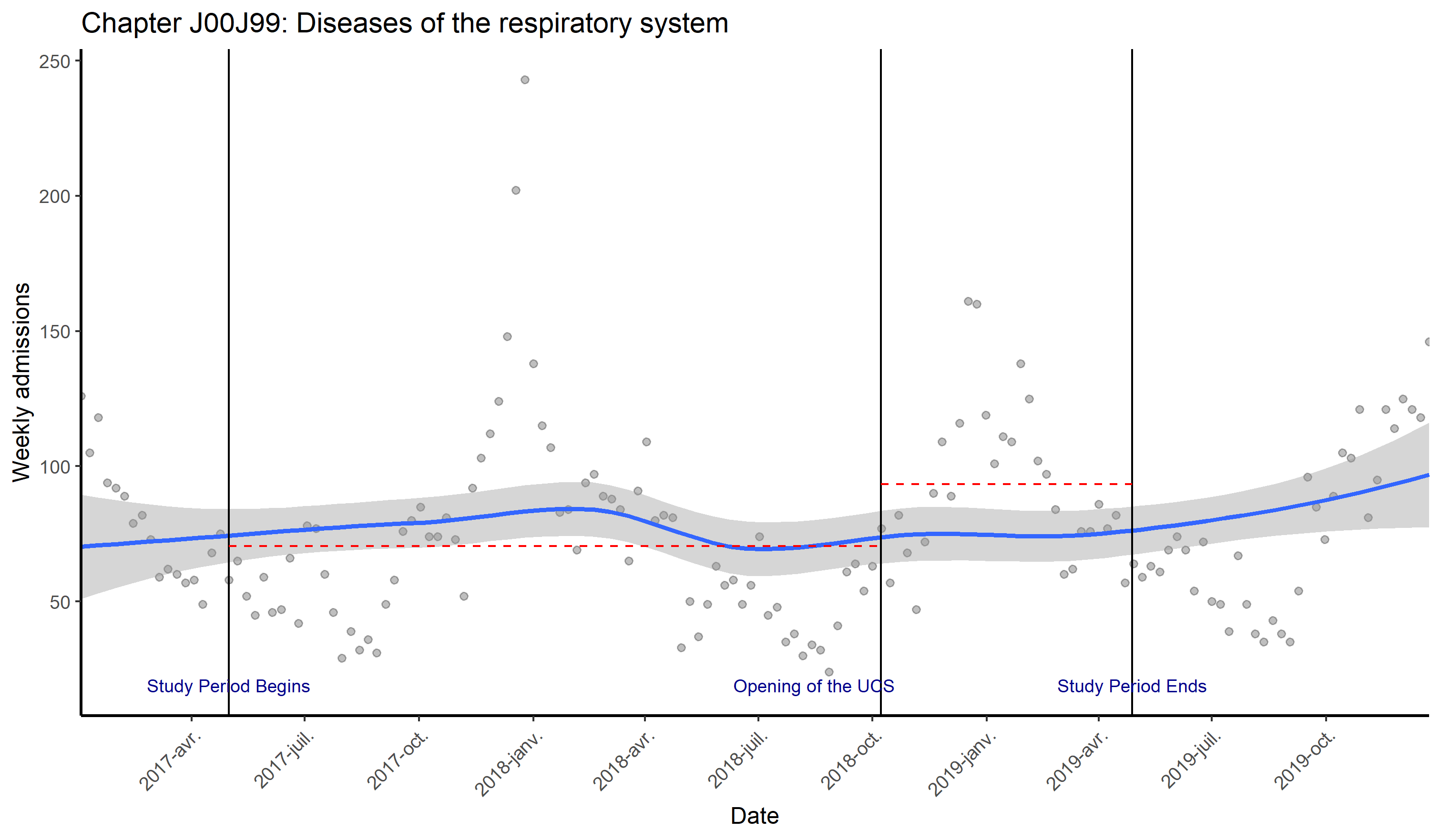** |
| **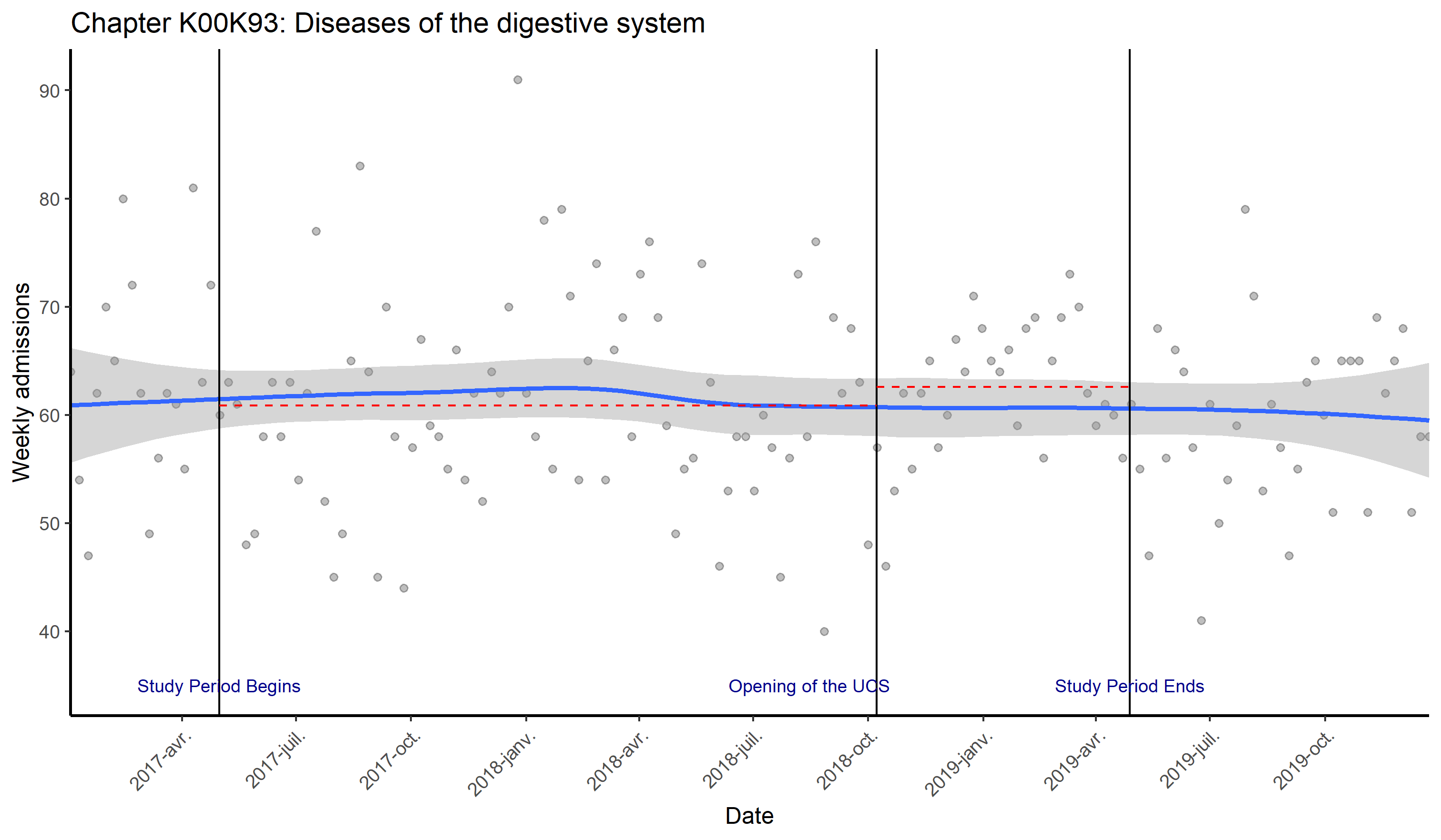** | **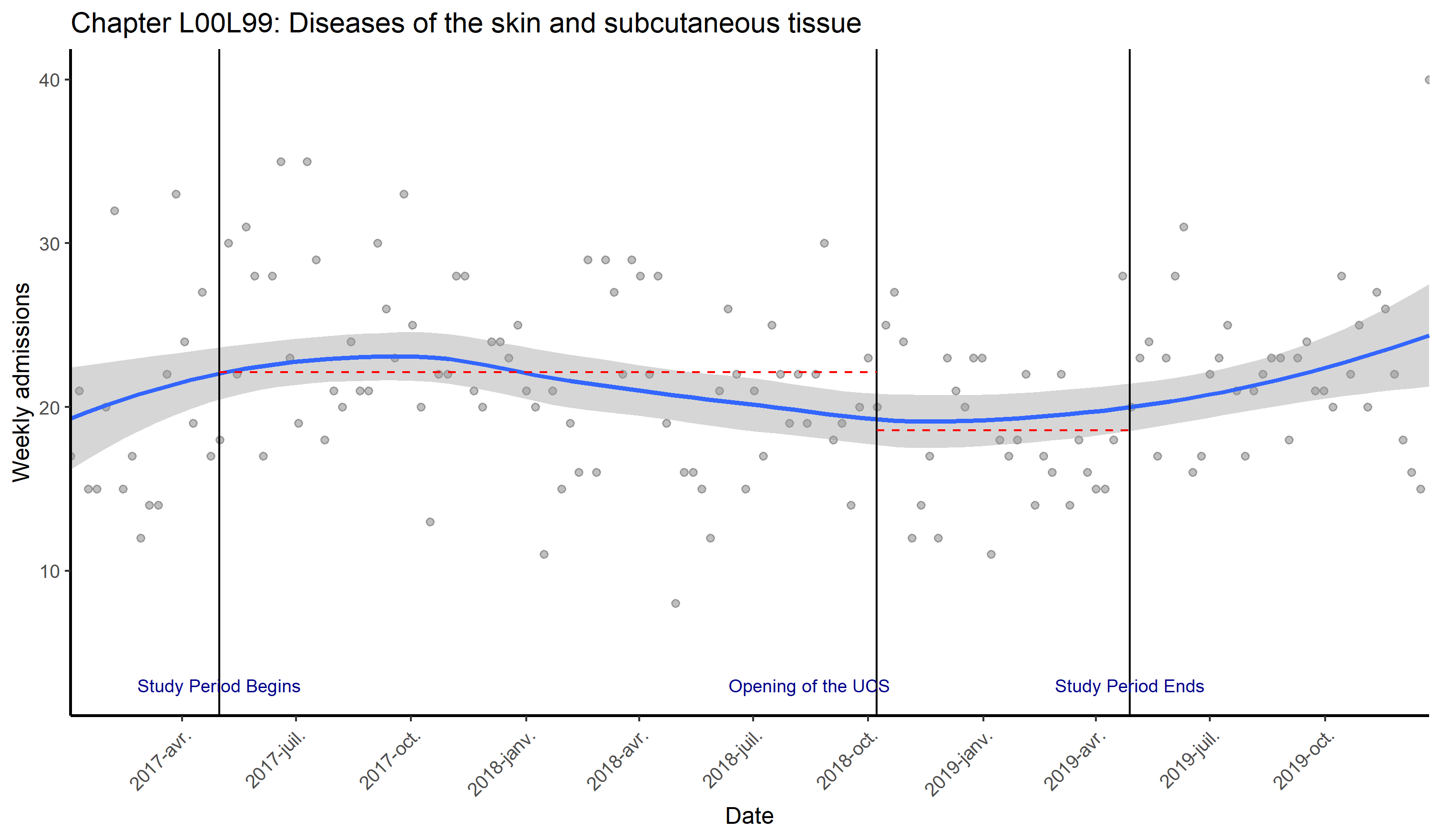** |
| **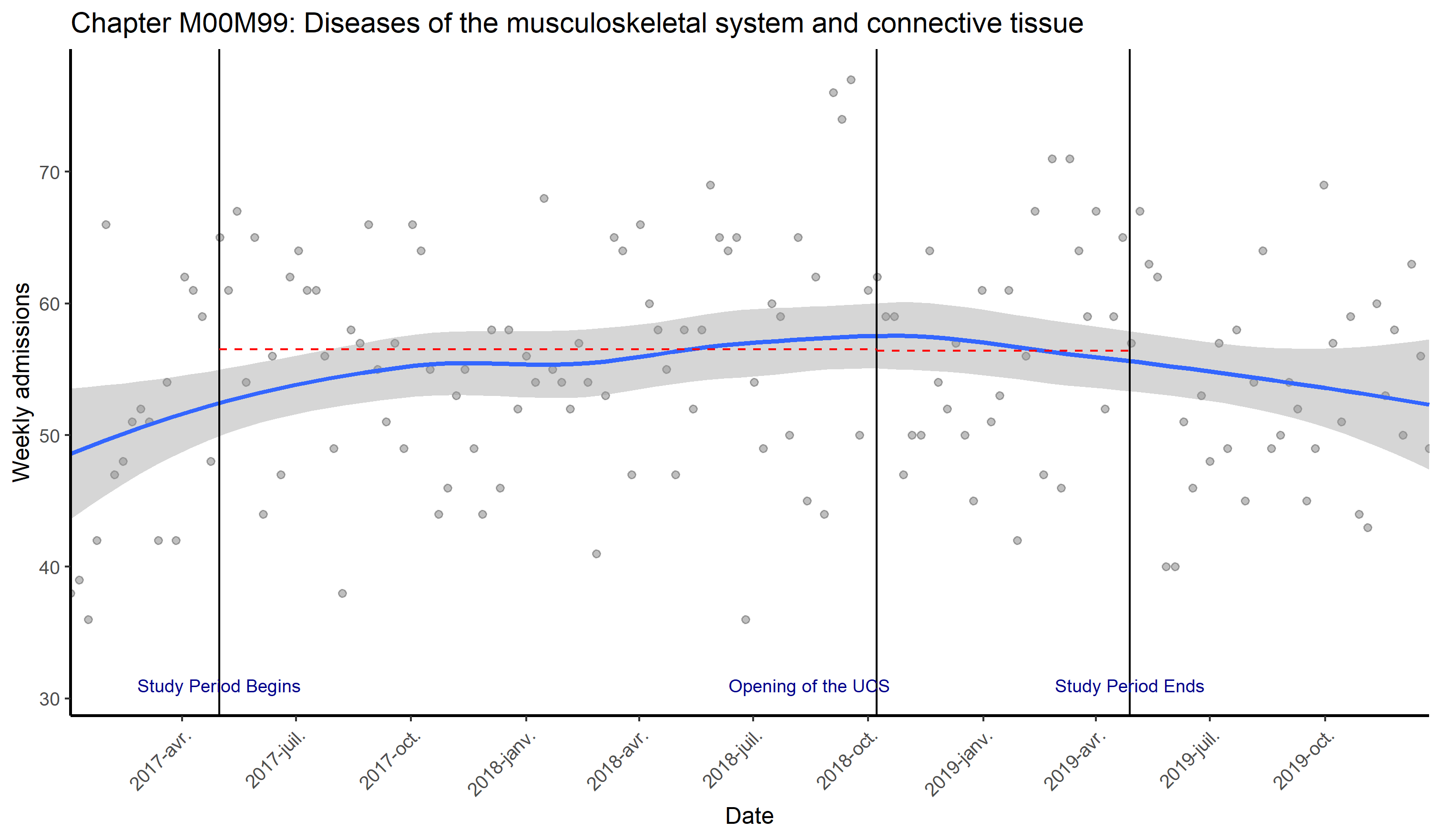** | **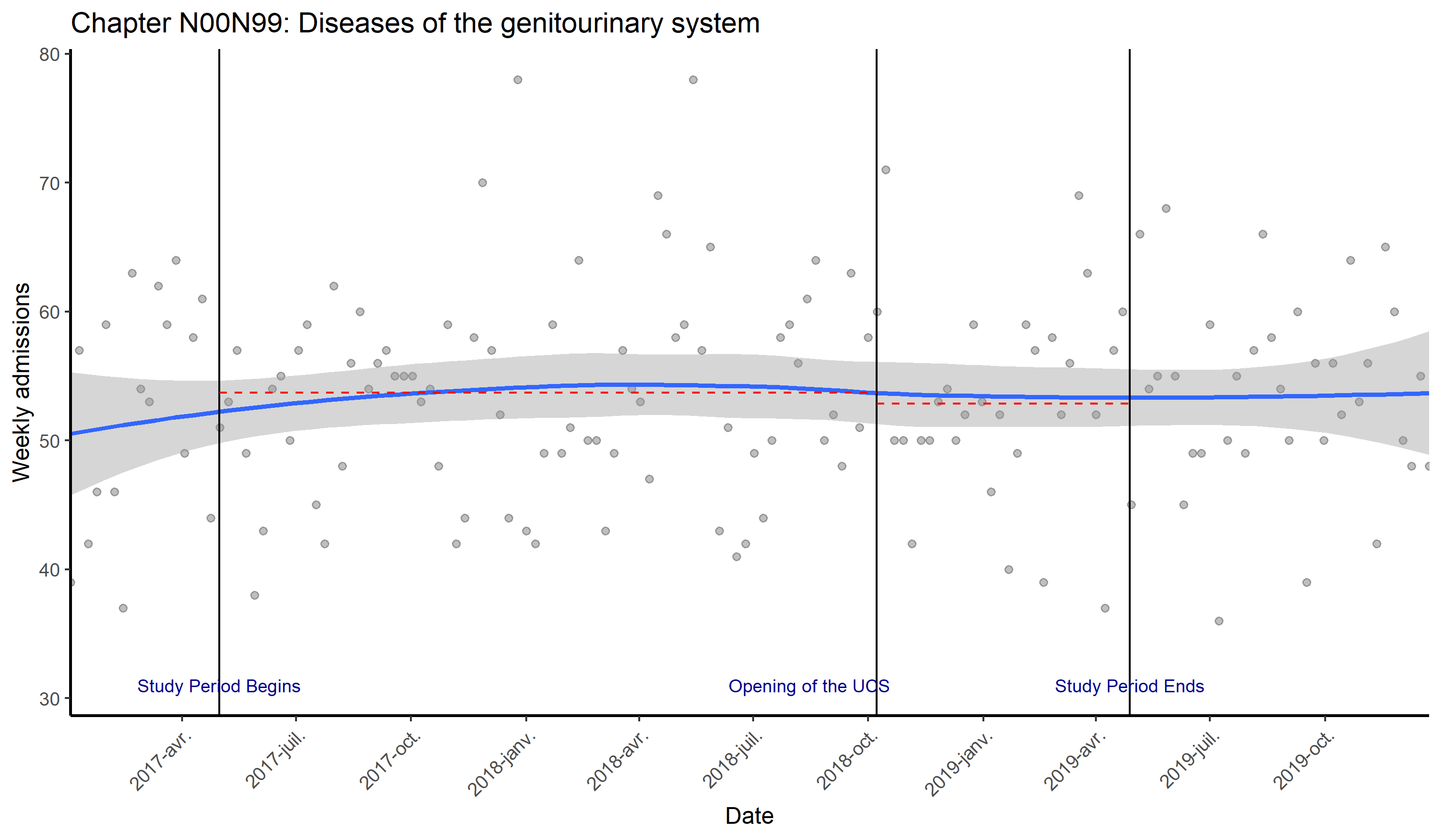** |
| **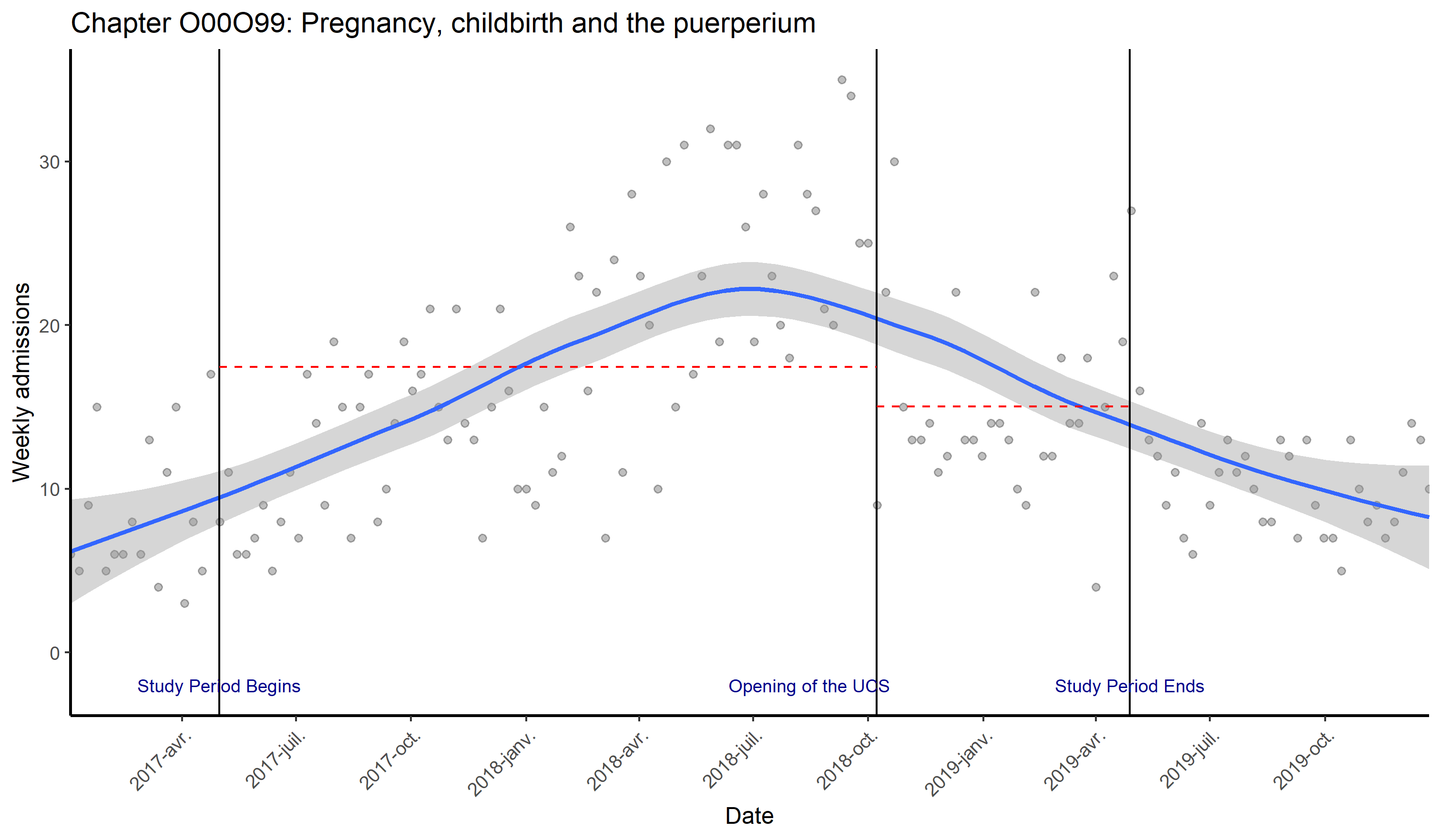** | **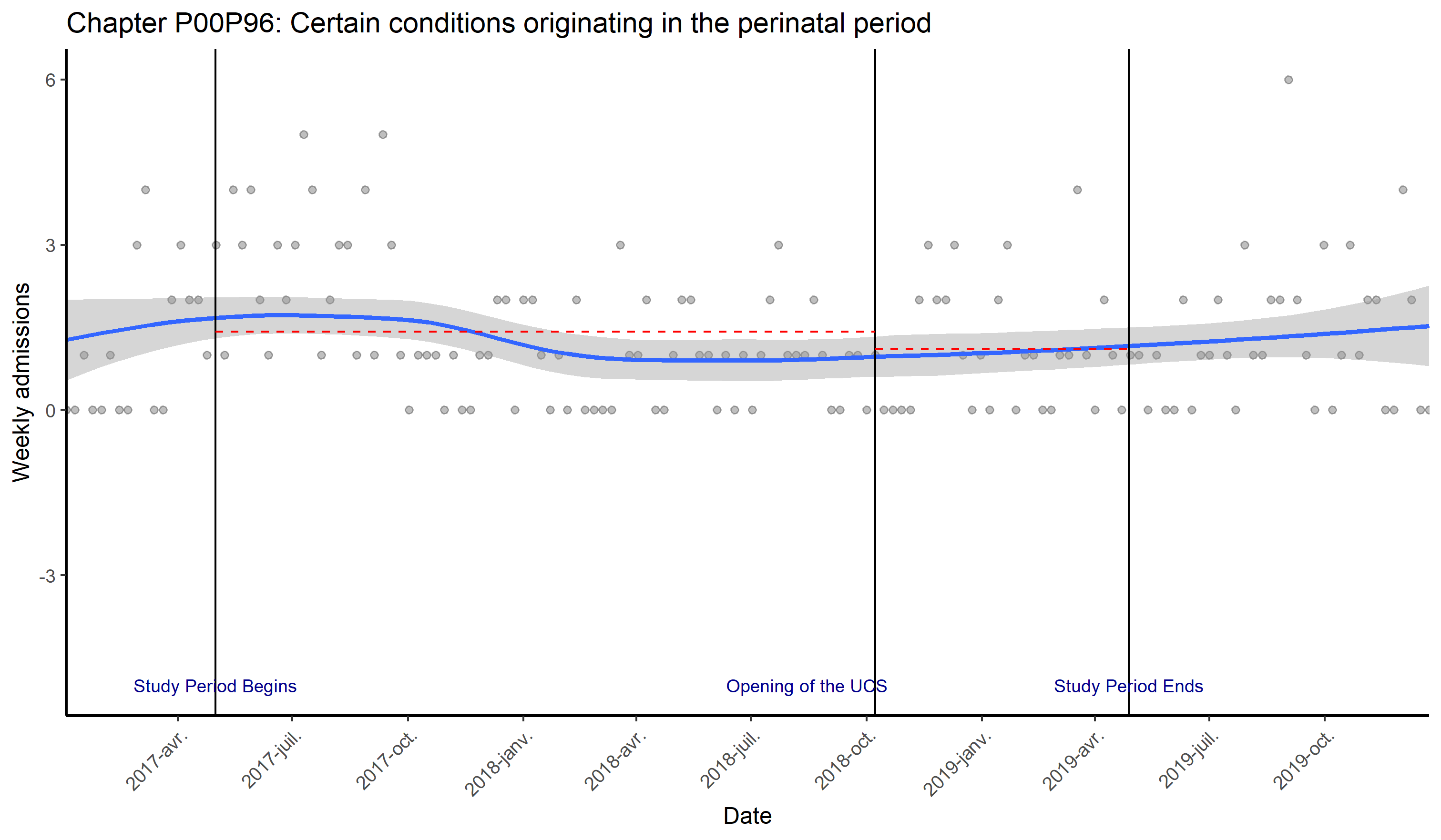** |
| **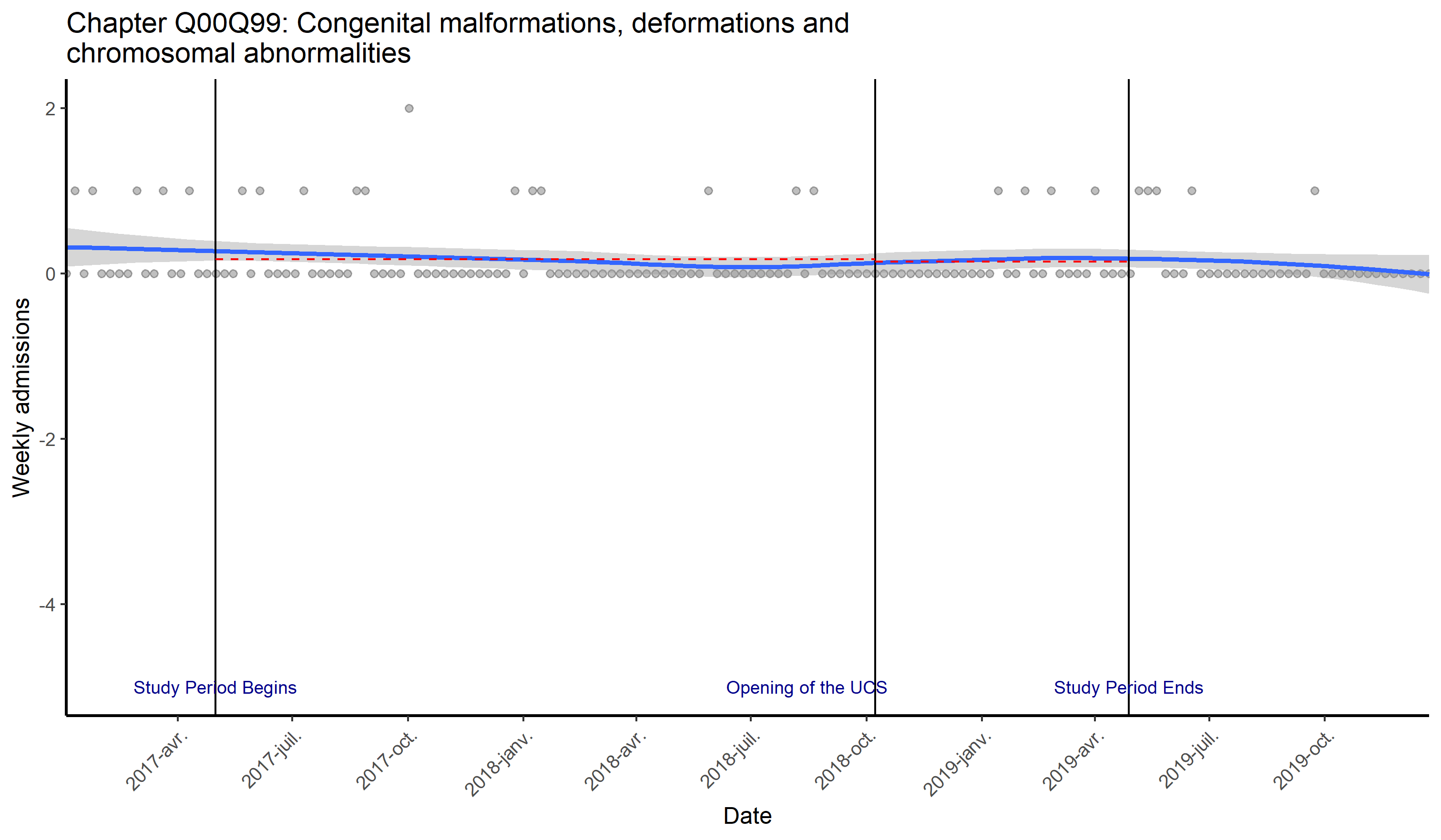** | **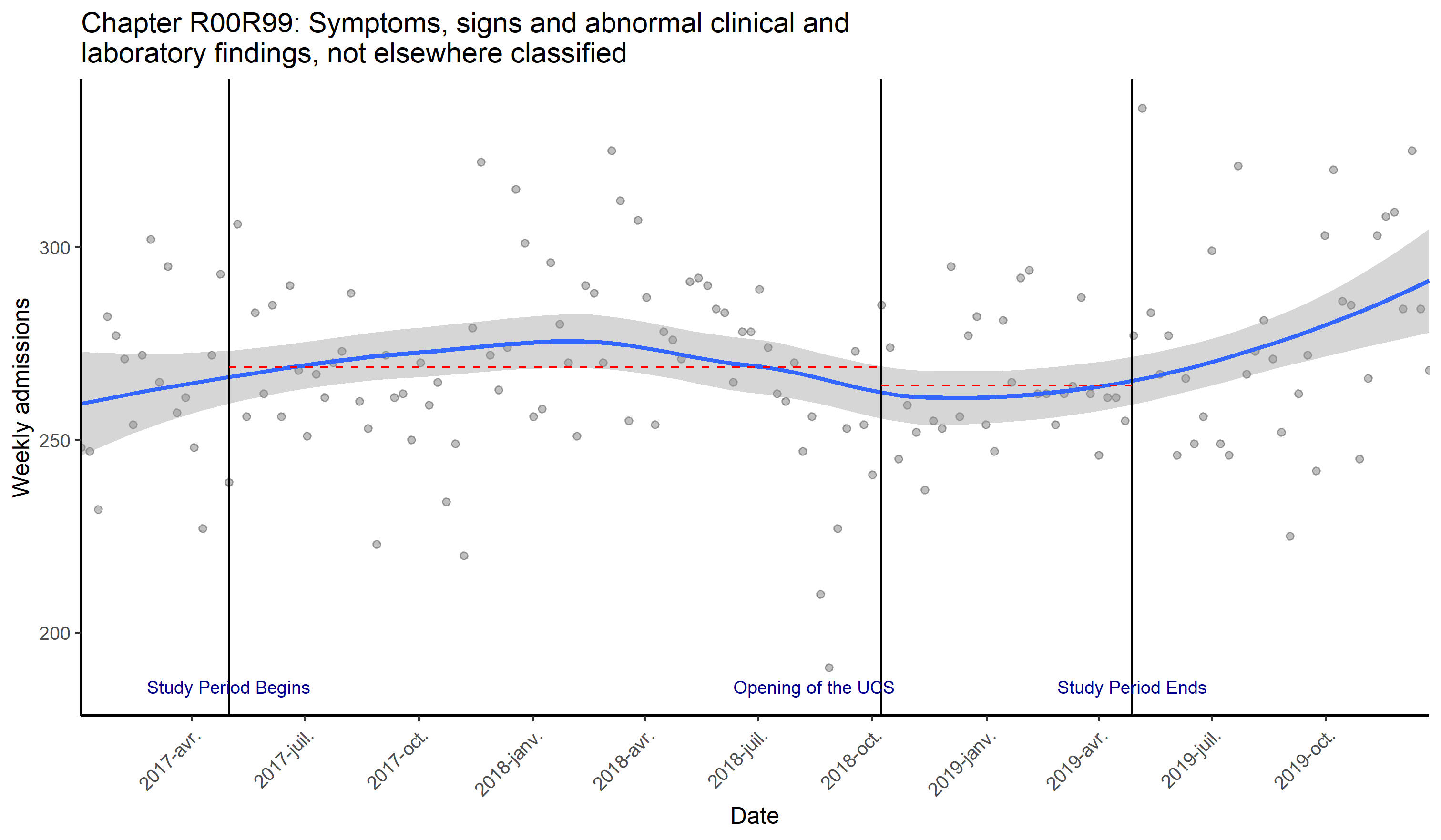** |
| **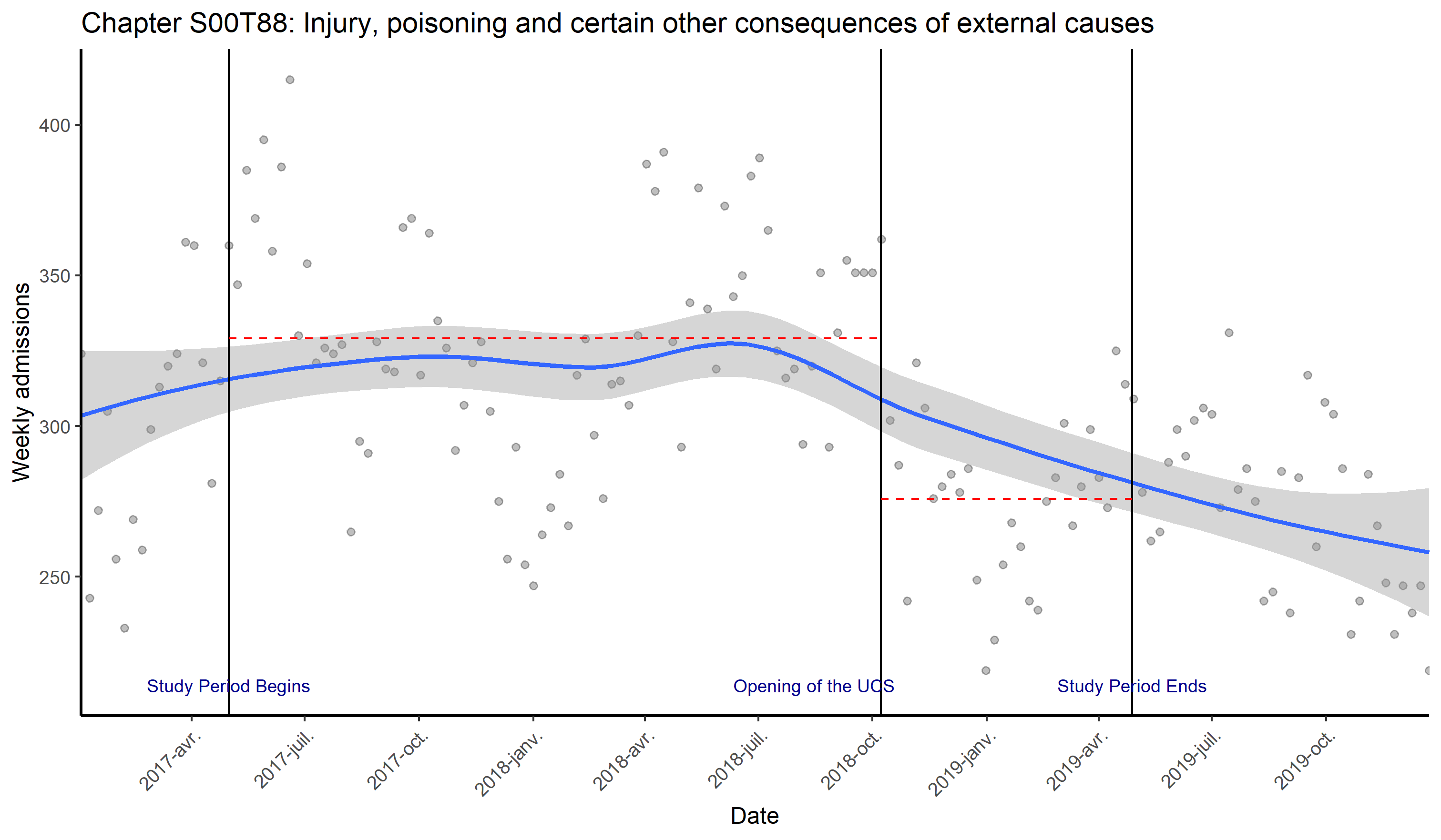** | **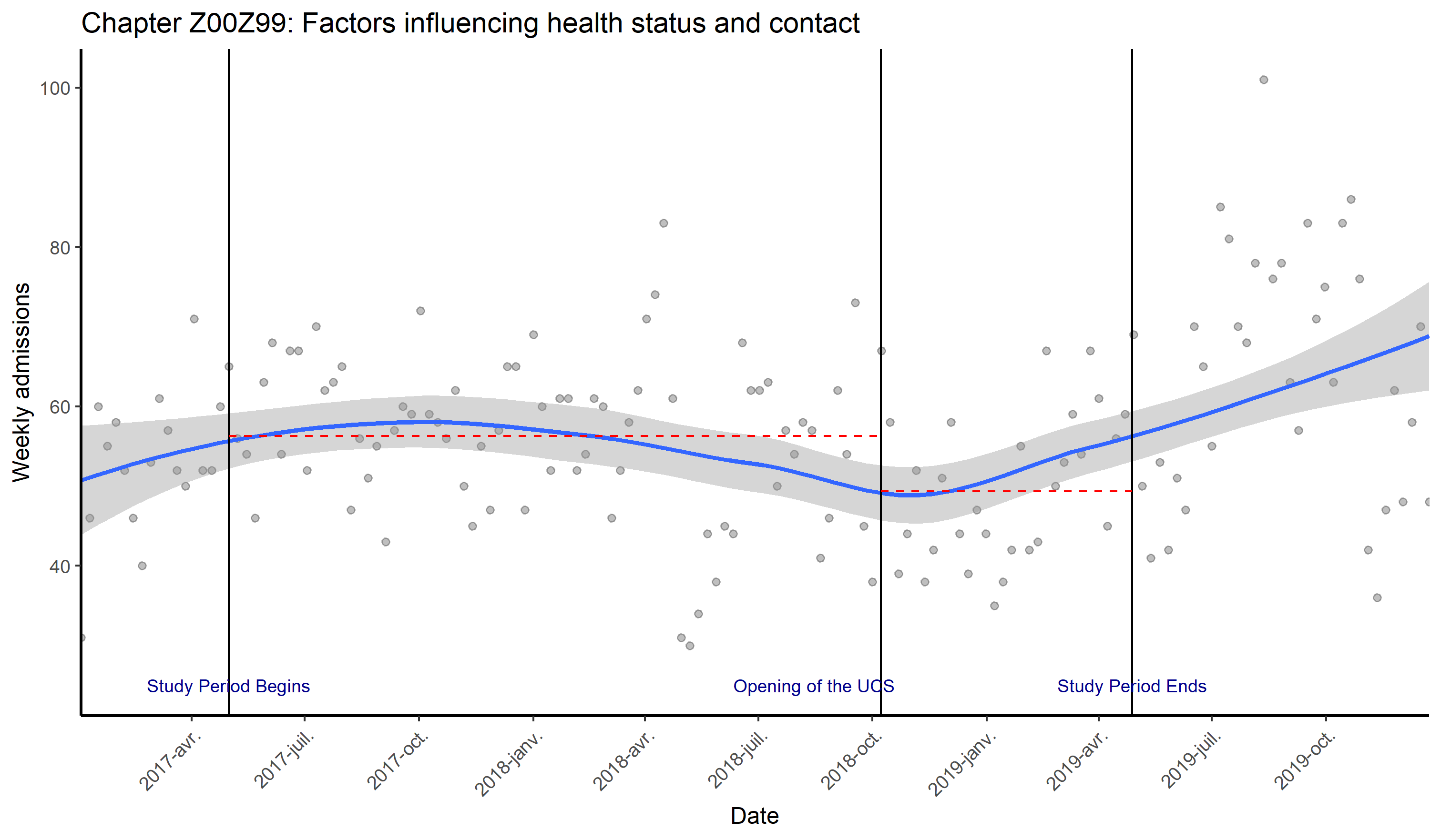** |

*Notes: The gray points are the observed weekly admissions; the blue line corresponds to the local regression (loss) of the weekly admissions and the dotted red lines corresponds to the averages of weekly admissions before and after the opening of the UCS*

**S3 Figure.** Change in weekly admissions to illustrate the trends for the 16-cluster structure and the ICD10 chapter classification structure from 2017 to 2019
