# Supplementary material for: Multimorbidity clustering of the emergency department patient flow: Impact analysis of new unscheduled care clinics
Source: PLoS One. 2022 Jan 31;17(1):e0262914. doi: 10.1371/journal.pone.0262914 (PMC8803184; doi:10.1371/journal.pone.0262914)
Supplement: S1 File — (DOCX) [file pone.0262914.s006.docx]

**Title: Multimorbidity Clustering of the Emergency Department Patient Flow: Impact Analysis of New Unscheduled Care Clinics**

**Authors:** Adrien Wartelle, MSc^1,2,*^, Farah Mourad-Chehade, PhD^1^, Farouk Yalaoui, PhD^1^, Hélène Questiaux, MD^3^, Thomas Monneret, MD^4^, Ghislain Soliveau, MD, PhD^4^, Jan Chrusciel, MD^2^, Antoine Duclos, MD, PhD^5,6,7^, David Laplanche, MD^2^, Stéphane Sanchez, MD, MPH^2^.

^1^ Université Technologique de Troyes, Institut Charles Delaunay, Troyes, F-10000, France

^2^ Centre Hospitalier de Troyes, Health Services and Performance Research Lab, Troyes, F-10000, France

^3^ Centre Hospitalier de Troyes, Emergency Department, Troyes, F-10000, France

^4^ Etablissement Aubois des Soins Immédiats, Troyes, F-10000, France

^5^ Hospices Civils de Lyon, Health Data Department, Lyon, F-69003, France

^6^ Université Claude Bernard Lyon 1, Health Services and Performance Research Lab (HESPER EA7425), F-69008 Lyon, France

^7^ Brigham and Women’s Hospital, Harvard Medical School, Center for Surgery and Public Health, Boston, MA, USA

***Corresponding author:**

**Address:** Centre Hospitalier de Troyes, Health Services and Performance Research Lab,

101 Ave. Anatole France 10000 Troyes, F-10000, France;

**Phone:** +33 3 25 49 48 01

**E-mail:** [adrien.wartelle@ch-troyes.fr](mailto:adrien.wartelle@ch-troyes.fr)

**Complete author information:**

**Adrien Wartelle, MSc, (*Corresponding author)**

PhD Student

Address: Centre Hospitalier de Troyes, Health Services and Performance Research Lab,

101 Ave. Anatole France 10000 Troyes, F-10000, France; Phone: +33 3 25 49 48 01 / E-mail:

adrien.wartelle@ch-troyes.fr

Université Technologique de Troyes, Institut Charles Delaunay, 12 rue Marie Curie, 10000 Troyes, F-10000, France, Phone: +33 3 25 71 56 77, E-mail: [adrien.wartelle@utt.fr](mailto:adrien.wartelle@utt.fr)

**Farad Mourad-Chehade, PhD,**

Associate Professor

Université Technologique de Troyes, Institut Charles Delaunay, 12 rue Marie Curie, 10000 Troyes, F-10000, France, Phone: +33 3 25 71 56 77, E-mail: [farah.chehade@utt.fr](mailto:farah.chehade@utt.fr)

**Farouk Yalaoui, PhD,**

Professor

Université Technologique de Troyes, Institut Charles Delaunay, 12 rue Marie Curie, 10000 Troyes, F-10000, France, Phone: +33 3 25 71 56 26, E-mail: [farouk.yalaoui@utt.fr](mailto:farouk.yalaoui@utt.fr)

**Hélène Questiaux, MD**

Emergency Physician

Address: Centre Hospitalier de Troyes, Emergency Department, 101 Ave. Anatole France 10000 Troyes, F-10000, France; Phone: +33 3 25 49 70 25, E-mail: [helene.questiaux@ch-troyes.fr](mailto:helene.questiaux@ch-troyes.fr)

**Thomas Monneret, MD,**

Emergency Physician

Address: Etablissement Aubois des Soins Immédiats, 4 Rue Chaim Soutine 10000 Troyes, F-10000, France, Phone: +33 3 25 49 49 49, E-mail: thomas.monneret@ch-troyes.fr

**Ghilain Solivau, MD,**

Emergency Physician

Address: Etablissement Aubois des Soins Immédiats, 4 Rue Chaim Soutine 10000 Troyes, F-10000, France, Phone: +33 3 25 49 49 49, E-mail: [drsoliveau@gmail.com](mailto:drsoliveau@gmail.com)

**Jan Chrusciel, MD, MPH**

Public Health Physician,

Address: Centre Hospitalier de Troyes, Health Services and Performance Research Lab,

101 Ave. Anatole France 10000 Troyes, F-10000, France, Phone: +33 3 25 49 48 01,

E-mail: [jan.chrusciel@ch-troyes.fr](mailto:jan.chrusciel@ch-troyes.fr)

**Antoine Duclos, MD, PhD,**

Public Health Physician, Professor,

Address: Hospices Civils de Lyon, Health Data Department, 3 Quai des Célestins 69002 Lyon, F-69002, France, Phone: +33 4 72 11 57 71, E-mail: [antoine.duclos@chu-lyon.fr](mailto:antoine.duclos@chu-lyon.fr)

**David Laplanche, MD,**

Public Health Physician,

Address: Centre Hospitalier de Troyes, Health Services and Performance Research Lab,

101 Av Anatole France 10000 Troyes, F-10000, France, Phone: +33 3 25 49 48 01,

E-mail: [david.laplanche@ch-troyes.fr](mailto:david.laplanche@ch-troyes.fr)

**Stéphane Sanchez, MD, MPH**

Public Health Physician,

Address: Centre Hospitalier de Troyes, Health Services and Performance Research Lab,

101 Av Anatole France 10000 Troyes, F-10000, France, Phone: +33 3 25 49 48 01,

E-mail: stephane.sanchez@ch-troyes.fr

**Supplementary Table S1.** Diagnostic content and quality indicators of the 14 clusters

| Cluster name | Visits : n | Composition (blocks of diagnoses) | RR_intra | RR_inter | MR |
| --- | --- | --- | --- | --- | --- |
| 1 Digestive disorders, pregnancy, menstruation | 17705 | R10-R19 (37.1%); K55-K64 (16.2%); N80-N98 (8.7%); N20-N23 (6.9%); O00-O08 (5.3%); N30-N39 (4.9%); Z30-Z39 (4.1%); O20-O29 (3.8%); K80-K87 (3.8%); N10-N16 (3.7%); N70-N77 (2.1%); K35-K38 (1.3%); J30-J39 (0.6%); P90-P96 (0.6%); N60-N64 (0.5%); O85-O92 (0.3%); A50-A64 (0.3%); O60-O75 (0.2%); Q50-Q56 (0.1%) | 1.48 | 0.77 | 1.91 |
| 2 General symptoms and mental disorders | 14336 | R50-R69 (68.4%); G40-G47 (11.1%); R25-R29 (7.7%); I60-I69 (6.0%); H80-H83 (5.2%); Z55-Z65 (0.9%); F00-F09 (0.6%); R47-R49 (0.3%) | 1.00 | 0.80 | 1.25 |
| 3 Infectious diseases | 14336 | J00-J06 (21.7%); Z70-Z76 (13.8%); J40-J47 (8.6%); H65-H75 (7.8%); J20-J22 (7.4%); A00-A09 (7.3%); K50-K52 (6.1%); T66-T78 (4.4%); K20-K31 (4.2%); L50-L54 (3.6%); B00-B09 (3.4%); R20-R23 (3.4%); L20-L30 (2.2%); H60-H62 (1.8%); B35-B49 (1.6%); H90-H95 (1.1%); B25-B34 (0.9%); B85-B89 (0.5%); P80-P83 (0.1%); P75-P78 (0.1%) | 1.48 | 0.82 | 1.79 |
| 4 General symptoms of chronic conditions | 13893 | R00-R09 (48.4%); J09-J18 (12.8%); I30-I52 (7.9%); E79-E90 (3.5%); D60-D64 (2.6%); D50-D53 (2.5%); E10-E16 (2.3%); I20-I25 (2.2%); N17-N19 (2.0%); I26-I28 (1.8%); K90-K93 (1.6%); J95-J99 (1.5%); I10-I15 (1.5%); D55-D59 (1.4%); K40-K46 (1.3%); G50-G59 (1.3%); J60-J70 (1.1%); J80-J84 (0.9%); K70-K77 (0.7%); R70-R79 (0.6%); I95-I99 (0.6%); G90-G99 (0.5%); K65-K67 (0.3%); H30-H36 (0.3%); L40-L45 (0.2%); B15-B19 (0.2%); B65-B83 (0.2%); C76-C80 (0.1%); G60-G64 (0.1%); M91-M94 (0.1%); D00-D09 (0.1%); R90-R94 (0.1%); R95-R99 (0.1%) | 1.36 | 0.97 | 1.40 |
| 5 Mental disorders and at-risk behaviors | 12139 | R40-R46 (17.9%); Z00-Z13 (15.8%); S50-S59 (15.4%); F10-F19 (11.0%); F40-F49 (10.3%); T36-T50 (8.6%); F30-F39 (7.9%); T51-T65 (3.4%); F20-F29 (3.3%); S10-S19 (3.3%); F90-F98 (2.1%); F60-F69 (0.5%); C81-C96 (0.3%); L10-L14 (0.2%); F80-F89 (0.1%) | 1.51 | 0.91 | 1.65 |
| 6 Wrist and Hand Trauma | 6777 | S60-S69 (100.0%) | 1.00 | 0.57 | 1.75 |
| 7 Head Trauma | 6112 | S00-S09 (100.0%) | 1.00 | 0.44 | 2.27 |
| 8 Hip related trauma and disorders | 5781 | S70-S79 (21.2%); R30-R39 (14.6%); A30-A49 (11.0%); T20-T32 (9.7%); N40-N51 (9.3%); I80-I89 (7.7%); T79-T79 (6.6%); T80-T88 (5.8%); I70-I79 (3.6%); Z40-Z54 (3.3%); D65-D69 (2.4%); Z80-Z99 (1.2%); D37-D48 (1.0%); C15-C26 (0.7%); D10-D36 (0.7%); H20-H22 (0.4%); G35-G37 (0.3%); E28-E30 (0.3%); M95-M99 (0.2%) | 1.90 | 1.03 | 1.85 |
| 9 Feet Trauma | 5224 | S90-S99 (98.5%); M80-M90 (1.5%) | 2.18 | 0.60 | 3.65 |
| 10 Back and Spine disorders | 3777 | M40-M54 (79.7%); S30-S39 (20.4%) | 1.26 | 0.61 | 2.06 |
| 11 Occulomotor disorders | 3235 | T15-T19 (37.0%); H10-H13 (22.6%); H55-H59 (15.9%); H53-H54 (9.0%); H15-H19 (6.6%); H00-H06 (5.3%); B50-B64 (1.0%); H43-H45 (1.0%); G70-G73 (0.9%); G80-G83 (0.5%); N00-N08 (0.4%) | 1.29 | 0.52 | 2.47 |
| 12 Lower limb trauma | 3074 | S80-S89 (100.0%) | 1.00 | 0.64 | 1.56 |
| 13 Cutaneous infections, wounds, and skin disorders | 2787 | L00-L08 (38.9%); Z20-Z29 (22.5%); K00-K14 (21.1%); L60-L75 (4.6%); L80-L99 (2.7%); F50-F59 (2.2%); O30-O48 (1.7%); G20-G26 (1.7%); E00-E07 (1.2%); G00-G09 (1.0%); A20-A28 (0.8%); L55-L59 (0.7%); A65-A69 (0.5%); A80-A89 (0.4%) | 1.75 | 0.80 | 2.18 |
| 14 Arthropathies | 2760 | M60-M79 (70.9%); M00-M25 (29.2%) | 1.88 | 0.71 | 2.63 |
| 15 Shoulder and arm trauma | 1930 | S40-S49 (100.0%) | 1.00 | 0.55 | 1.82 |
| 16 Chest trauma and other diseases of the pleura | 1151 | S20-S29 (81.1%); J90-J94 (18.9%) | 2.79 | 0.82 | 3.42 |

*RR_intra_, RR_inter_ and MR are quality indicators of the clusters. They are described in the method section and in the original article of the clustering method [1]

1. Wartelle A, Mourad-Chehade F, Yalaoui F, Chrusciel J, Laplanche D, Sanchez S. Clustering of a Health Dataset Using Diagnosis Co-Occurrences. Appl Sci. 2021;11:2373. doi:10.3390/app11052373.

**Supplementary Data Table S2.** Labels of the ICD10 Blocks with content of each block from the ICD10 chapter classification structure

| ICD10 Block | Label |  |
| --- | --- | --- |
| A00A09 | Intestinal infectious diseases | |
| A20A28 | Certain zoonotic bacterial diseases | |
| A30A49 | Other bacterial diseases | |
| A50A64 | Infections with a predominantly sexual mode of transmission | |
| A65A69 | Other spirochetal diseases | |
| A80A89 | Viral infections of the central nervous system | |
| B00B09 | Viral infections characterized by skin and mucous membrane lesions | |
| B15B19 | Viral hepatitis | |
| B25B34 | Other viral diseases | |
| B35B49 | Mycoses |  |
| B50B64 | Protozoal diseases | |
| B65B83 | Helminthiases | |
| B85B89 | Pediculosis, acariasis and other infestations | |
| C15C26 | Malignant neoplasms, digestive organs | |
| C76C80 | Malignant neoplasms, secondary and ill-defined | |
| C81C96 | Malignant neoplasms, stated or presumed to be primary, of lymphoid, hematopoietic and related tissue | |
| D00D09 | In situ neoplasms | |
| D10D36 | Benign neoplasms | |
| D37D48 | Neoplasms of uncertain or unknown behavior | |
| D50D53 | Nutritional anemia | |
| D55D59 | Hemolytic anemia | |
| D60D64 | Aplastic and other anemia | |
| D65D69 | Coagulation defects, purpura and other hemorrhagic conditions | |
| E00E07 | Thyroid gland / Thyroid | |
| E10E16 | Pancreas / Insulin, glucagon | |
| E28E30 | Gonads / Estrogen, androgens, testosterone, etc. | |
| E79E90 | Other metabolic disorders | |
| F00F09 | Organic, including symptomatic, mental disorders | |
| F10F19 | Mental and behavioral disorders due to psychoactive substance use | |
| F20F29 | Schizophrenia, schizotypal and delusional disorders | |
| F30F39 | Mood (affective) disorders | |
| F40F49 | Neurotic, stress-related and somatoform disorders | |
| F50F59 | Behavioral syndromes associated with physiological disturbances and physical factors | |
| F60F69 | Disorders of adult personality and behavior | |
| F80F89 | Disorders of psychological development | |
| F90F98 | Behavioral and emotional disorders with onset usually occurring in childhood and adolescence | |
| G00G09 | Inflammatory diseases of the central nervous system | |
| G20G26 | Extrapyramidal and movement disorders | |
| G35G37 | Demyelinating diseases of the central nervous system | |
| G40G47 | Episodic and paroxysmal disorders | |
| G50G59 | Nerve, nerve root and plexus disorders | |
| G60G64 | Polyneuropathies and other disorders of the peripheral nervous system | |
| G70G73 | Diseases of myoneural junction and muscle | |
| G80G83 | Cerebral palsy and other paralytic syndromes | |
| G90G99 | Other disorders of the nervous system | |
| H00H06 | Disorders of eyelid, lacrimal system and orbit | |
| H10H13 | Disorders of conjunctiva | |
| H15H19 | Disorders of sclera and cornea | |
| H20H22 | Disorders of iris and ciliary body | |
| H30H36 | Disorders of choroid and retina | |
| H43H45 | Disorders of vitreous body and globe | |
| H53H54 | Disorders of ocular muscles, binocular movement, accommodation and refraction | |
| H55H59 | Other disorders of eye and adnexa | |
| H60H62 | Diseases of external ear | |
| H65H75 | Diseases of middle ear and mastoid | |
| H80H83 | Diseases of inner ear | |
| H90H95 | Other disorders of ear | |
| I10I15 | Hypertensive diseases | |
| I20I25 | Ischemic heart diseases | |
| I26I28 | Pulmonary heart disease and diseases of pulmonary circulation | |
| I30I52 | Other forms of heart disease | |
| I60I69 | Cerebrovascular diseases | |
| I70I79 | Diseases of arteries, arterioles and capillaries | |
| I80I89 | Diseases of veins, lymphatic vessels and lymph nodes, not elsewhere classified | |
| I95I99 | Other and unspecified disorders of the circulatory system | |
| J00J06 | Acute upper respiratory infections | |
| J09J18 | Influenza and Pneumonia | |
| J20J22 | Other acute lower respiratory infections | |
| J30J39 | Other diseases of upper respiratory tract | |
| J40J47 | Chronic lower respiratory diseases | |
| J60J70 | Lung diseases due to external agents | |
| J80J84 | Other respiratory diseases principally affecting the interstitium | |
| J90J94 | Other diseases of pleura | |
| J95J99 | Other diseases of the respiratory system | |
| K00K14 | Diseases of oral cavity, salivary glands and jaws | |
| K20K31 | Diseases of esophagus, stomach and duodenum | |
| K35K38 | Diseases of appendix | |
| K40K46 | Hernia |  |
| K50K52 | Noninfective enteritis and colitis | |
| K55K64 | Other diseases of intestines | |
| K65K67 | Diseases of peritoneum | |
| K70K77 | Diseases of liver | |
| K80K87 | Disorders of gallbladder, biliary tract and pancreas | |
| K90K93 | Other diseases of the digestive system | |
| L00L08 | Infections of the skin and subcutaneous tissue | |
| L10L14 | Bullous disorders | |
| L20L30 | Dermatitis and eczema | |
| L40L45 | Papulosquamous disorders | |
| L50L54 | Urticaria and erythema | |
| L55L59 | Radiation-related disorders of the skin and subcutaneous tissue | |
| L60L75 | Radiation-related disorders of the skin and subcutaneous tissue | |
| L80L99 | Other disorders of the skin and subcutaneous tissue | |
| M00M25 | Arthropathies | |
| M40M54 | Dorsopathies | |
| M60M79 | Soft tissue disorders | |
| M80M90 | Osteopathies | |
| M91M94 | Chondropathies | |
| M95M99 | Other disorders of the musculoskeletal system and connective tissue | |
| N00N08 | Glomerular diseases | |
| N10N16 | Renal tubulo-interstitial diseases | |
| N17N19 | Renal failure | |
| N20N23 | Urolithiasis |  |
| N30N39 | Other diseases of urinary system | |
| N40N51 | Diseases of male genital organs | |
| N60N64 | Disorders of breast | |
| N70N77 | Inflammatory diseases of female pelvic organs | |
| N80N98 | Noninflammatory disorders of female genital tract | |
| O00O08 | Pregnancy with abortive outcome | |
| O20O29 | Other maternal disorders predominantly related to pregnancy | |
| O30O48 | Maternal care related to the fetus and amniotic cavity and possible delivery problems | |
| O60O75 | Complications of labor and delivery | |
| O85O92 | Complications predominantly related to the puerperium | |
| P75P78 | Digestive system disorders of fetus and newborn | |
| P80P83 | Conditions involving the integument and temperature regulation of fetus and newborn | |
| P90P96 | Other disorders originating in the perinatal period | |
| Q50Q56 | Congenital malformations and deformations : genital organs | |
| R00R09 | Circulatory and respiratory systems | |
| R10R19 | Digestive system and abdomen | |
| R20R23 | Skin and subcutaneous tissue | |
| R25R29 | Nervous and musculoskeletal systems | |
| R30R39 | Urinary system | |
| R40R46 | Cognition, perception, emotional state and behavior | |
| R47R49 | Speech and voice | |
| R50R69 | General symptoms and signs | |
| R70R79 | On examination of blood, without diagnosis | |
| R90R94 | On diagnostic imaging and in function studies, without diagnosis | |
| R95R99 | Ill-defined and unknown causes of mortality | |
| S00S09 | Injury: Head | |
| S10S19 | Injury: Neck |  |
| S20S29 | Injury: Thorax | |
| S30S39 | Injury: Abdomen, lower back, lumbar spine and pelvis | |
| S40S49 | Injury: Shoulder and upper arm | |
| S50S59 | Injury: Elbow and forearm | |
| S60S69 | Injury: Wrist and hand | |
| S70S79 | Injury: Hip and thigh | |
| S80S89 | Injury: Knee and lower leg | |
| S90S99 | Injury: Ankle and foot | |
| T15T19 | Effects of foreign body entering through natural orifice | |
| T20T32 | Burns and corrosions | |
| T36T50 | Poisoning by drugs, medicaments and biological substances | |
| T51T65 | Toxic effects of substances chiefly non-medicinal as to source | |
| T66T78 | Other and unspecified effects of external causes | |
| T79T79 | Certain early complications of trauma | |
| T80T88 | Complications of surgical and medical care, not elsewhere classified | |
| Z00Z13 | Persons encountering health services for examination and investigation | |
| Z20Z29 | Persons with potential health hazards related to communicable diseases | |
| Z30Z39 | Persons encountering health services in circumstances related to reproduction | |
| Z40Z54 | Persons encountering health services for specific procedures and health care | |
| Z55Z65 | Persons with potential health hazards related to socioeconomic and psychosocial circumstances | |
| Z70Z76 | Persons encountering health services in other circumstances | |
| Z80Z99 | Persons with potential health hazards related to family and personal history and certain conditions influencing health status | |

| **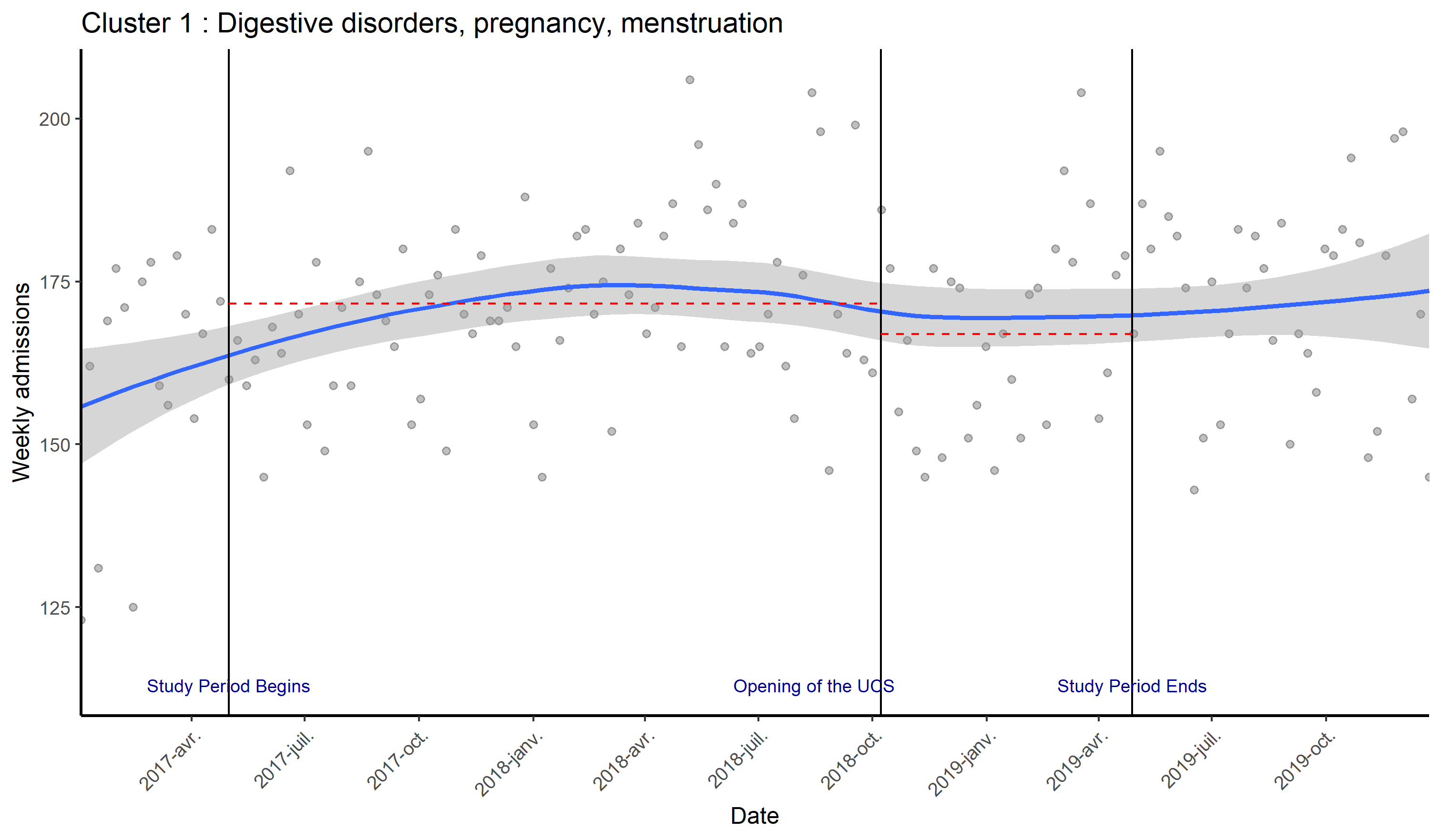** | 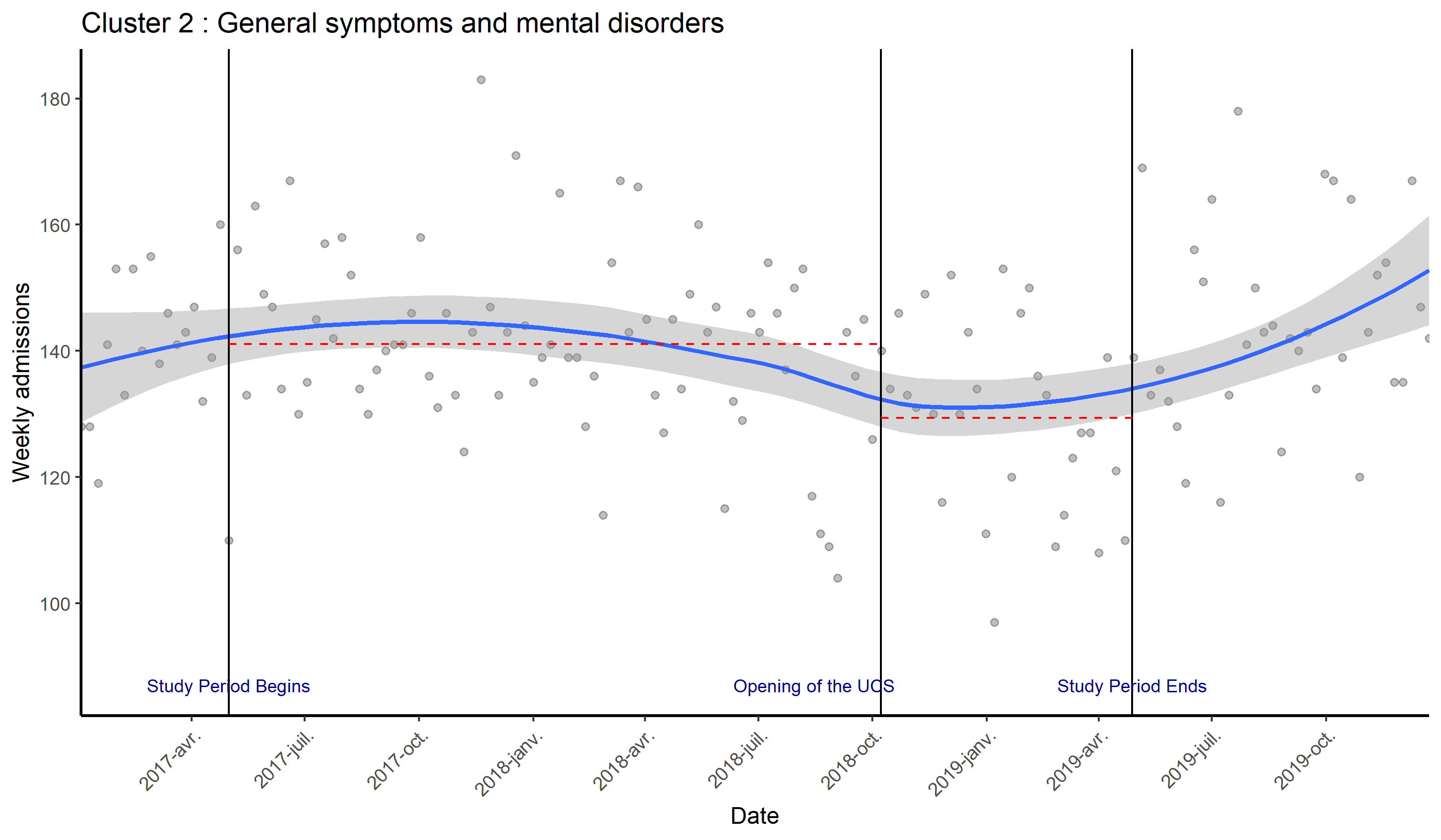 |
| --- | --- |
| **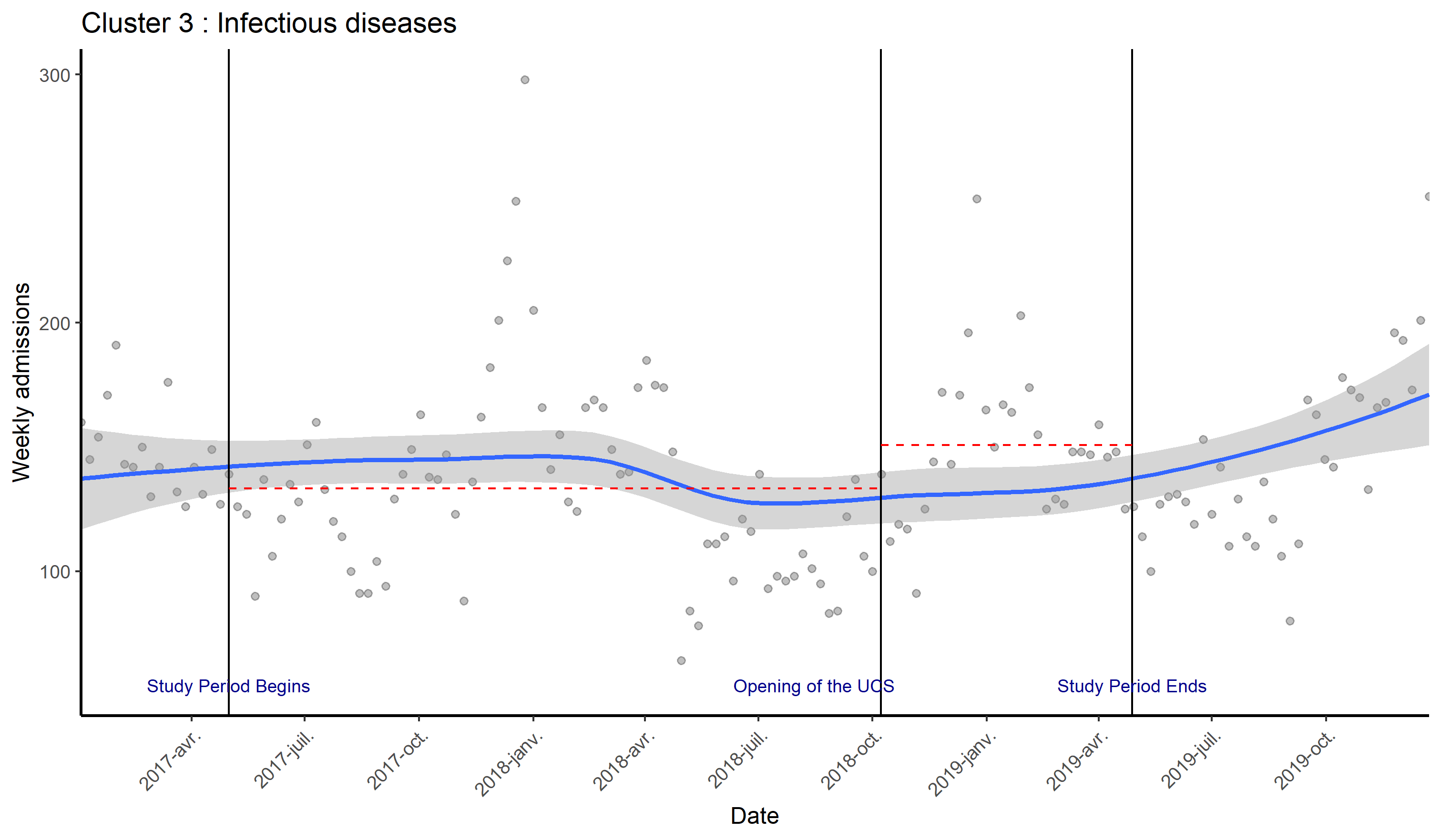** | **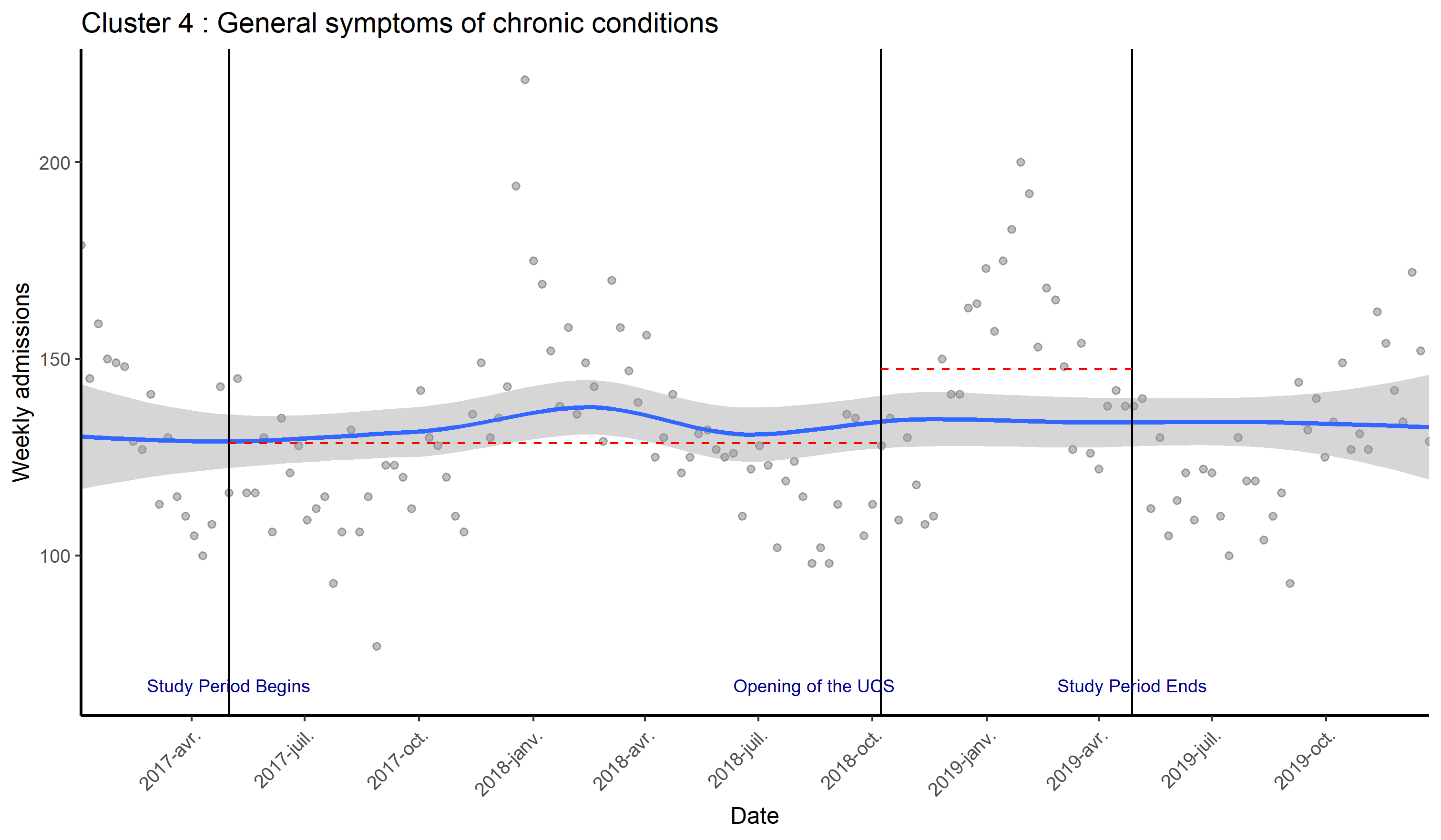** |
| **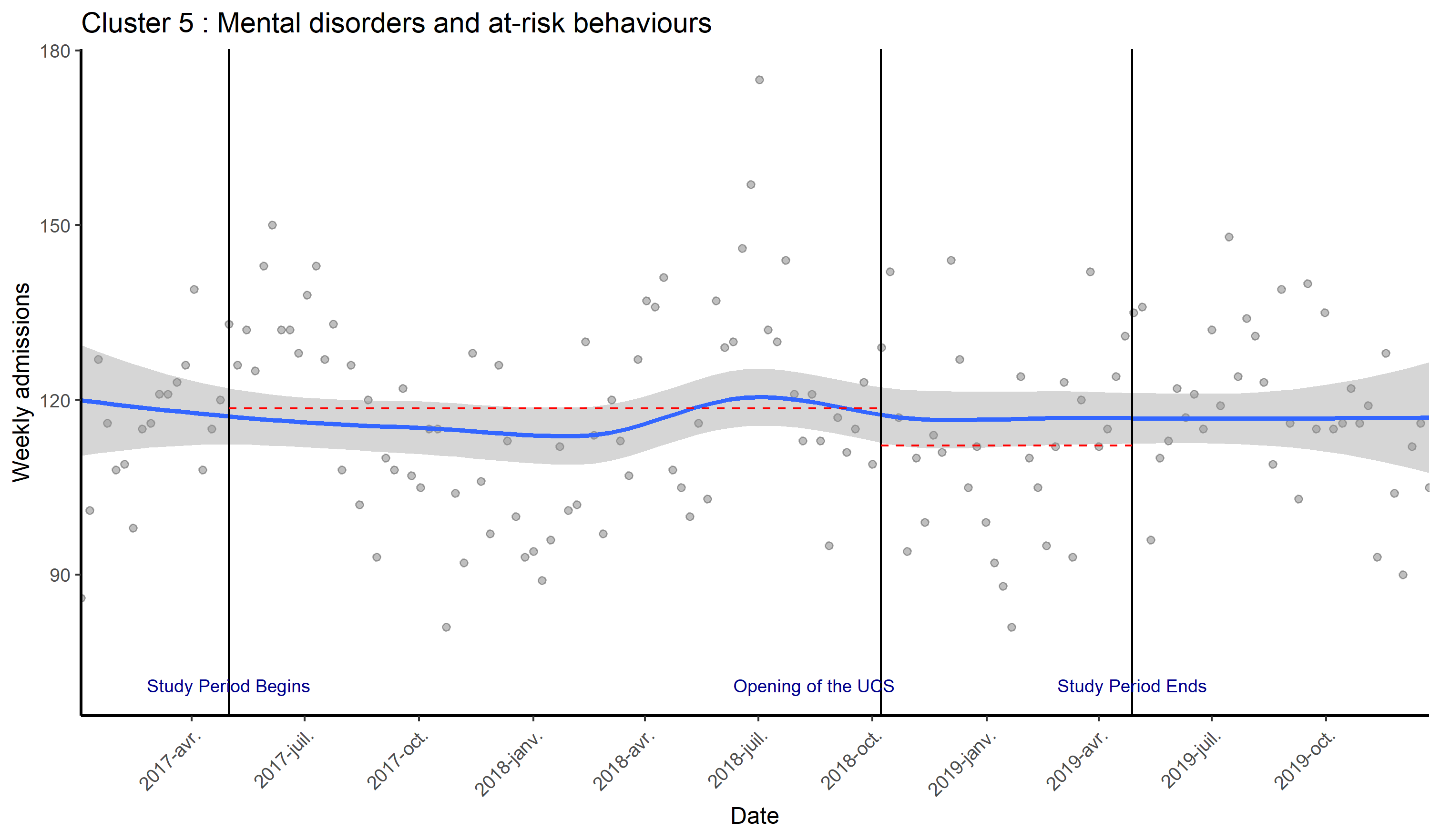** | **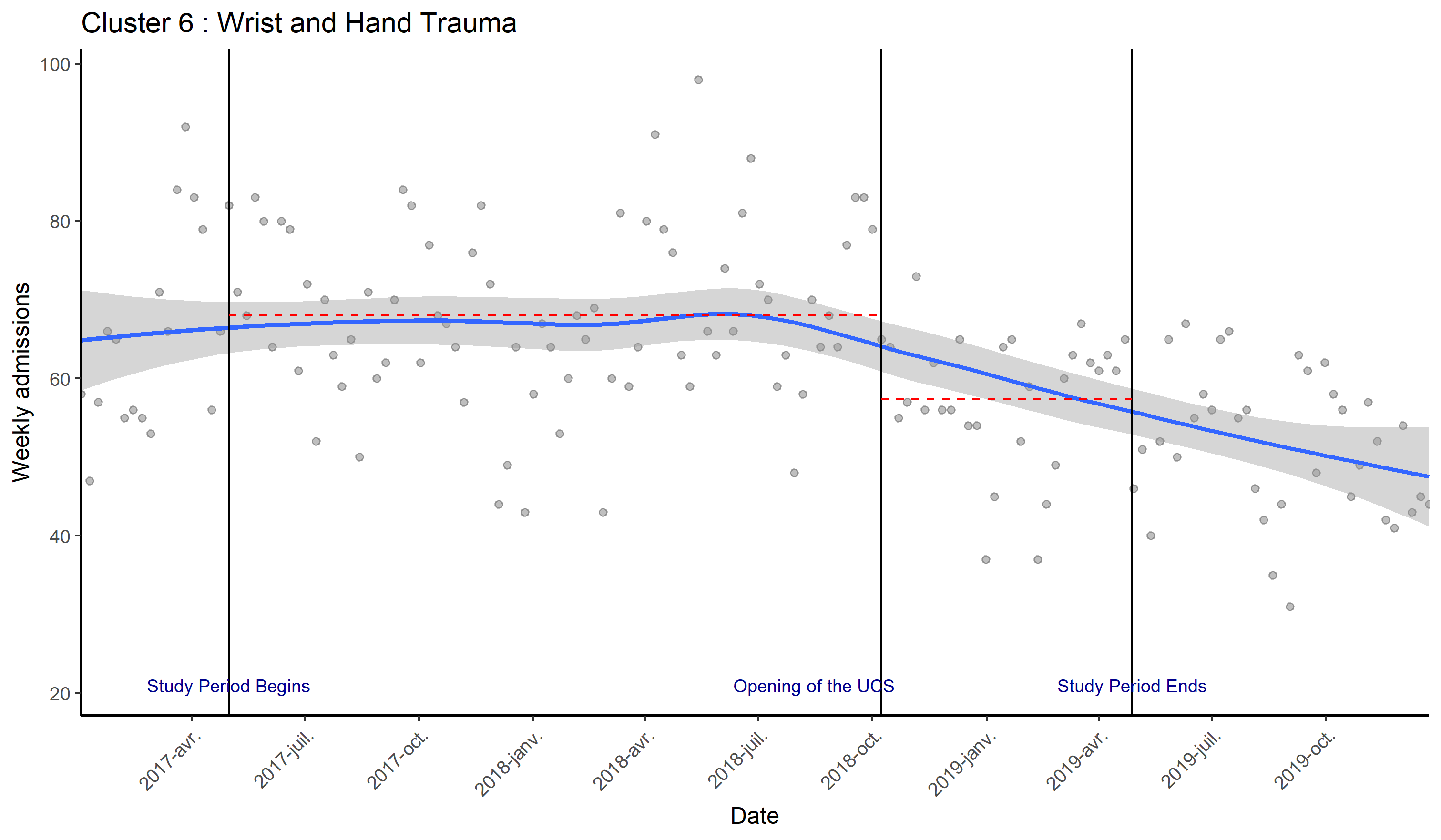** |
| **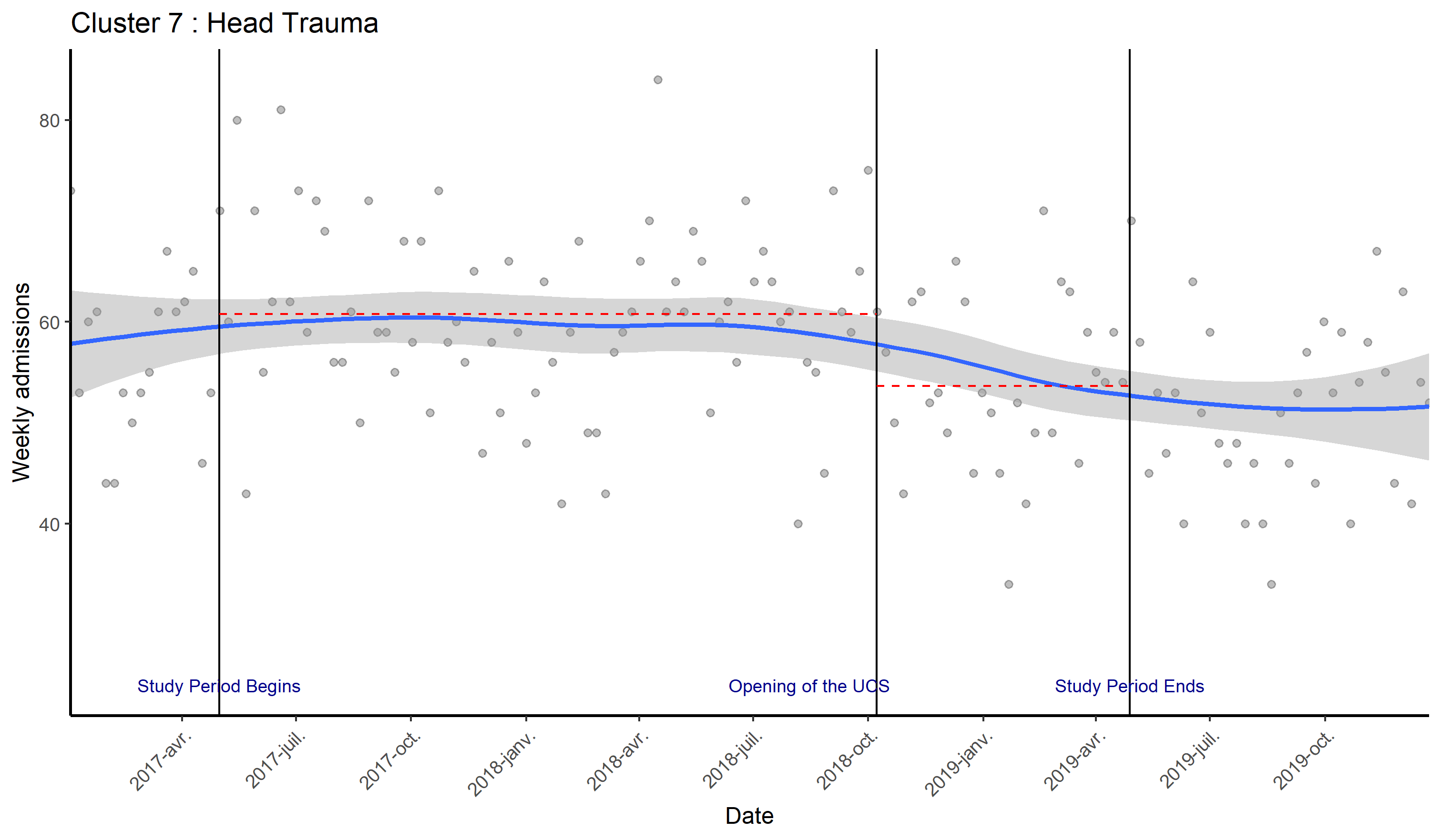** | **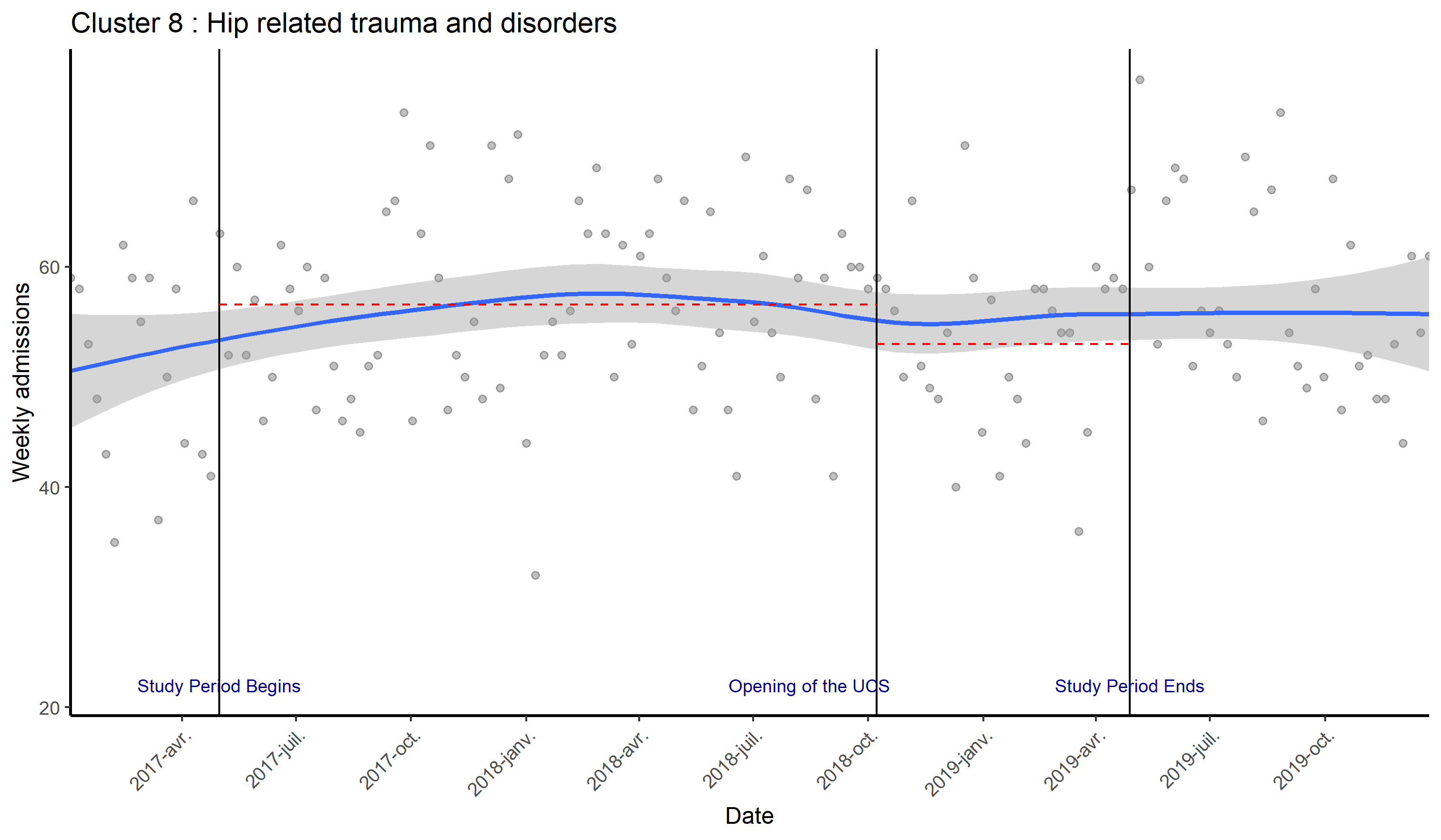** |
| **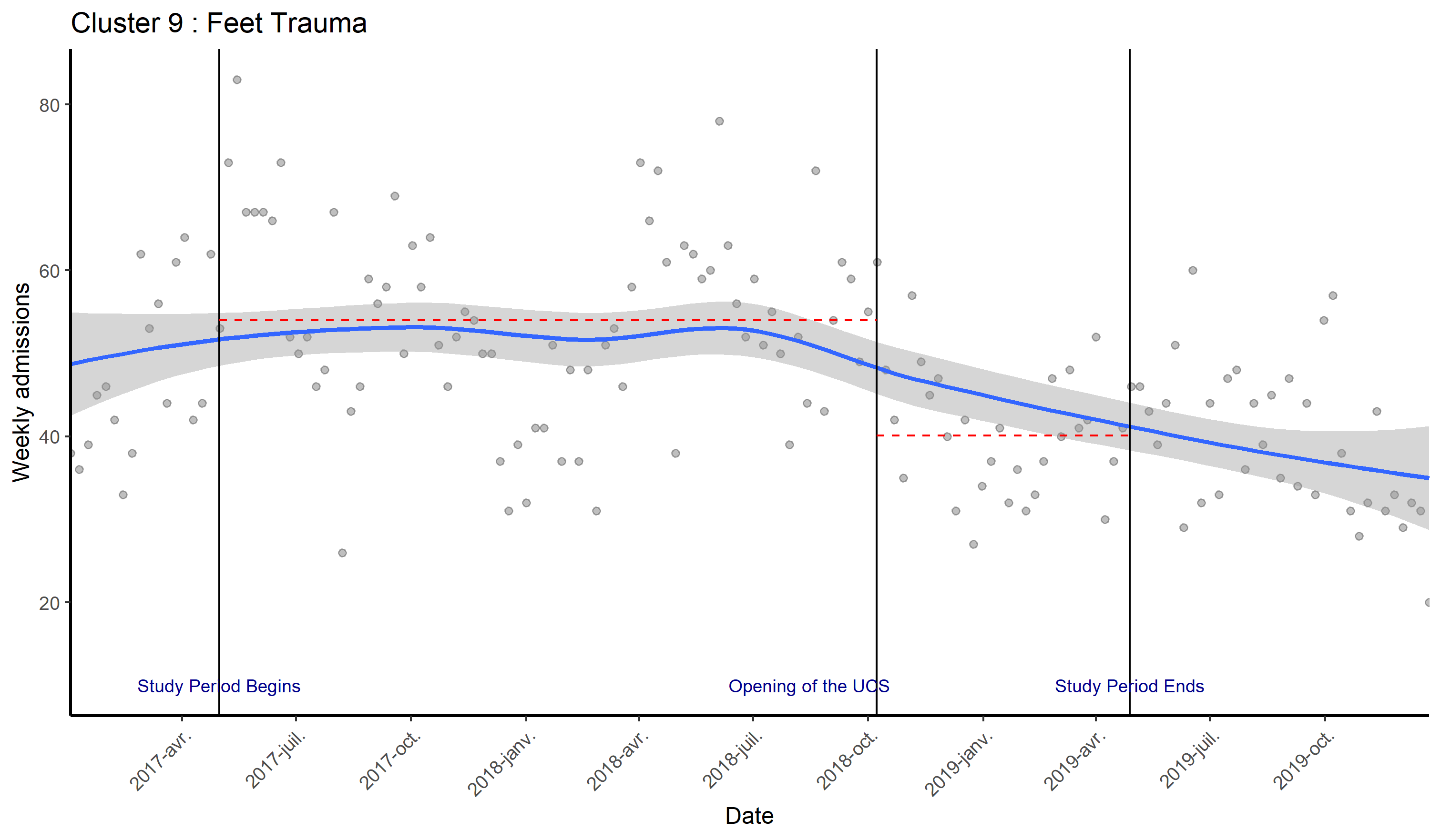** | **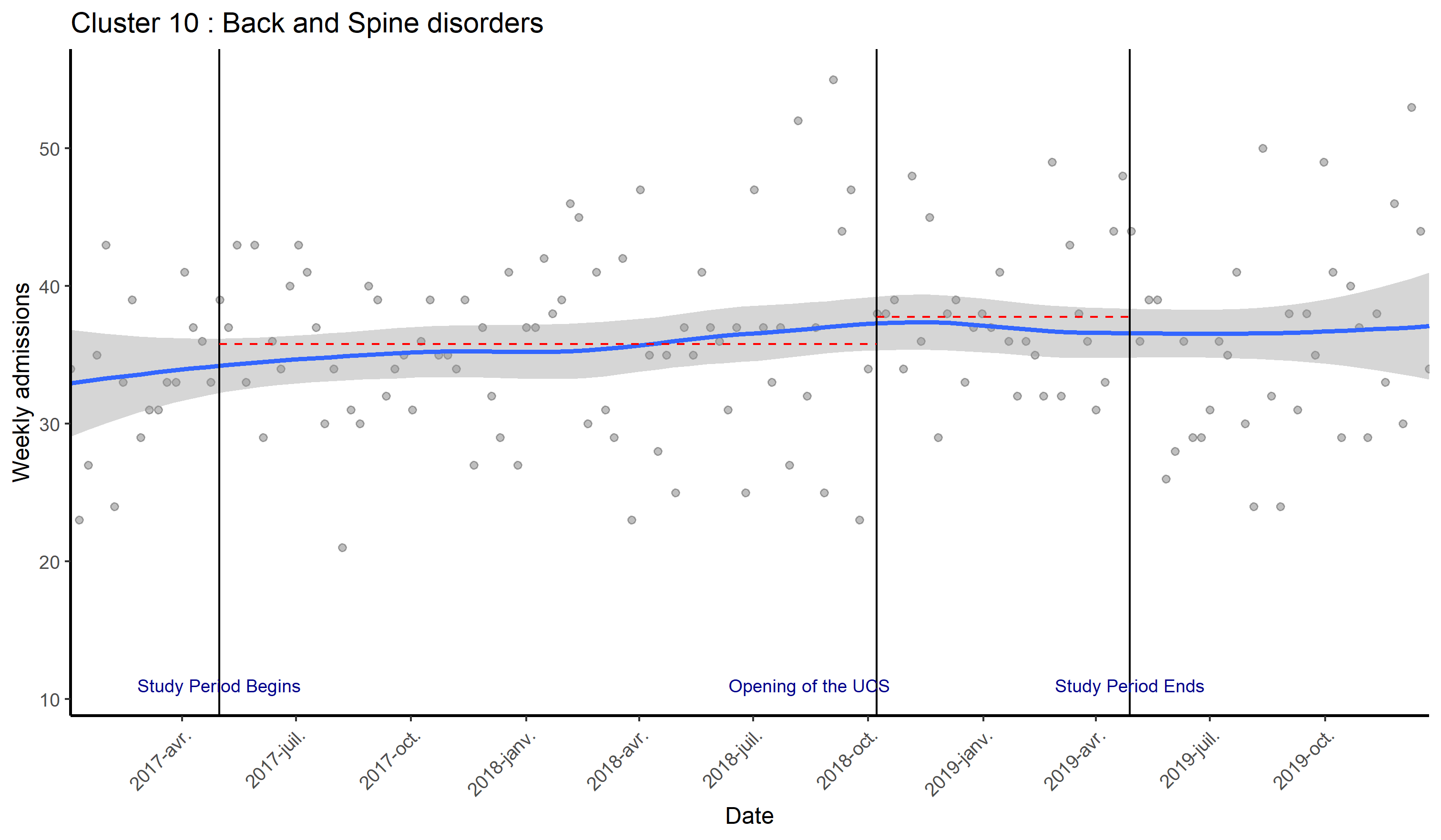** |
| **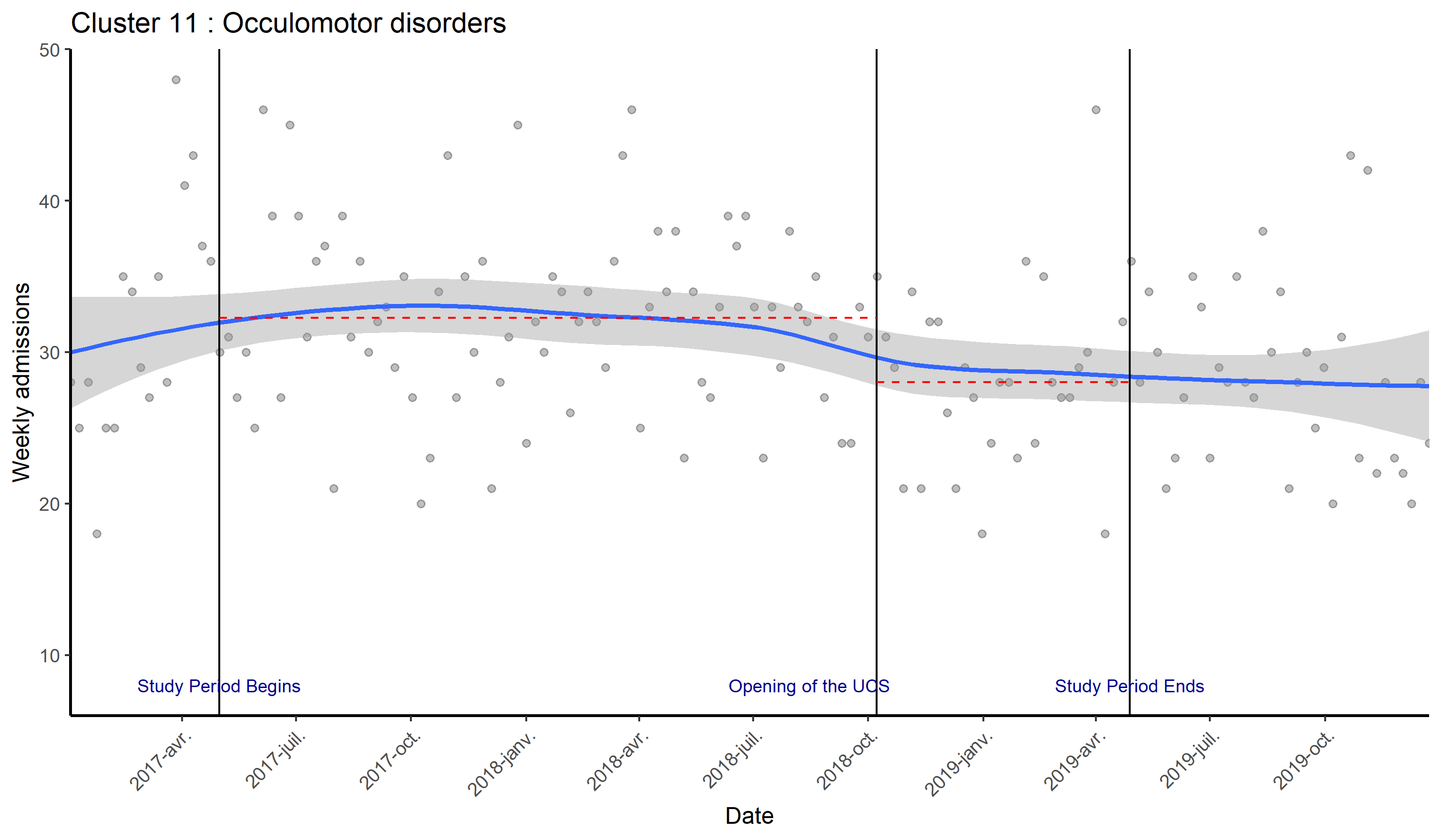** | **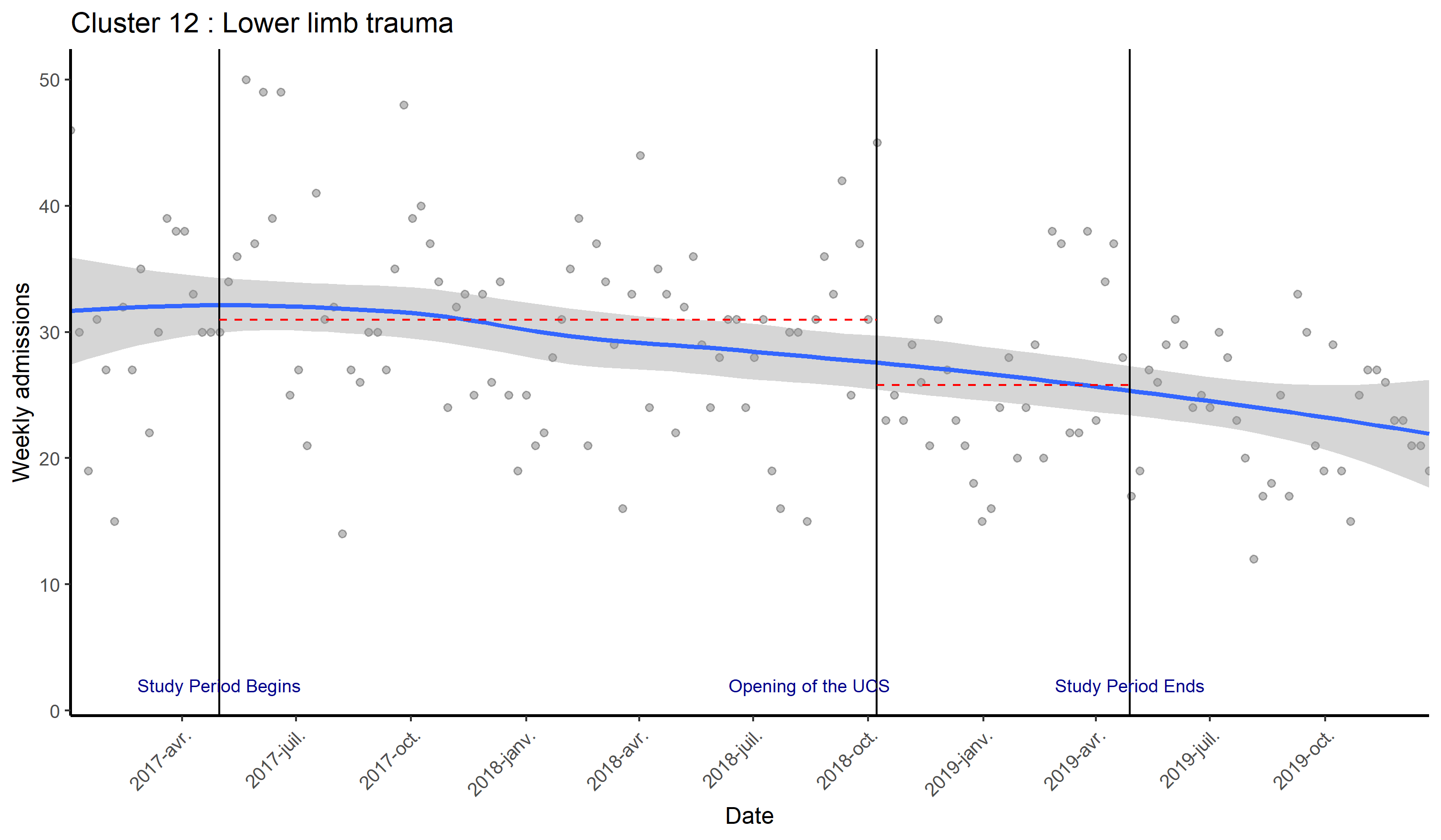** |
| **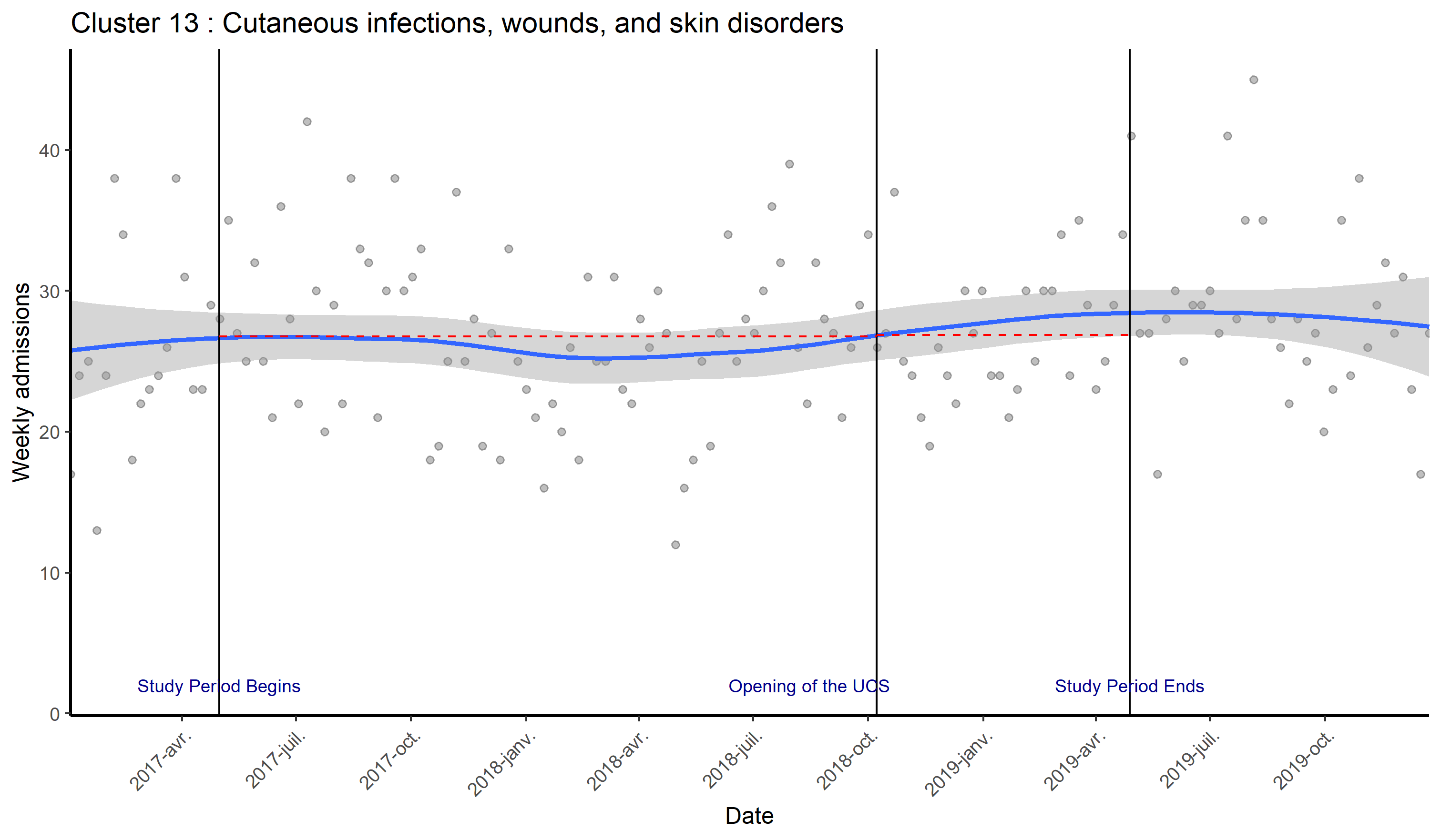** | **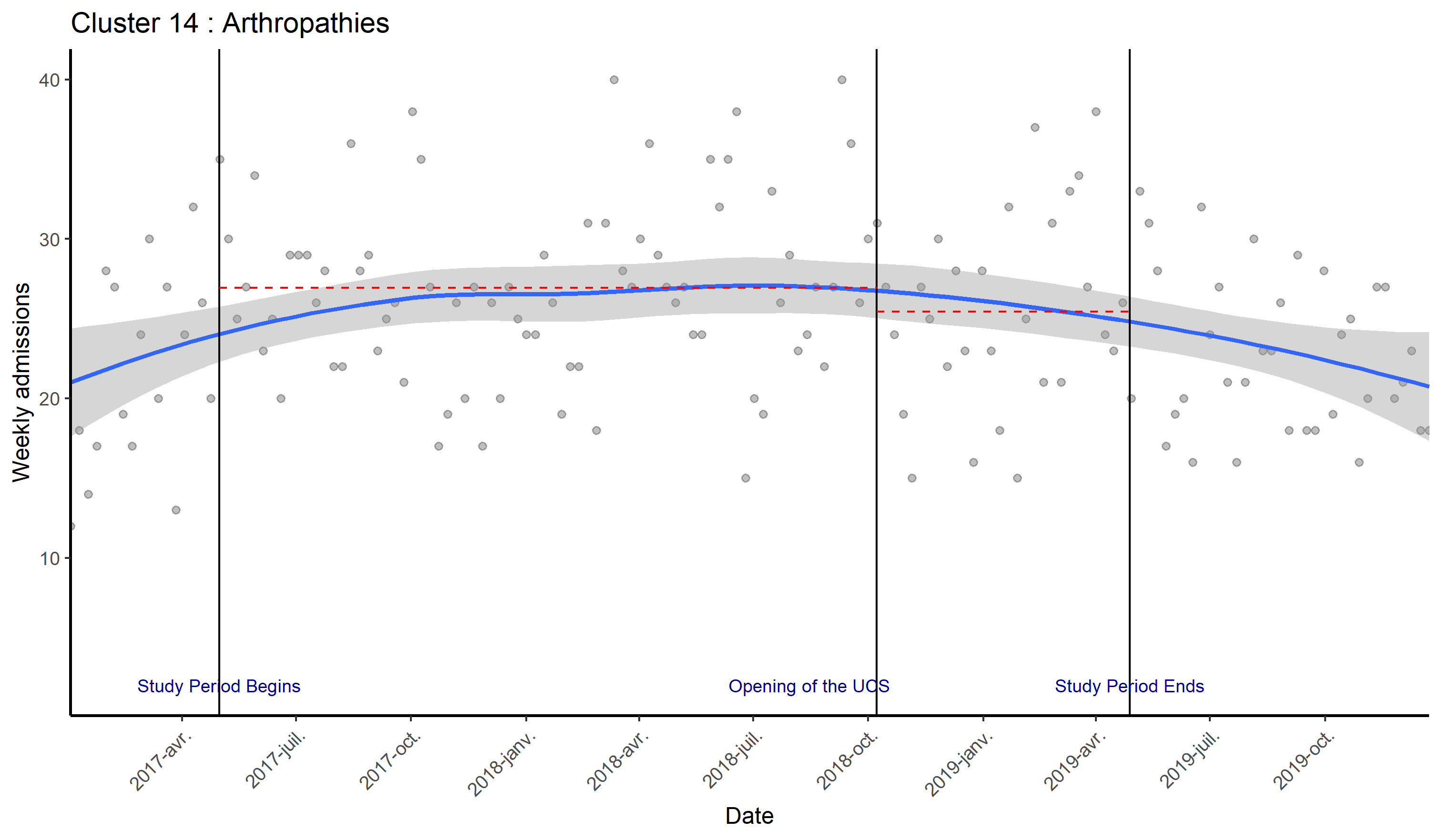** |
| **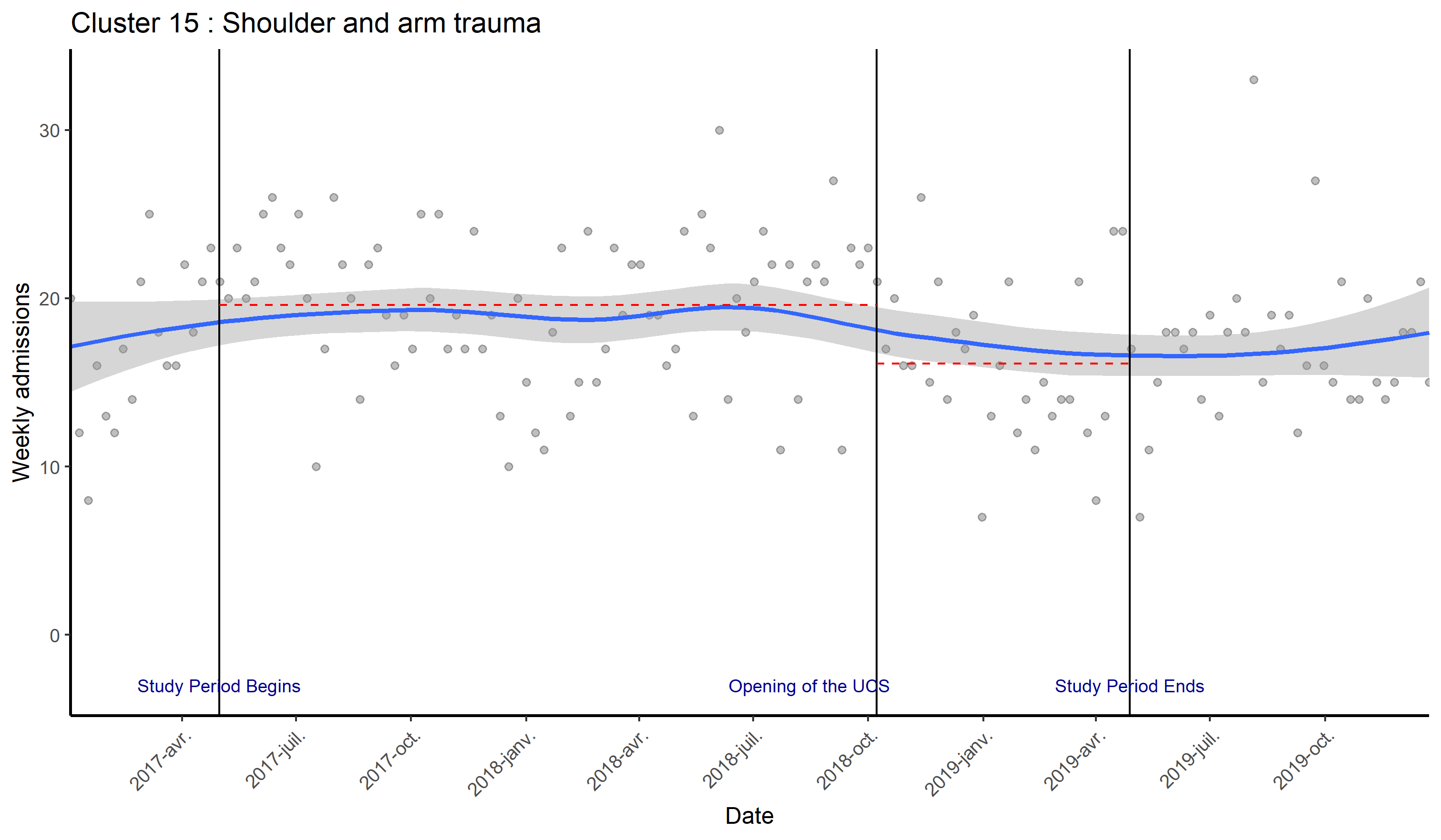** | **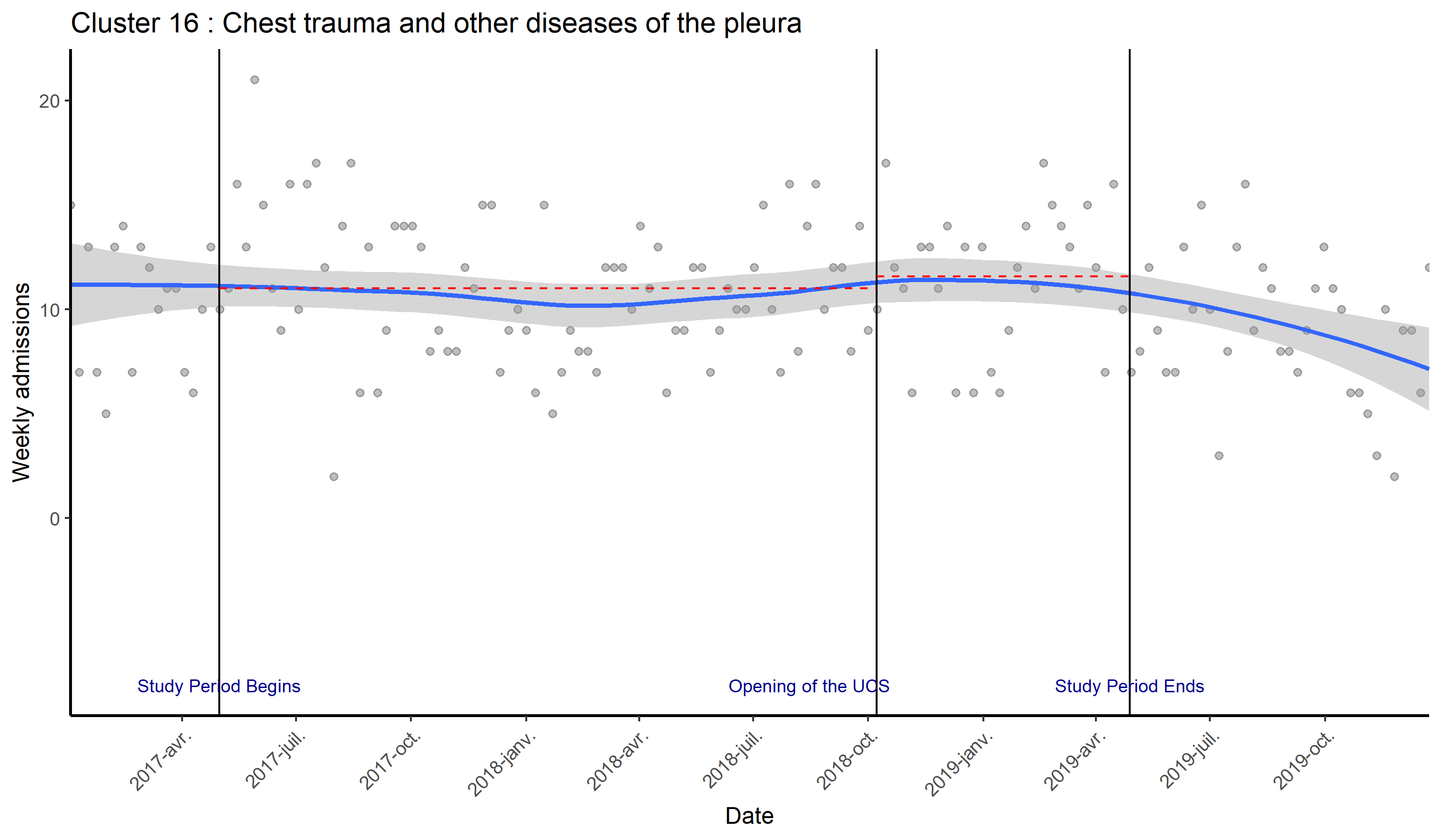** |

**With the 21 ICD 10 chapters**

| **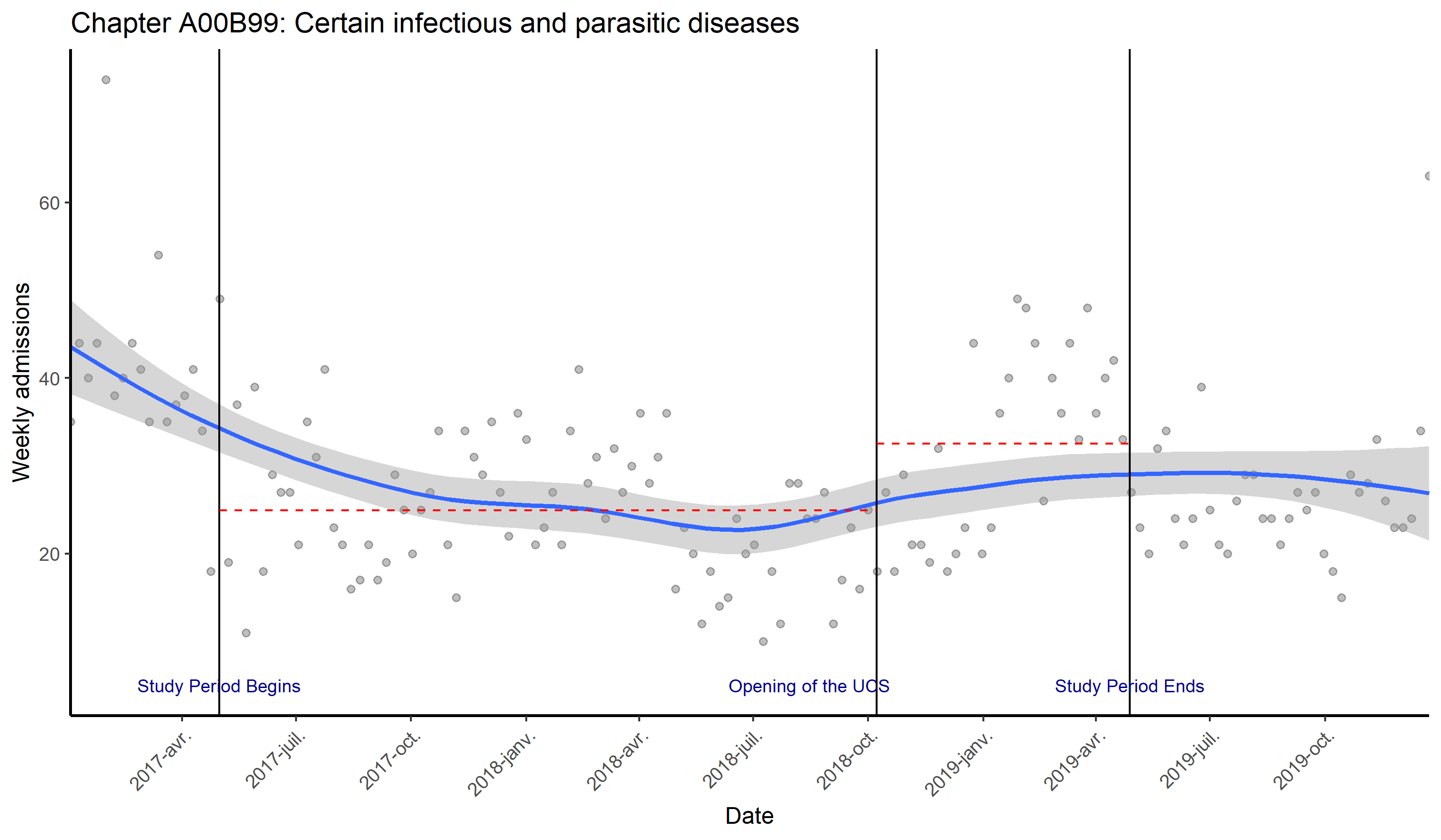** | **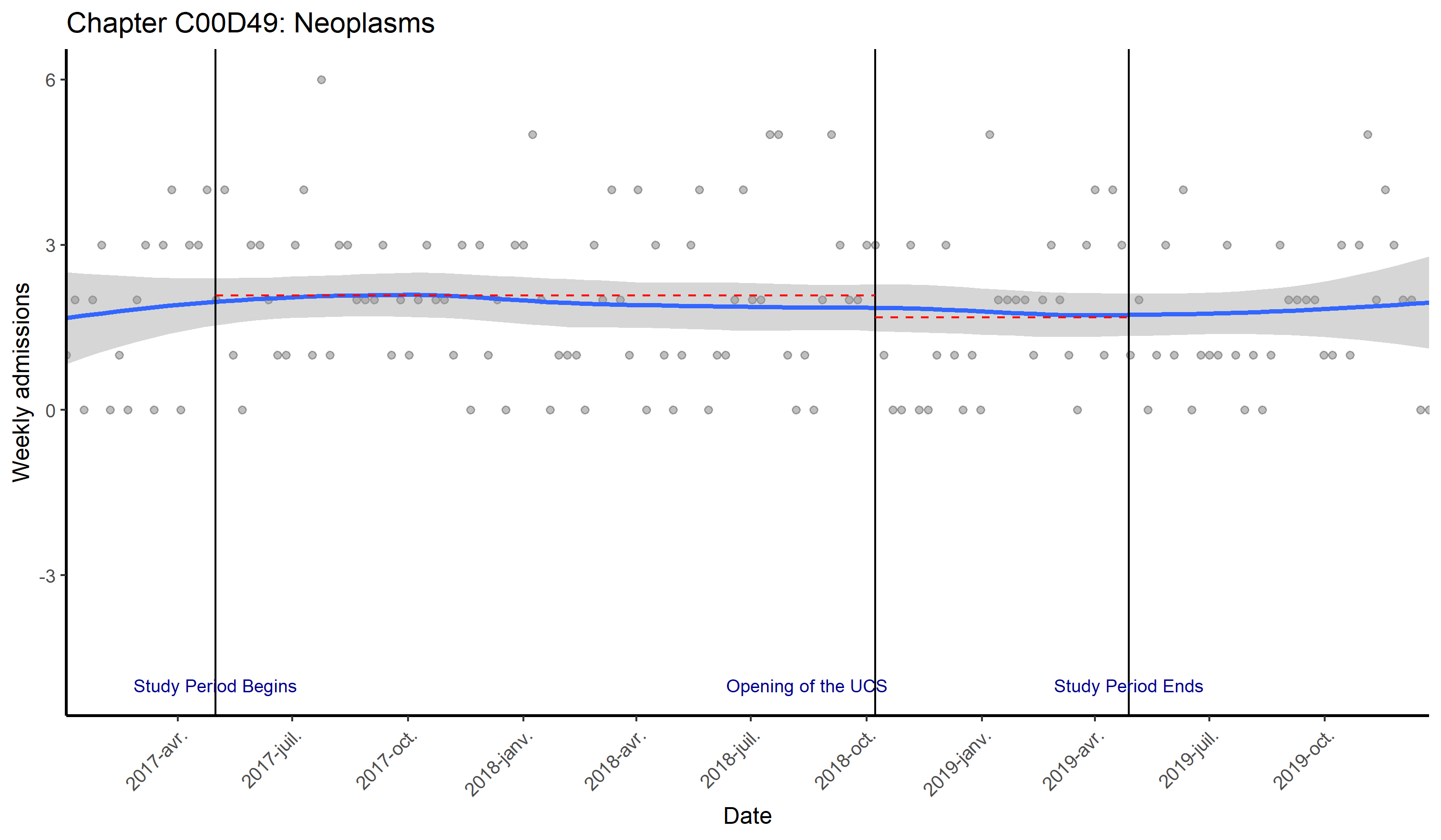** |
| --- | --- |
| **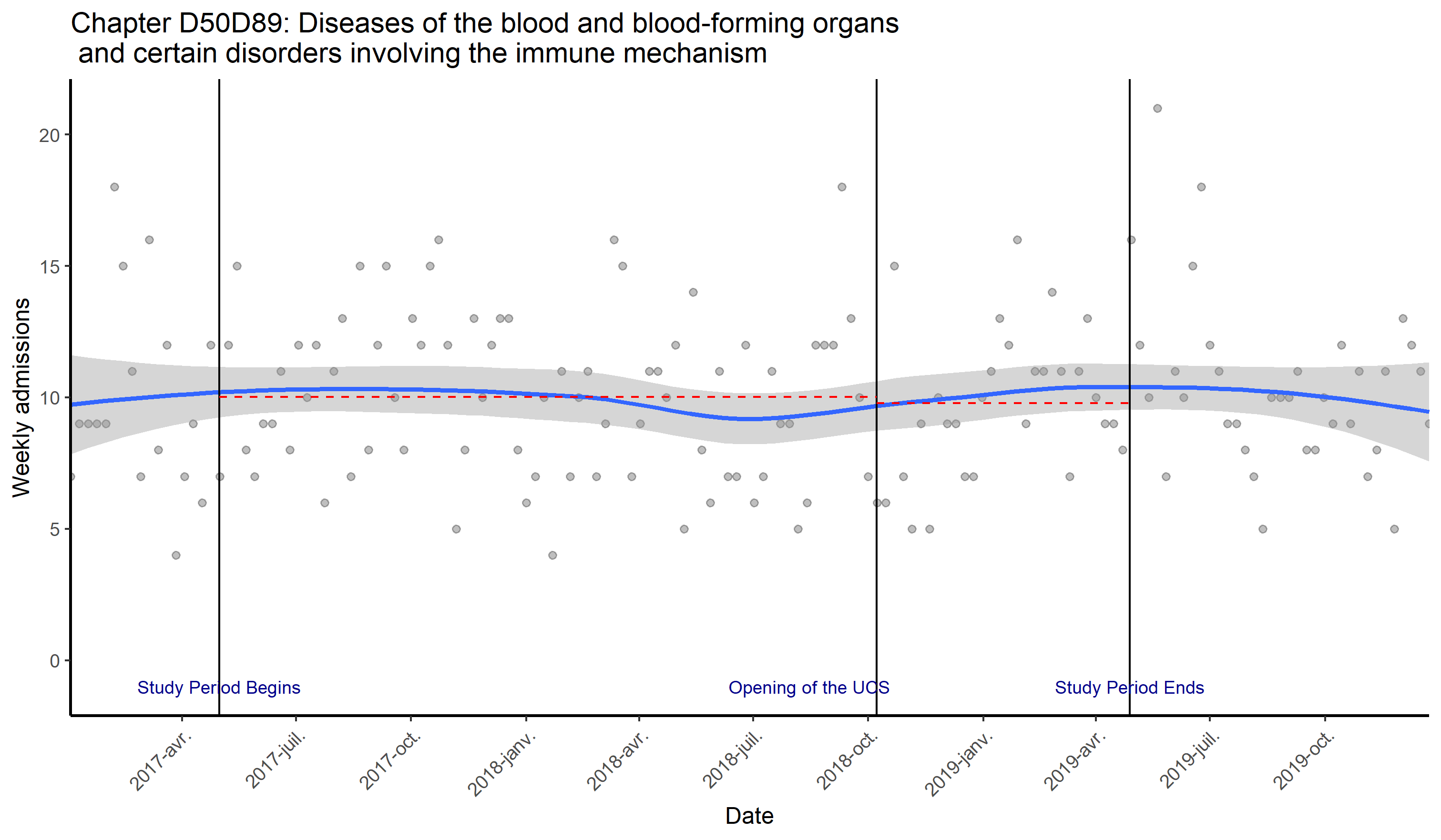** | **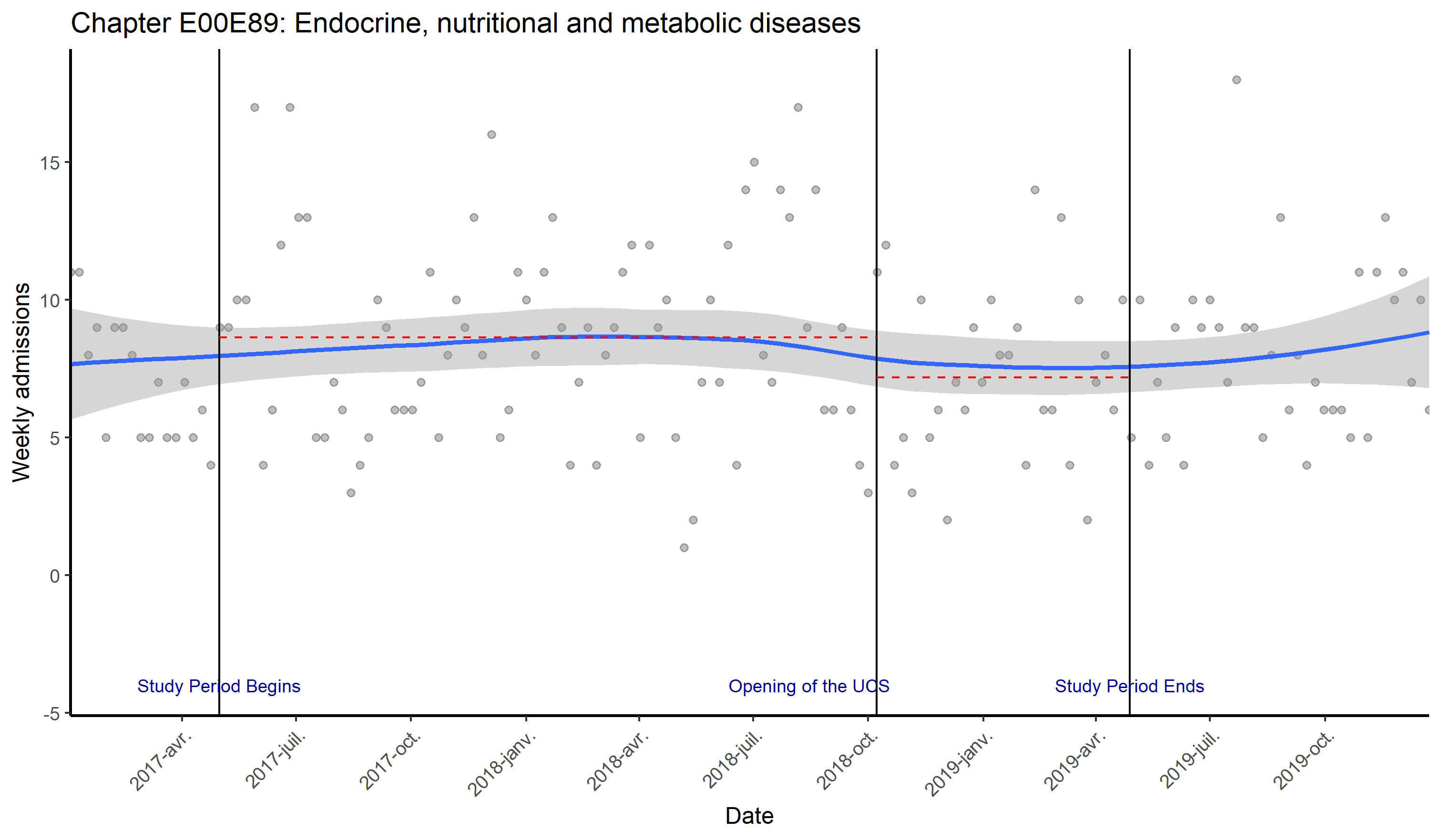** |
| **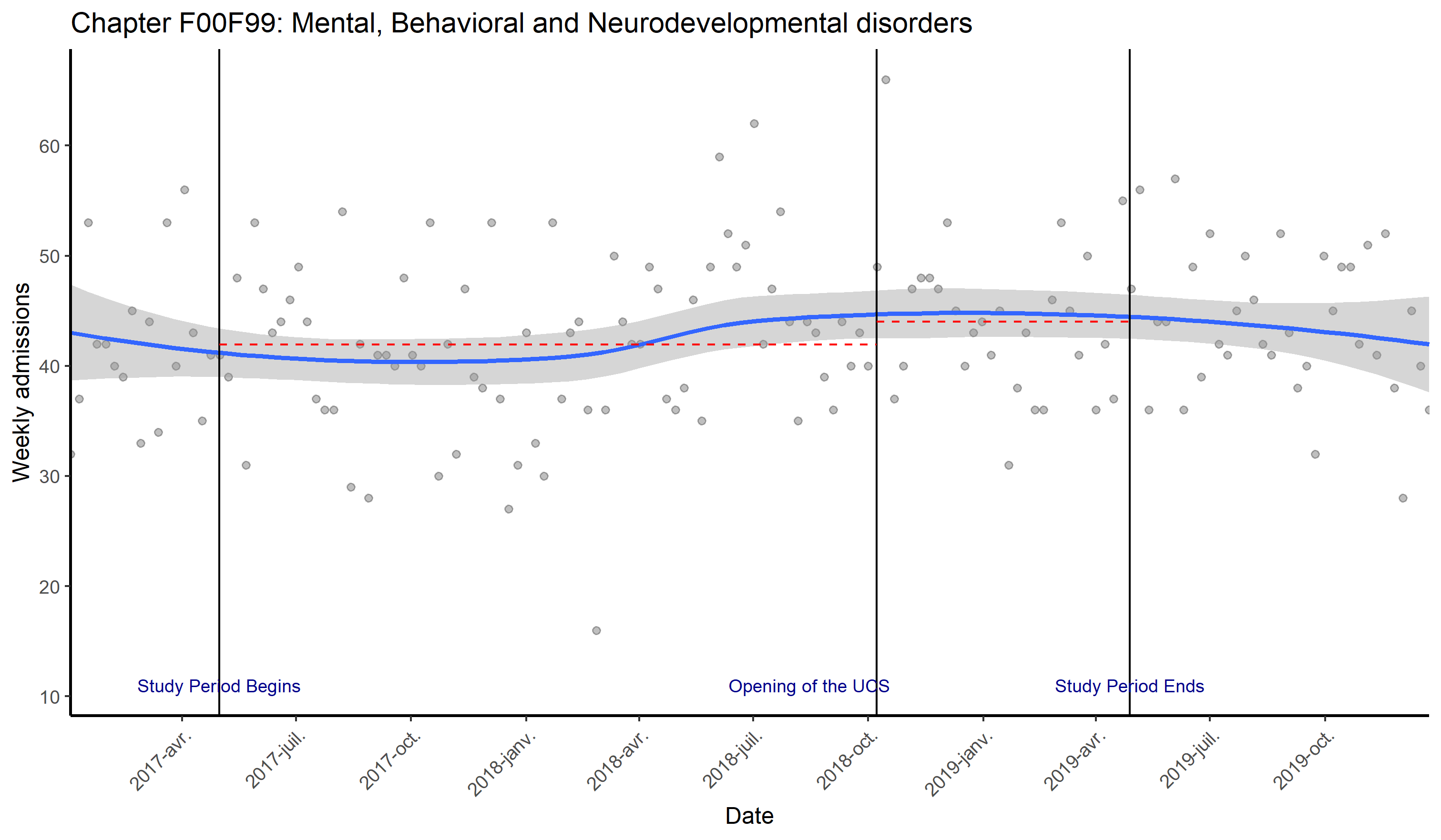** | **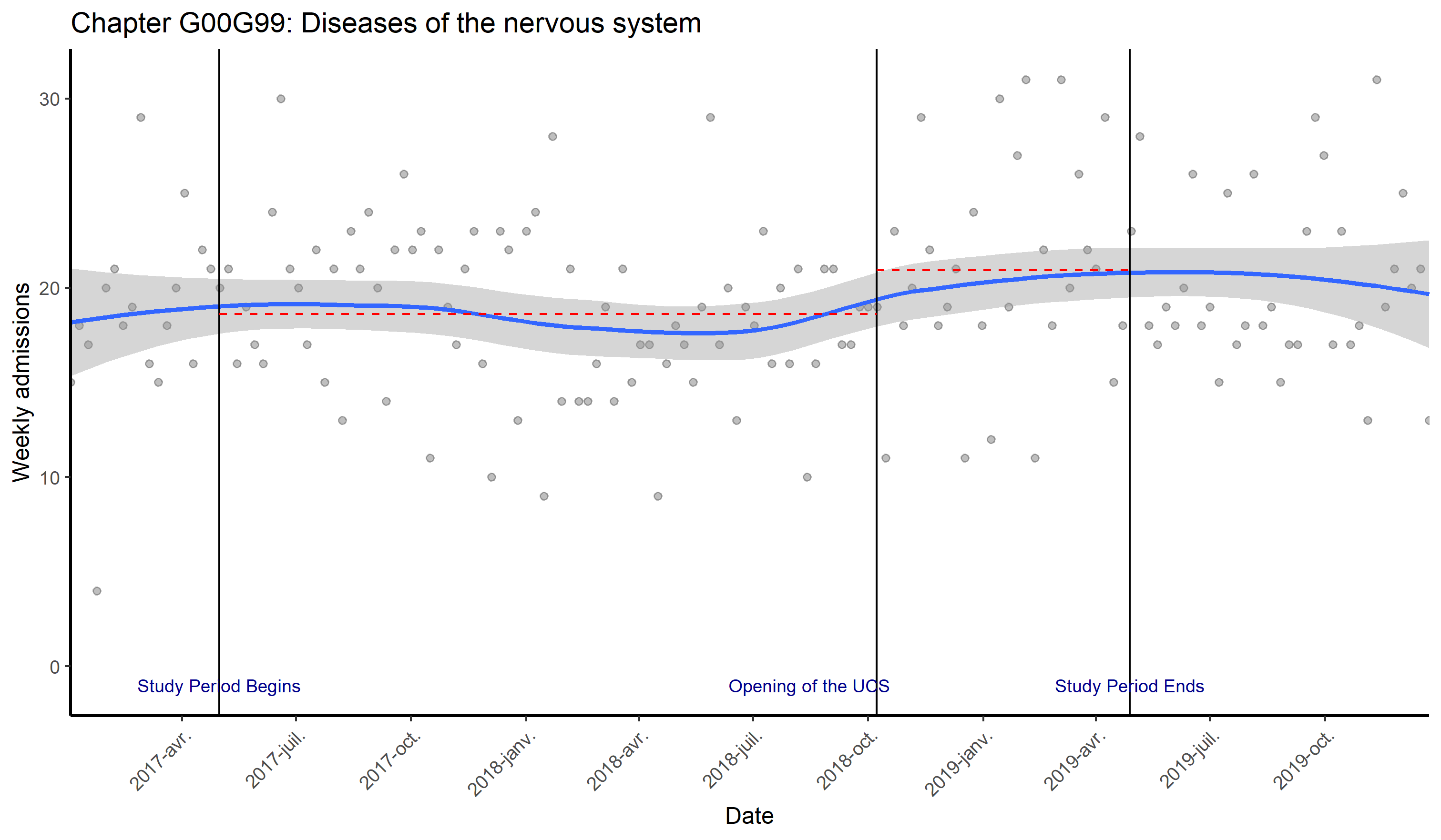** |
| **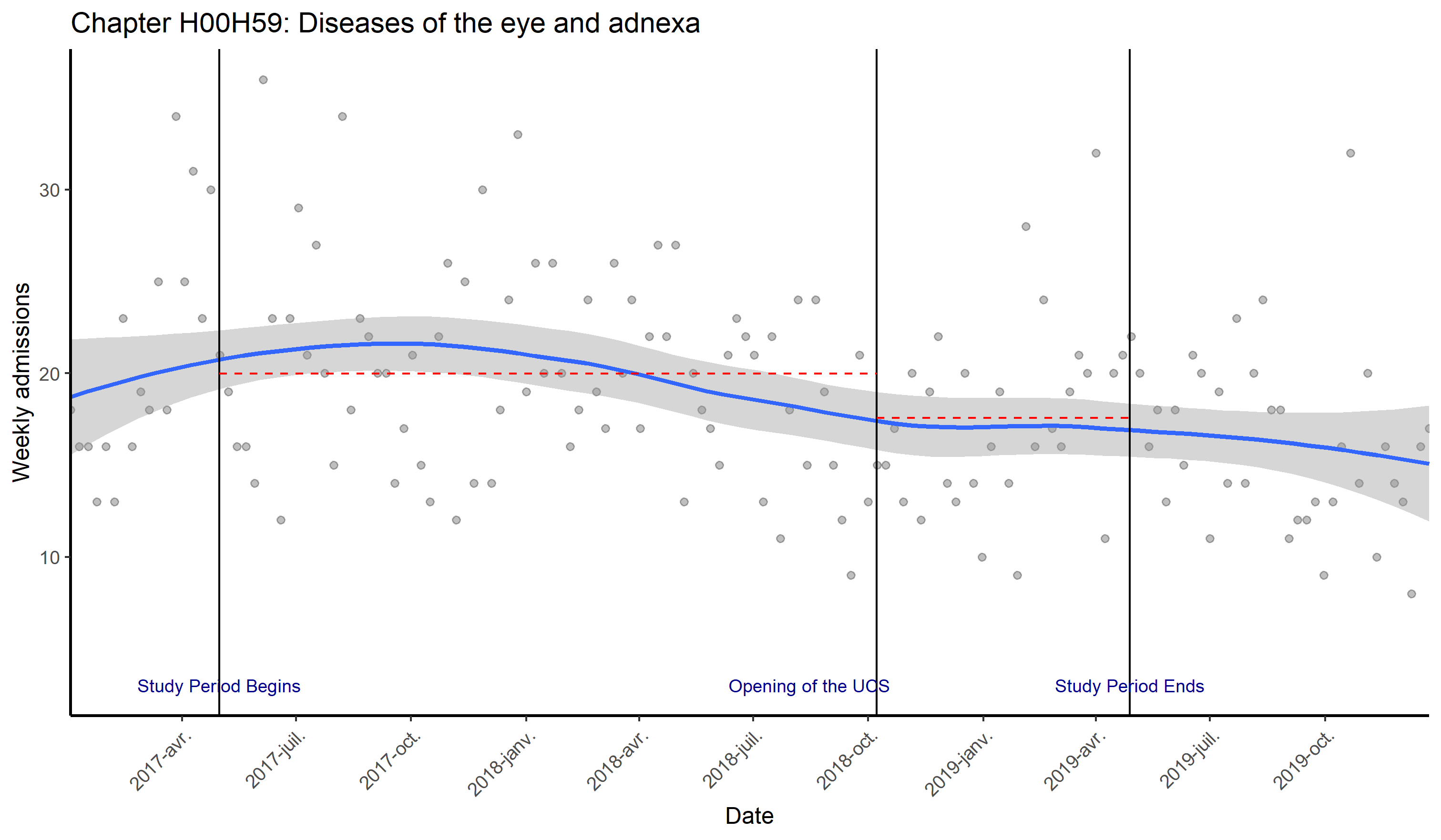** | **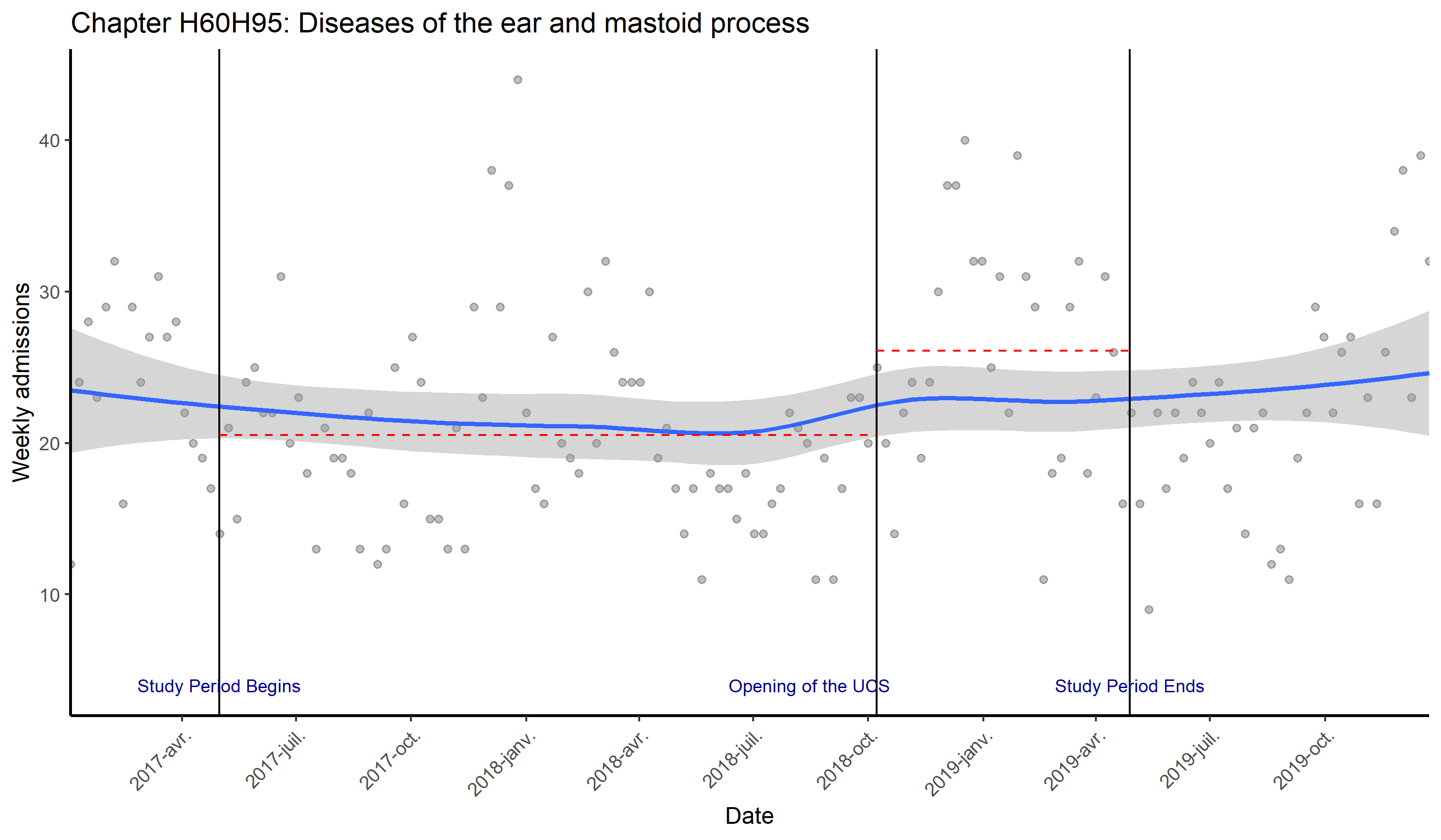** |
| **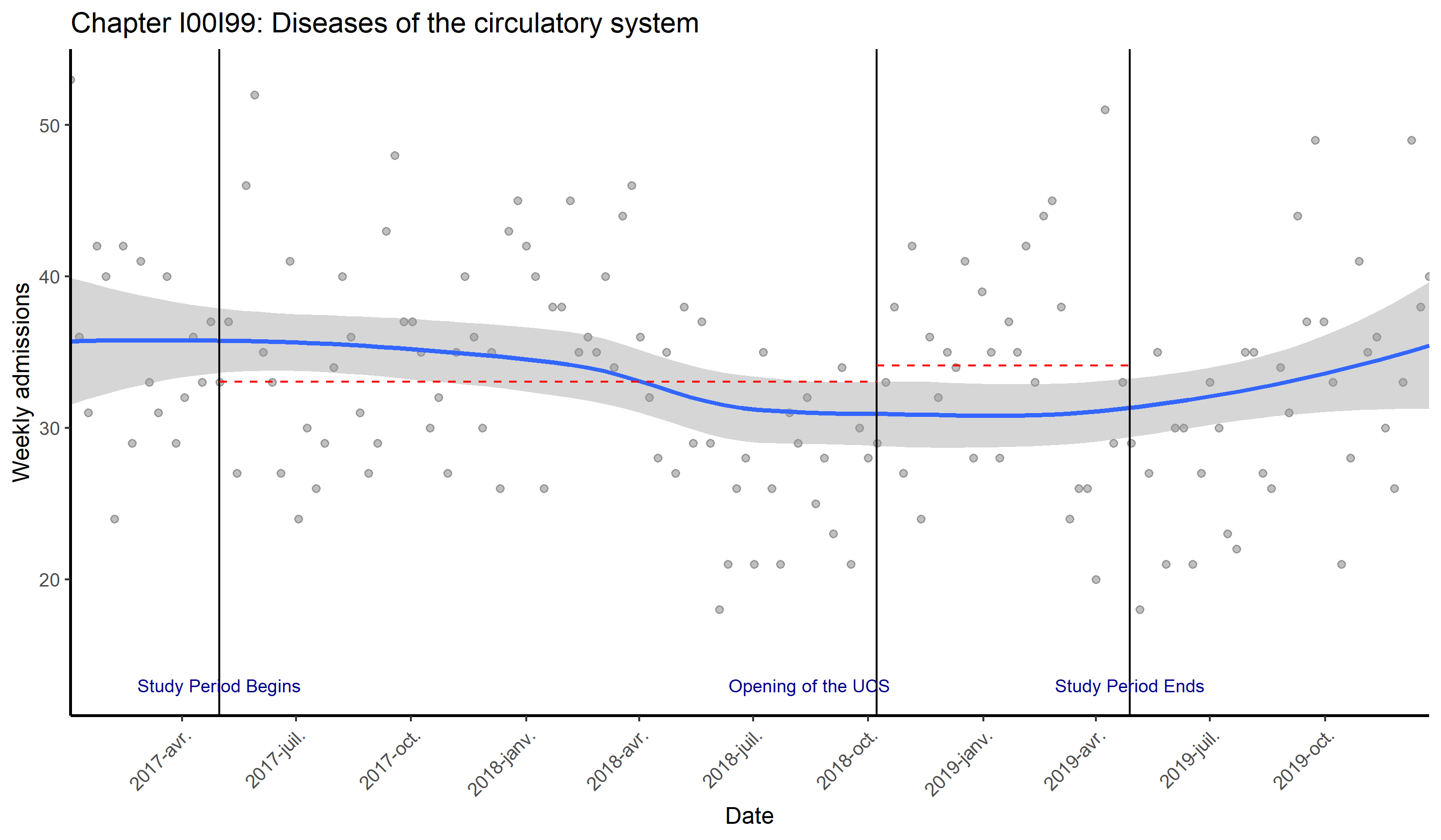** | **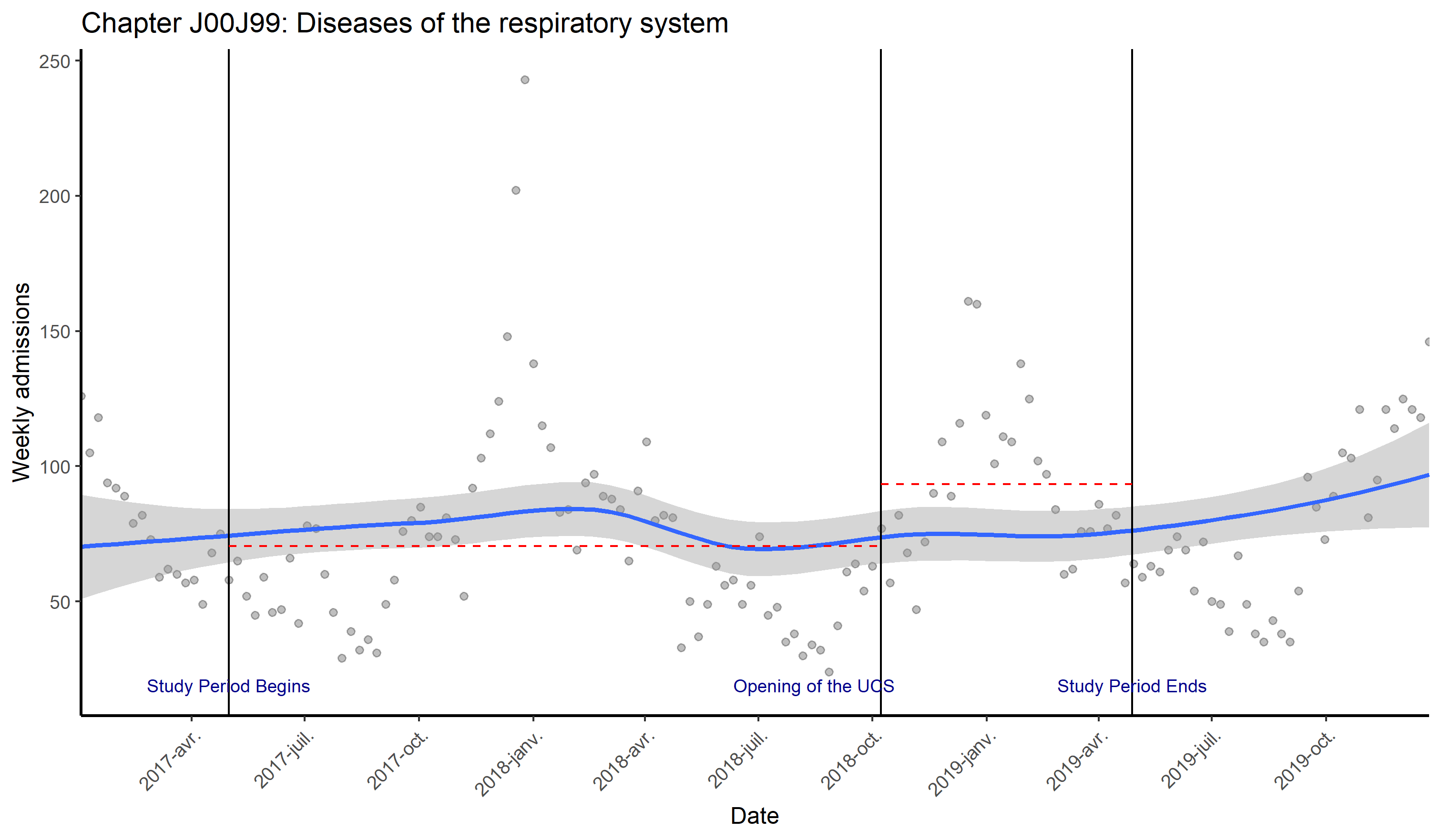** |
| **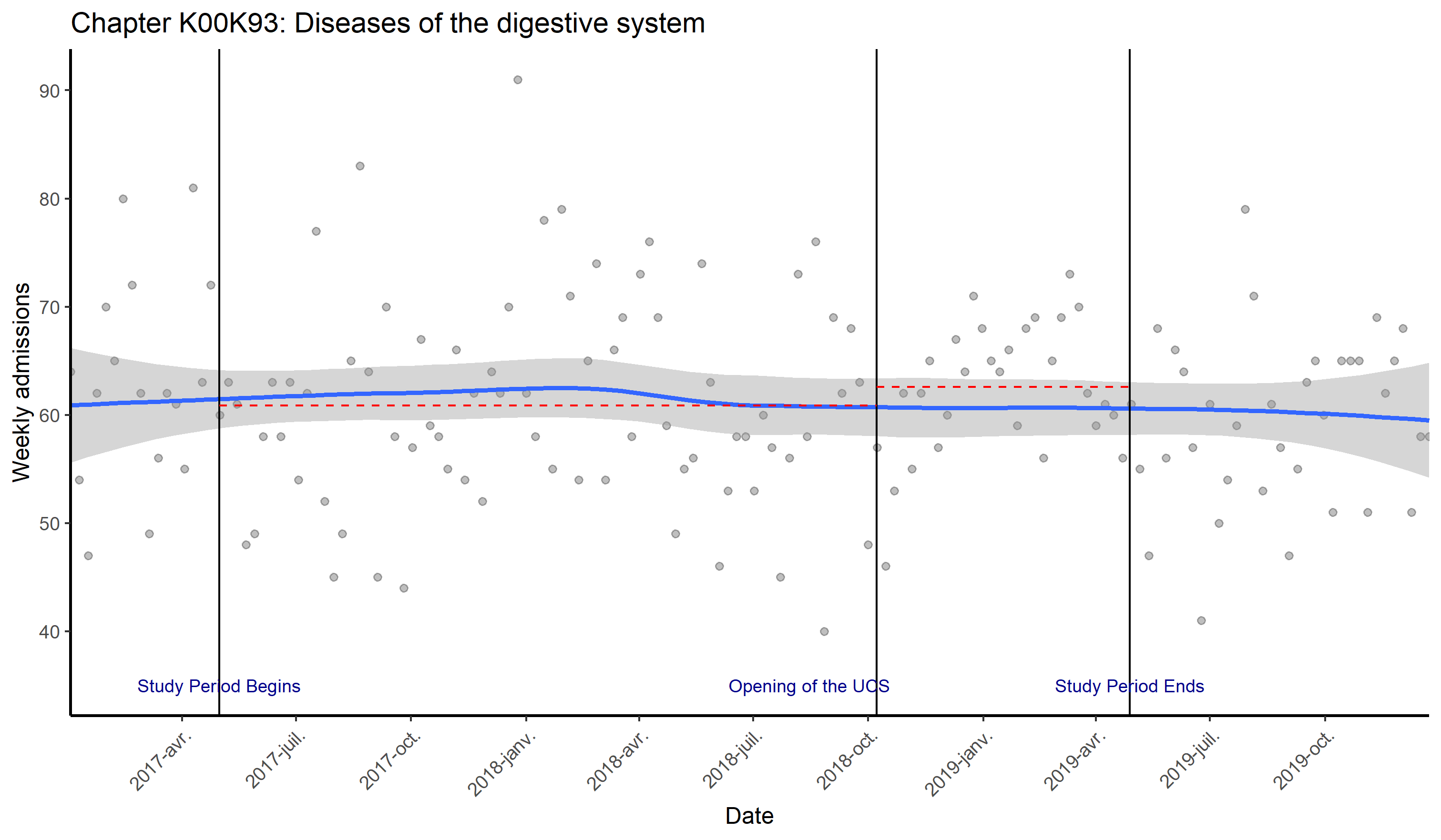** | **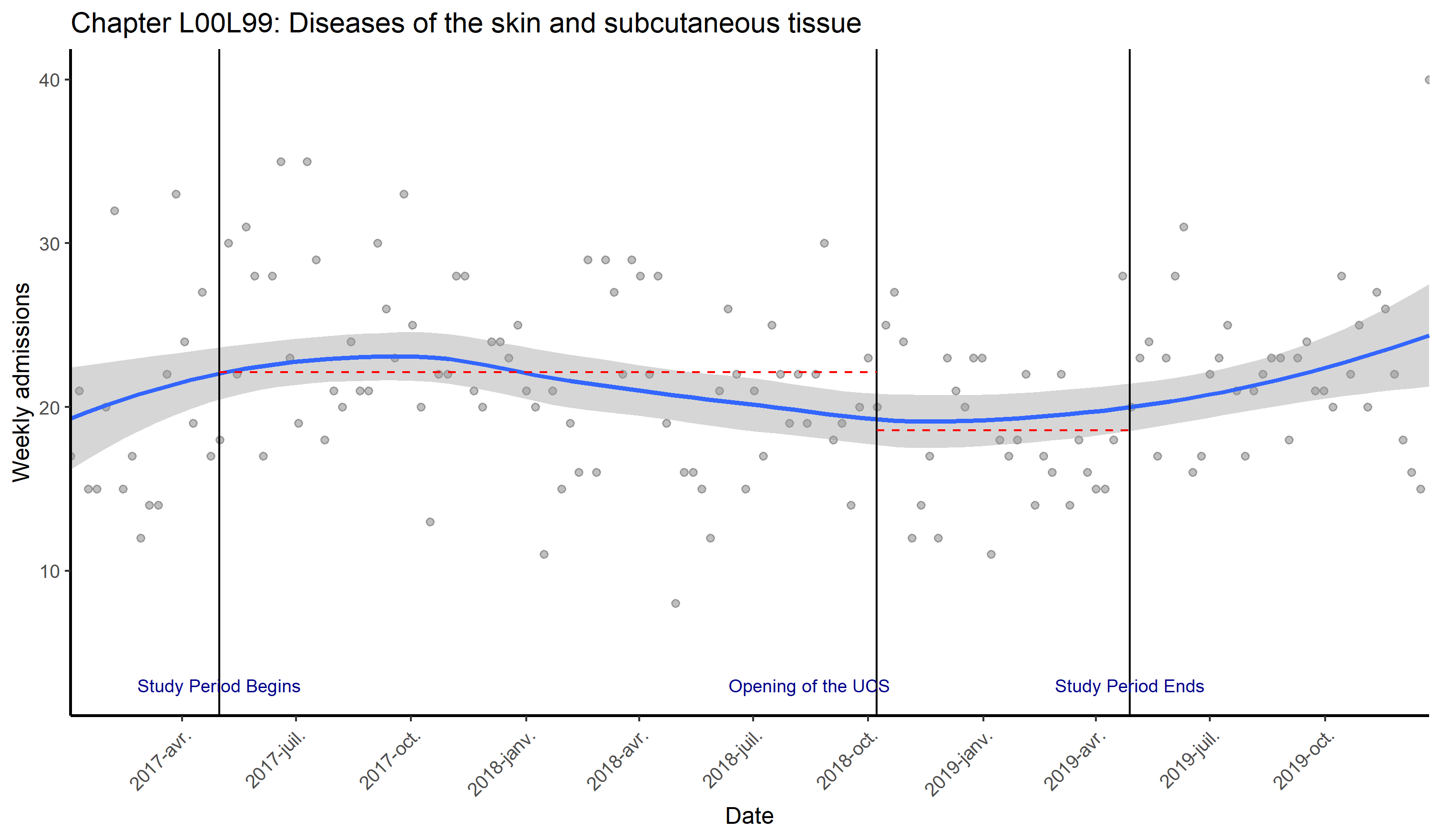** |
| **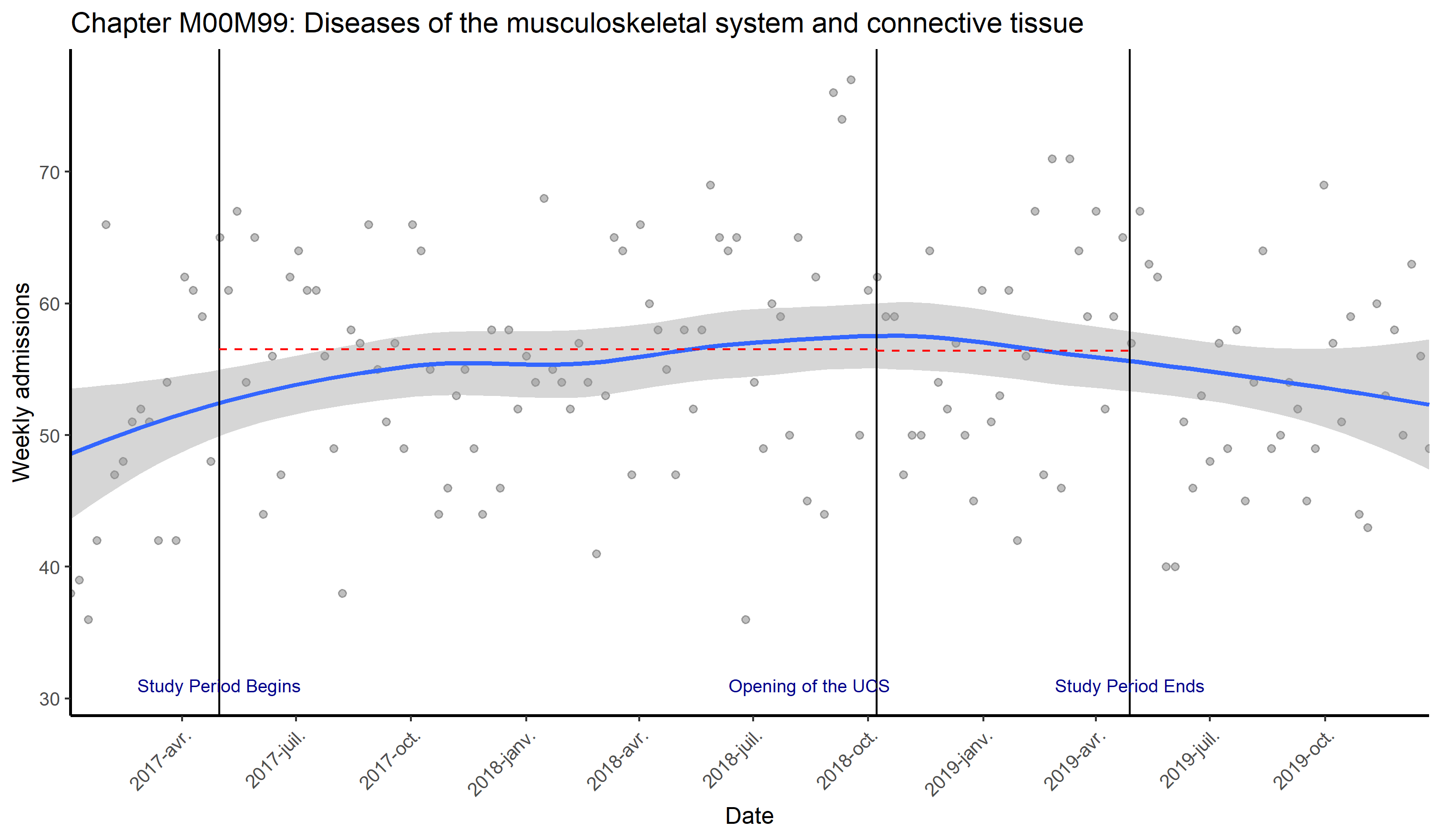** | **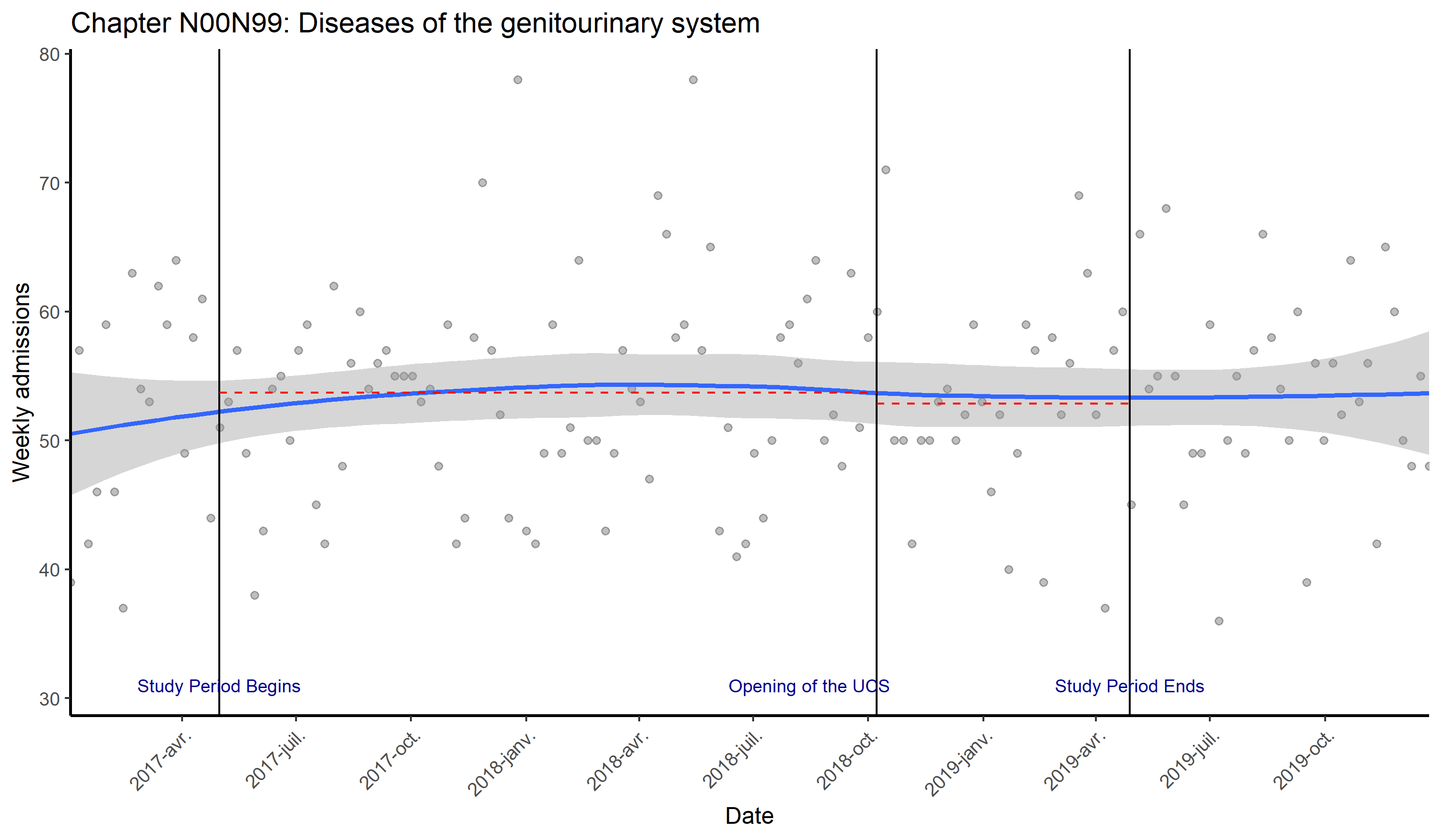** |
| **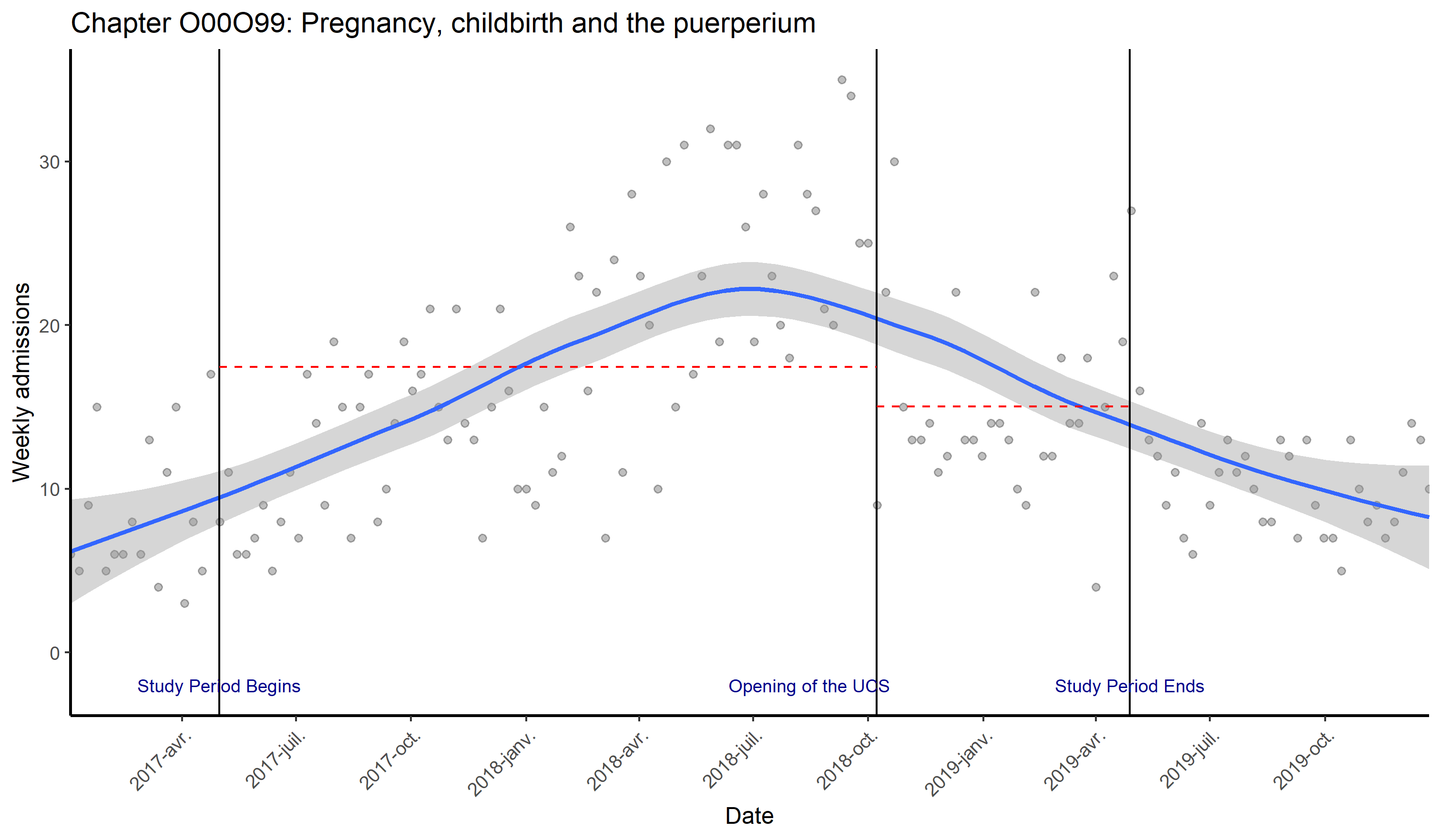** | **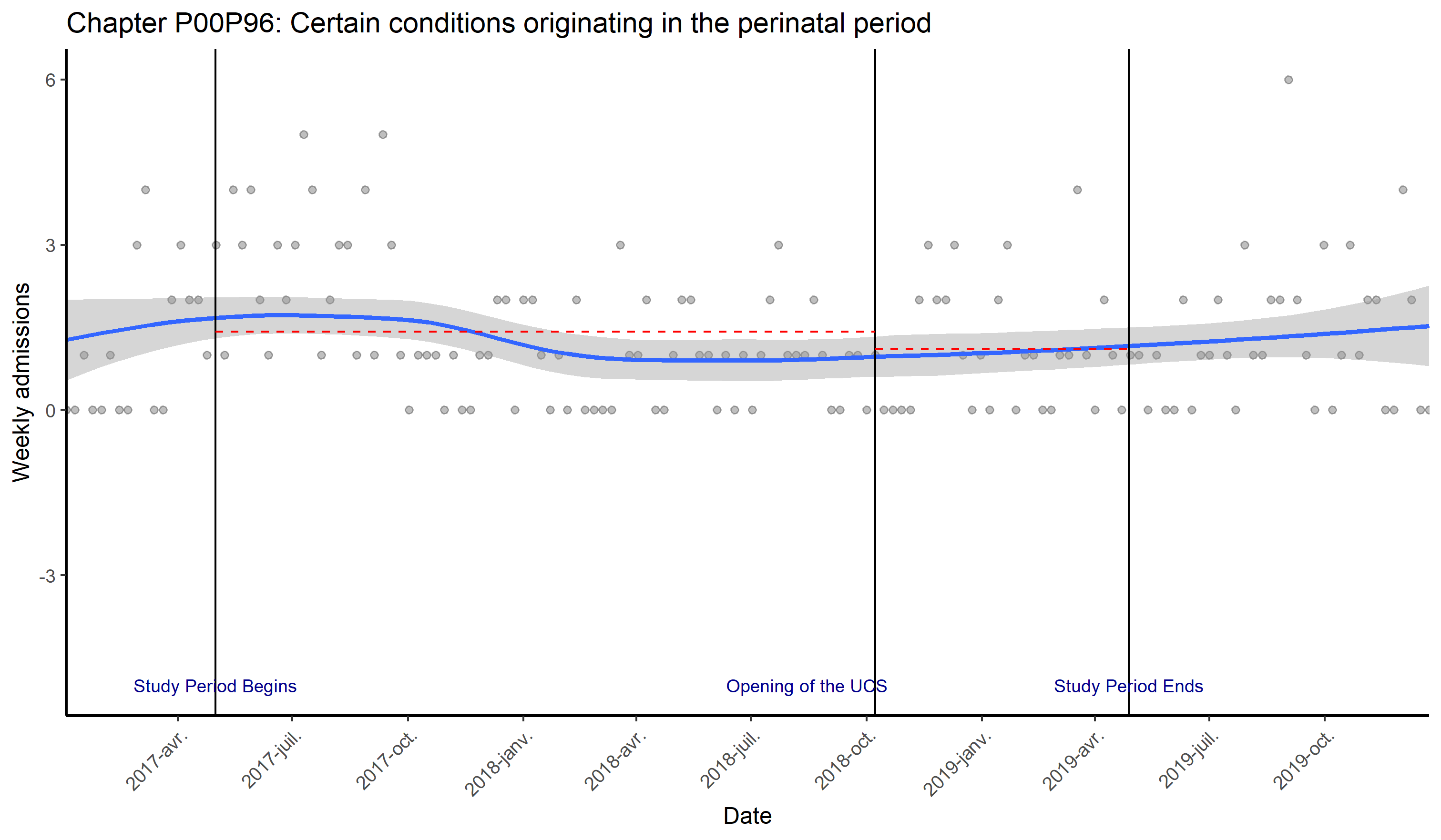** |
| **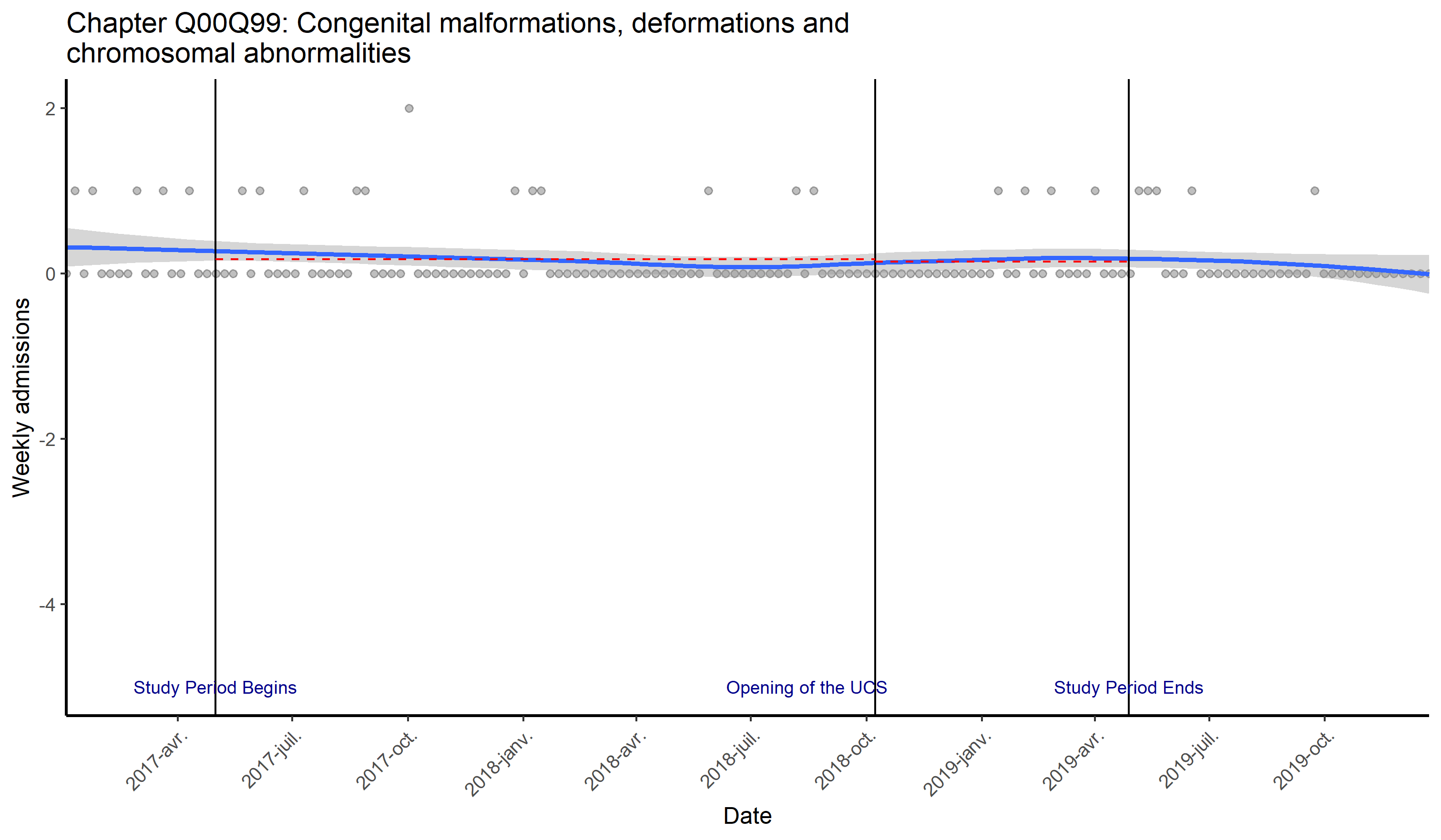** | **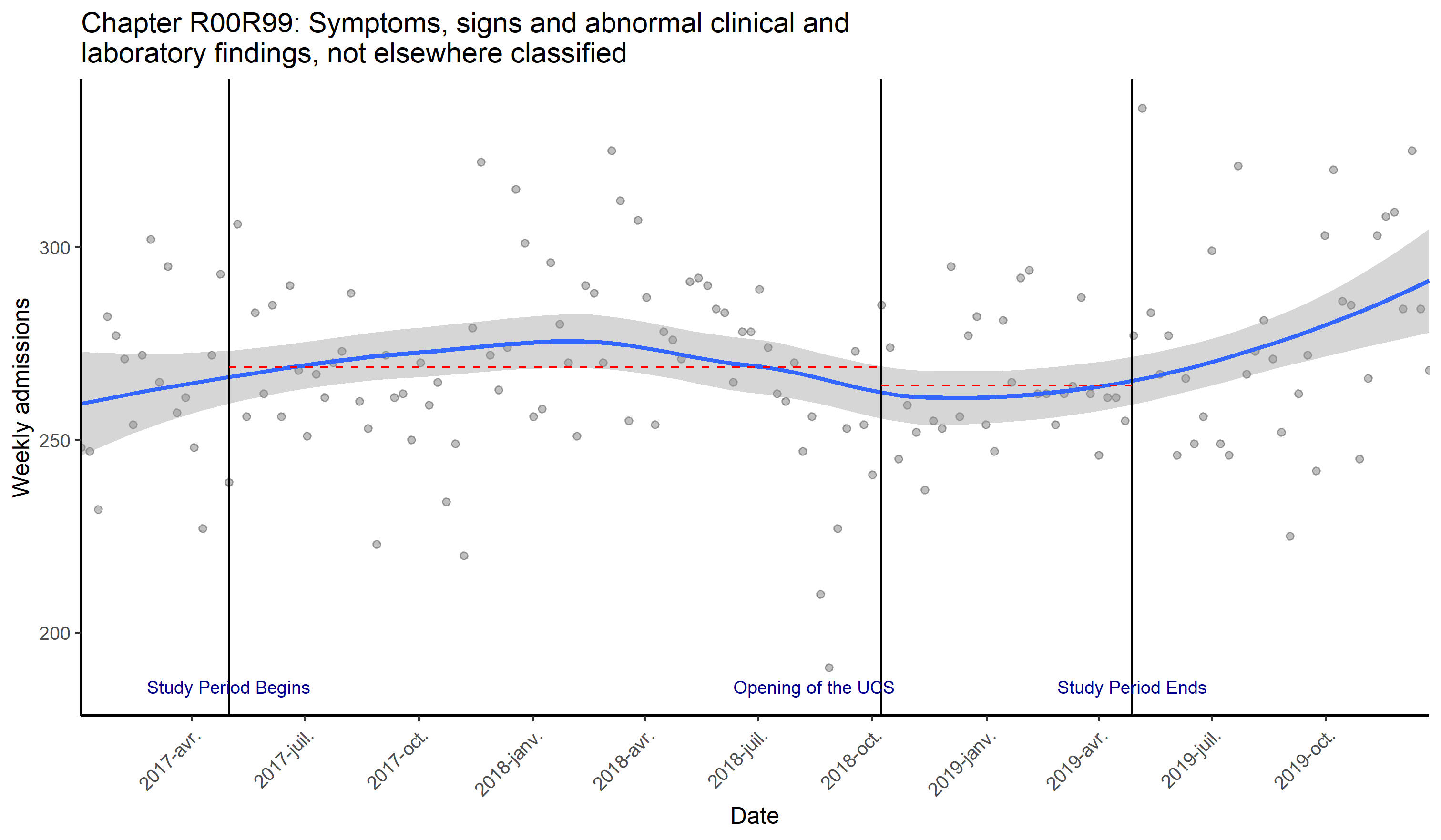** |
| **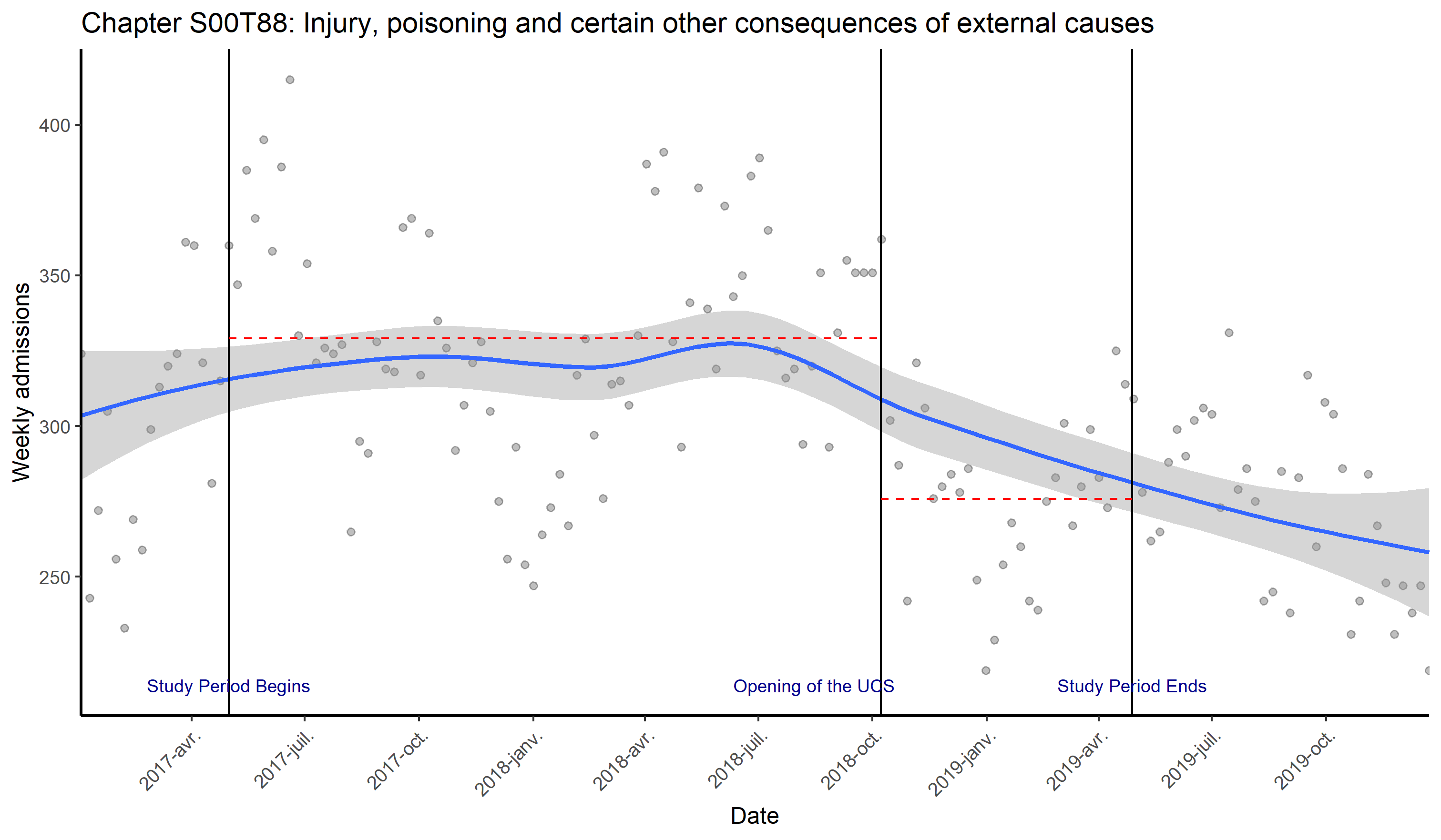** | **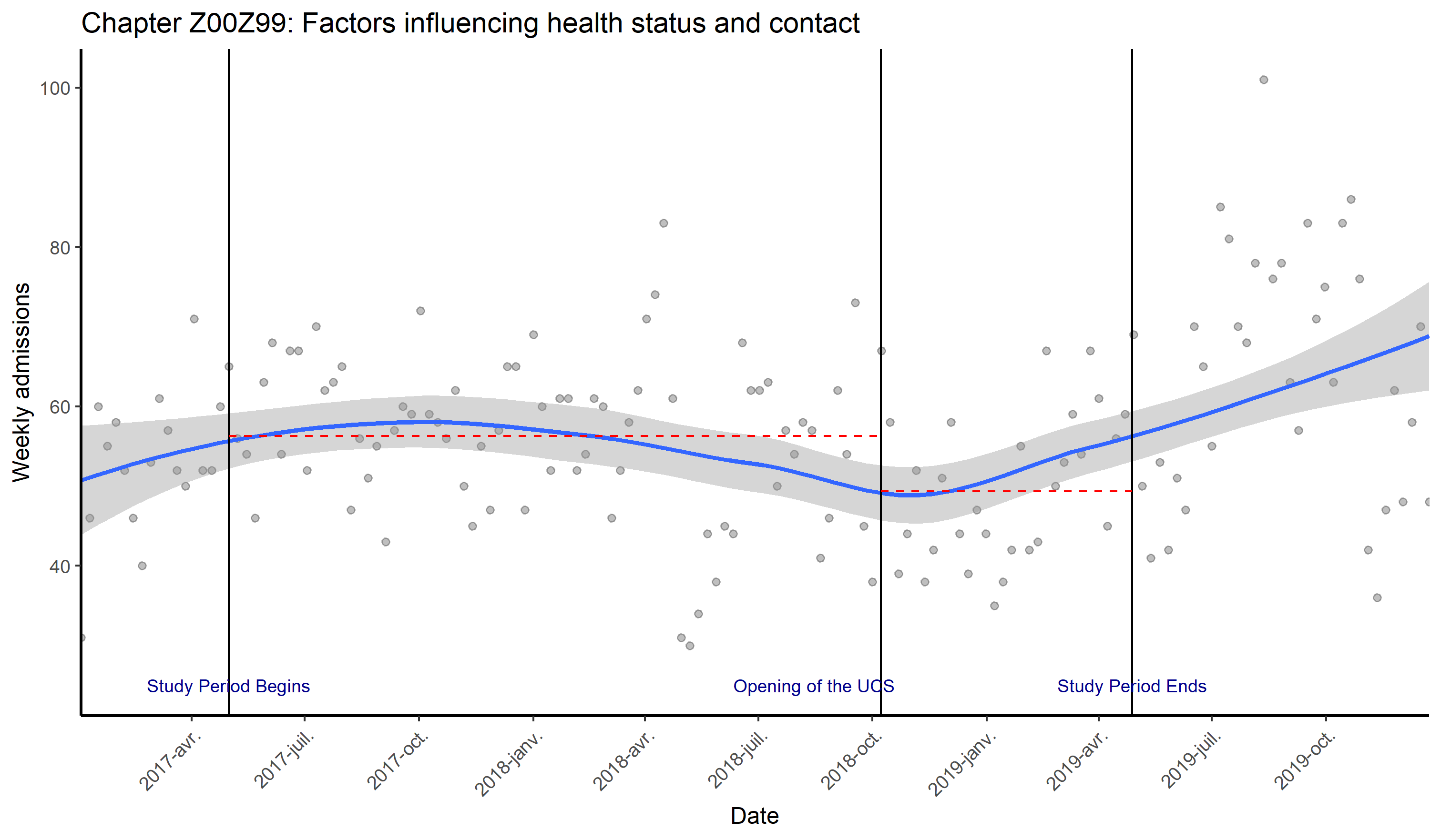** |

*Notes: The gray points are the observed weekly admissions, the blue line corresponds to local regression (loss) of the weekly admissions and the dotted red lines corresponds to the averages of weekly admissions before and after the opening of the UCS*

**Supplementary Figure S3.** Graphical evolutions in weekly admissions from 2017 to 2019 to illustrate the trends in weekly admissions for the 16-cluster structure and the ICD10 chapter classification structure
